# Performances of Adaptive MultiBLUP, Bayesian regressions, and weighted-GBLUP approaches for genomic predictions in Belgian Blue beef cattle

**DOI:** 10.1186/s12864-020-06921-3

**Published:** 2020-08-06

**Authors:** José Luis Gualdrón Duarte, Ann-Stephan Gori, Xavier Hubin, Daniela Lourenco, Carole Charlier, Ignacy Misztal, Tom Druet

**Affiliations:** 1 Unit of Animal Genomics, GIGA-R, 11 Avenue de l’Hôpital (B34), University of Liège, 4000 Liège, Belgium; 2 Innovation Department, Elevéo asbl and Inovéo, Awé Group, 5590 Ciney, Belgium; 3 Department of Animal and Dairy Science, University of Georgia, 425 River Rd, Athens, GA 30602 USA

**Keywords:** Genomic prediction, Genomic selection, Genome-wide association study, Bovine genomics, Beef cattle

## Abstract

**Background:**

Genomic selection has been successfully implemented in many livestock and crop species. The genomic best linear unbiased predictor (GBLUP) approach, assigning equal variance to all SNP effects, is one of the reference methods. When large-effect variants contribute to complex traits, it has been shown that genomic prediction methods that assign a higher variance to subsets of SNP effects can achieve higher prediction accuracy. We herein compared the efficiency of several such approaches, including the Adaptive MultiBLUP (AM-BLUP) that uses local genomic relationship matrices (GRM) to automatically identify and weight genomic regions with large effects, to predict genetic merit in Belgian Blue beef cattle.

**Results:**

We used a population of approximately 10,000 genotyped cows and their phenotypes for 14 traits, mostly related to muscular development and body dimensions. According to the trait, we found that 4 to 25% of the genetic variance could be associated with 2 to 12 genomic regions harbouring large-effect variants. Noteworthy, three previously identified recessive deleterious variants presented heterozygote advantage and were among the most significant SNPs for several traits. The AM-BLUP resulted in increased reliability of genomic predictions compared to GBLUP (+ 2%), but Bayesian methods proved more efficient (+ 3%). Overall, the reliability gains remained thus limited although higher gains were observed for skin thickness, a trait affected by two genomic regions having particularly large effects. Higher accuracies than those from the original AM-BLUP were achieved when applying the Bayesian Sparse Linear Mixed Model to pre-select groups of SNPs with large effects and subsequently use their estimated variance to build a weighted GRM. Finally, the single-step GBLUP performed best and could be further improved (+ 3% prediction accuracy) by using these weighted GRM.

**Conclusions:**

The AM-BLUP is an attractive method to automatically identify and weight genomic regions with large effects on complex traits. However, the method was less accurate than Bayesian methods. Overall, weighted methods achieved modest accuracy gains compared to GBLUP. Nevertheless, the computational efficiency of the AM-BLUP might be valuable at higher marker density, including with whole-genome sequencing data. Furthermore, weighted GRM are particularly useful to account for large variance loci in the single-step GBLUP.

## Background

Genomic selection [1] has been widely adopted in cattle and in other livestock species. The genomic best linear unbiased prediction or GBLUP [1, 2] and the single-step GBLUP [3, 4] are popular methods to estimate genomic breeding values (GEBV). They both rely on the construction of a genomic relationship matrix (GRM) using genotypes at a set of interrogated SNPs [2, 5] or even using whole-genome sequence data. These methods assume a polygenic model in which all the marker effects have equal variance and a small contribution to the total additive genetic variance. It has been previously shown that when large-effect variants contribute to complex traits, Bayesian methods that assign a higher variance to a subset of SNP effects can achieve higher prediction accuracy than GBLUP (e.g., [6]). For instance, models such as Bayes B [1], Bayes R [7] or the Bayesian sparse linear mixed model (BSLMM) proposed by Zhou et al. [8] assign SNPs to different classes based on the variance of their effect. These models have been shown to be effective in livestock species but also to predict complex traits in human data (e.g., [8, 9]). Alternatively, a number of studies proposed to weight the SNPs according to their effect or their variance to build the GRM in order to increase GBLUP prediction accuracy [10–12]. Different strategies have been proposed, relying on iterative schemes using SNP effects estimated from the GBLUP model [13–16] or on the posterior effects or variances obtained from some Bayesian methods [11, 12, 17]. It is also possible to directly estimate the variance associated with a single SNP or a subset of SNPs by fitting a specific GRM in a REML analysis (e.g., [18, 19]). Local GRM can for instance be used to identify genomic regions with significant contribution to genetic variation with the so-called Regional Heritability Mapping [20, 21]. In 2014, Speed and Balding [22] proposed and implemented the Adaptive MultiBLUP (AM-BLUP) approach for genomic prediction. This approach consists in using local GRMs, estimated with subsets of SNP, to identify regions of the genome significantly associated with the trait of interest. After this first association step, a REML approach is used to estimate the fraction of heritability explained by the significant regions. Doing so, they give more weights to regions including variants with larger effects and account for the genetic architecture of the trait. The method is computationally effective compared to some of the Bayesian models previously mentioned [22]. In addition, they showed that their approach achieved higher prediction accuracy than other methods, including BSLMM [22]. However, this approach was mostly tested for the prediction of human complex traits and the properties of the model remain unknown in livestock species.

The benefit of such approaches might increase in the near future as causative variants, or variants in high LD with those, are now frequently added to genotyping arrays to improve accuracy of genomic predictions (e.g., [23]). In addition, research projects are implemented to annotate genetic polymorphisms in livestock species [24] and that information might also be used to improve accuracy of genomic predictions [25].

A genomic selection program has been recently implemented in the Walloon region (Southern Belgium) for the Belgian Blue beef (BBB) cattle breed known for its extreme muscular development. As for other breeds and traits in cattle, several variants with large effects have been identified and characterized in that breed. The 11 bp deletion in the myostatin gene [26] is fixed and does not contribute any longer to genetic variation but other variants are still segregating in BBB (e.g., [27]). In particular, several variants causing genetic defects at homozygous state might have favourable effects in heterozygotes and be under balancing selection [28, 29]. The objective of the present study was to apply the AM-BLUP method for genomic predictions of complex traits in BBB cattle in order to study its properties in a livestock population. To do so, we compared the approach to state-of-the-art methods as GBLUP or BSLMM. In addition, we investigated a few strategies to improve the approach. We then evaluated whether the obtained weighted GRM could improve accuracy of single-step GBLUP (ssGBLUP) approaches. Because these methods also identify genomic regions or variants with larger effects, this study further provides insights into the genetic architecture of complex traits such as muscular development or height in that unique breed.

## Results

### Regions and SNPs significantly associated with traits

Application of the AM-BLUP results in the identification of regions significantly associated with each trait and consequently provides information on their genetic architecture. Similarly, the BSLMM identifies SNPs with a high probability to have a large effect on the traits (i.e., with a large posterior inclusion probability - **PIP**). We start thus by describing the regions identified by AM-BLUP (using 1 Mb chunks) and compare them to results from BSLMM or from more traditional GWAS approaches relying on LMM. Note that in a few cases, the AM-BLUP identified several regions close to each other that should possibly be merged as one larger region capturing more genetic variance. However, we preferred to keep the boundaries as defined by the default settings of the software (see Methods). The number of regions significantly associated ranged from 2 to 12 per trait (Table 1). These regions represented an average total length of 47.7 Mb (742.1 SNPs) per trait (Supp. File 1), and jointly captured from 4.3% (rib-shape) to 24.6% (height) of the additive genetic variance, with an average of 13.4% per trait and 2.6% per region. Identified regions accounted for 5% or more of the additive genetic variance of a trait in six instances. First, a region spanning from 36 to 47 Mb on BTA6 accounted for 6.8, 7.6 and 7.1% of the genetic variance of length, pelvis length and height, respectively, and encompassed the *NCAPG-LCORL* genes (non-SMC condensin I complex subunit G / ligand dependent nuclear receptor corepressor like) previously associated with height in cattle (e.g., [30, 31]) and in other species [32–34]. Although only two regions were associated with skin thickness, each one accounted for more than 5% of the genetic variance. Finally, a large region on BTA19 (from 38 to 56 Mb) explained 5.3% of the genetic variance of rump linear scoring. That region encompasses the loss-of-function mutation in the *MRC2* gene (mannose receptor C type 2) responsible for the crooked-tail syndrome [28] and previously associated with different muscular development traits [27]. This region overlapped with regions identified for nine other traits suggesting a highly pleiotropic region (affecting all traits but skin thickness, tail set and pelvis- and chest-width). Overall, the 72 associations could be condensed in 22 regions (regions were merged if they overlapped) affecting one to ten traits, confirming that some regions are pleiotropic and that multiple-trait approaches might be beneficial to increase power and mapping resolution.

**Table 1:** Summary of significant associations identified with the AM-BLUP approach, with the Bayesian Sparse Linear Mixed Model (BSLMM) and by genome-wide association study (GWAS). The table reports also the percentage of additive genetic variance (${\upsigma}_{\mathrm{g}}^2$) captured by the identified regions

Trait	Regions identified with AM-BLUP				BSLMM	GWAS	
	Number of regions	Proportion ${\upsigma}_{\mathrm{g}}^2$ (in %)	Total length of regions (Mb)	Number of SNPs	Number of SNPs with PIP > 0.5	Number of regions	Number of significant SNPs
Shoulder muscling	6	10.8	53.2	814	1	3	5
Top muscling	9	18.4	62.5	948	3	3	8
Skin thickness	2	12.9	19.4	234	1	2	15
Height	9	24.6	84.3	1301	9	10	42
Muscular development	12	19.4	104.7	1593	2	8	13
Rump	3	13.2	37.8	595	2	5	14
Length	9	22.2	59.4	883	6	5	12
Chest width	2	6.1	14.2	230	3	2	2
Pelvis width	2	6.0	18.0	300	2	3	5
Rib shape	2	4.3	19.8	291	1	3	3
Pelvis length	4	18.2	35.8	595	4	3	5
Tail set	2	5.7	20.7	316	0	3	3
Buttock muscling (side)	5	11.0	64.1	950	1	6	20
Buttock muscling (rear)	5	15.1	73.8	1048	3	4	5

**Fig. 1:**
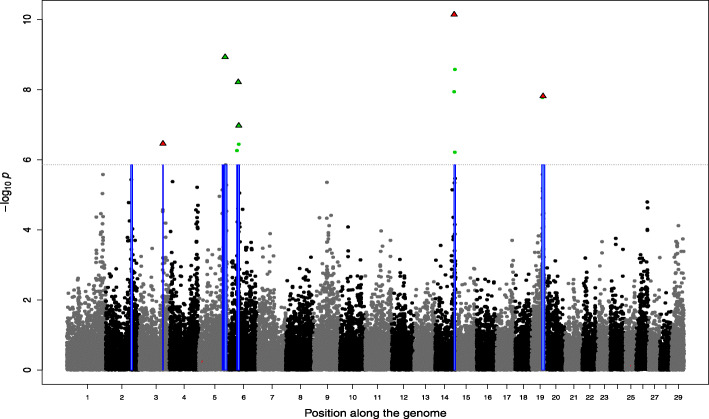
Genome-wide association study for length. The Manhattan plot represents the significance of SNP associations estimated with linear-mixed models. The significant SNP are represented in green. Significant regions identified with the Adaptive MultiBLUP approach are represented with a blue box. SNP with a posterior inclusion probability (PIP) larger than 0.5 are identified with triangles. The PIP were estimated with BSLMM. The five known coding variants previously identified for their association with specific phenotypes (see Table 2) are in red. The horizontal dashed line indicates the significance threshold

**Fig. 2:**
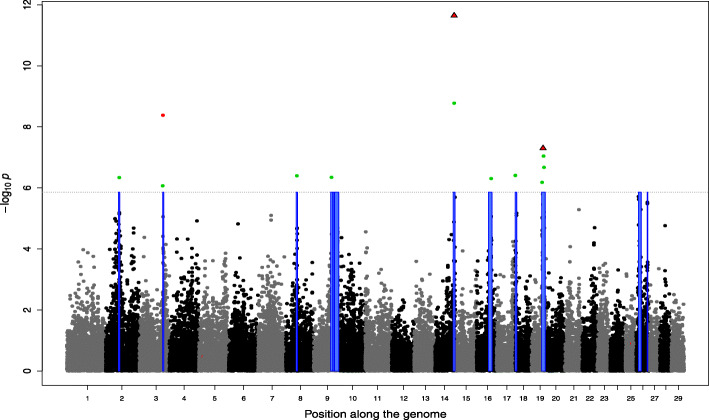
Genome-wide association study for muscular development. The Manhattan plot represents the significance of SNP associations estimated with linear-mixed models. The significant SNP are represented in green. Significant regions identified with the Adaptive MultiBLUP approach are represented with a blue box. SNP with a posterior inclusion probability (PIP) larger than 0.5 are identified with triangles. The PIP were estimated with BSLMM. The five known coding variants previously identified for their association with specific phenotypes (see Table 2) are in red. The horizontal dashed line indicates the significance threshold

As a matter of comparison, the number of SNPs with a PIP (obtained with BSLMM) higher than 0.5 ranged from 0 to 9 (Table 1) per trait, for a total of 38 hits across all traits (2.7 per trait on average). This corresponded to 16 unique SNPs and the number of traits associated with each of these SNPs ranged from 1 to 9 (Suppl. File 1). Among these SNPs, five known variants with coding effects (missense, splice-site and loss-of-function variants) accounted for more than 50% of the hits (Supp. File 1). More precisely, the loss-of-function mutation in *MRC2* [28] segregating at 4% in our sample was associated with 7 traits, the splice-site variant in *RNF11* (ring finger protein 11) causing dwarfism [29] and segregating at 6% had PIP > 0.5 for 2 traits and a missense variant in *WWP1* (WW domain containing E3 ubiquitin protein ligase 1) [35] segregating at 14% was found for 9 traits whereas another less frequent (1%) missense variant in *ATP2A1* (ATPase sarcoplasmic/endoplasmic reticulum Ca2+ transporting 1) [36] causing congenital muscular dystonia 1 (CMD1) had a large effect on linear score for rump. These four variants are known to have deleterious effects at homozygous states and at least two of them have been shown to be under balancing selection [28, 29]. These variants affect mostly size (e.g., shorter animals) and muscular development traits (higher muscularity). Finally, the roan coat color [37], a missense variant in *KITLG* (KIT ligand) on BTA5, was found associated with skin thickness. When the PIP were cumulated over windows of 25 SNPs (Supp. File 1), 1 to 12 windows were identified per trait (on average 11.1 SNPs per trait). This represents only 8 new regions (+ 21%) compared to selection based on individual SNPs PIP. With the LMM-based GWAS, 11.3 significant SNPs were detected on average per trait, corresponding to 2 to 10 regions per trait (Table 1; Supp. File 1). As expected, the 5 variants previously mentioned were found significantly associated with several traits (Table 2). Overall, the overlap among the AM-BLUP, the BSLMM and the LMM-based GWAS was relatively high as illustrated for length and muscular development in Figs. 1 and 2 respectively, and for other traits in Supp. File 2. The majority of SNPs with a PIP > 0.5 (35 out of 38), or the window of 25 SNPs with cumulative PIP > 0.5 (37 out of 46), were inside the regions identified by the AM-BLUP (for 12 traits, this last proportion was 100% - Supp. File 1). Similarly, the significantly associated SNPs identified with the LMM approach were also in majority located within large-variance regions found by the AM-BLUP (86%). This proportion increased to 97 and 100% for SNP with *p*-values below 1e-8 and 1e-10.

**Table 2:** Association between five coding variants and different traits. The five variants have been previously associated with specific phenotypes (coat colour or recessive genetic defects) and the corresponding gene names are indicated in the table. The associations are reported in terms of *p*-values and posterior inclusion probability (PIP) estimated with the Bayesian Sparse Linear Mixed Model

Gene	RNF11 [29]		KITLG [37]		WWP1 [35]		MRC2 [28]		ATAP2A1 [36]	
Phenotype	Dwarfism		Roan coat colour		Fitness		Crooked-tail syndrome		Congenital Muscular dystonia 1	
Trait	PIP	*p-value*	PIP	*p-value*	PIP	*p-value*	PIP	*p-value*	PIP	*p-value*
Shoulder muscling	0.01	9.6e-5	0	0.78	1	1.4e-11	0.02	1.0e-4	0	0.39
Top muscling	0.92	3.8e-11	0	0.25	1	1.2e-12	0.91	2.4e-6	0	0.34
Skin thickness	0	0.16	1	5.4e-12	0	2.1e-1	0	0.57	0	0.09
Height	0.28	4.2e-8	0	0.74	1	1.6e-11	0.68	1.2e-12	0.01	0.42
Muscular development	0.37	4.1e-9	0	0.34	1	2.3e-12	0.81	5.0e-8	0	0.13
Rump	0	1.7e-2	0	0.50	0	6.7e-2	0.97	1.6e-13	1	2.0e-8
Length	0.62	3.5e-7	0	0.59	1	7.2e-11	0.75	1.5e-8	0	0.76
Chest width	0.01	6.5e-3	0	0.45	0.76	7.4e-6	0.03	3.8e-5	0	0.39
Pelvis width	0	5.7e-2	0	0.56	0.91	5.3e-5	0.03	3.2e-5	0	0.46
Rib shape	0	4.0e-4	0	0.31	0	3.4e-2	1	2.0e-10	0	0.94
Pelvis length	0.03	2.5e-3	0	0.24	0.99	5.8e-8	0.42	2.6e-9	0	0.23
Tail set	0	1.0e-2	0	0.15	0.01	4.8e-2	0	0.98	0	0.01
Buttock muscling (side)	0.02	6.7e-6	0	0.60	0.23	2.9e-3	0.01	3.1e-3	0.29	7.6e-5
Buttock muscling (rear)	0.01	4.3e-4	0	0.10	1	2.8e-11	0.54	8.0e-8	0	0.34

**Fig. 3:**
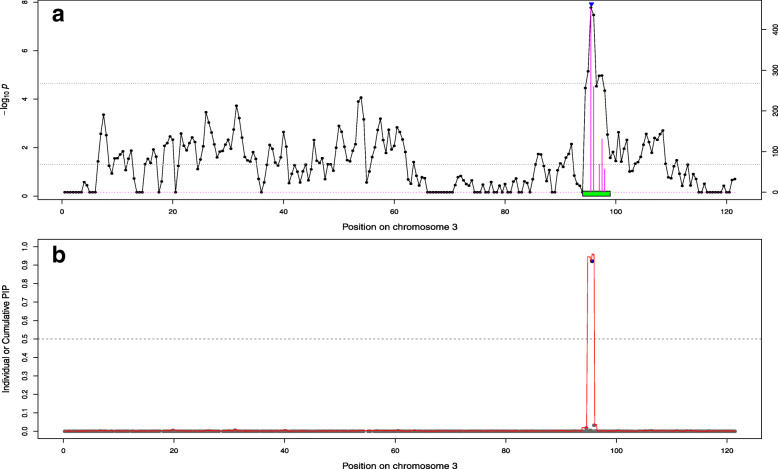
Association curves for top muscling on chromosome 3. **a**) Association with the Adaptive MultiBLUP approach. The line indicates the significance of association with 1 Mb chunks. The upper horizontal dashed line indicates the significance threshold obtained by permutations, the lower horizontal line is the lower limit to include chunks in a significant region whereas the green box represent the boundaries of the region used in the bootstrapping. The height of barplots indicates the number of time the segment was best in a bootstrapping sample. The blue triangle represents the position of the variant in *RNF11* associated with dwarfism. **b**) Association with BSLMM. The red curve represents the cumulated posterior inclusion probability (PIP) of 25 SNP windows whereas the grey points indicate individual SNP PIP

**Fig. 4:**
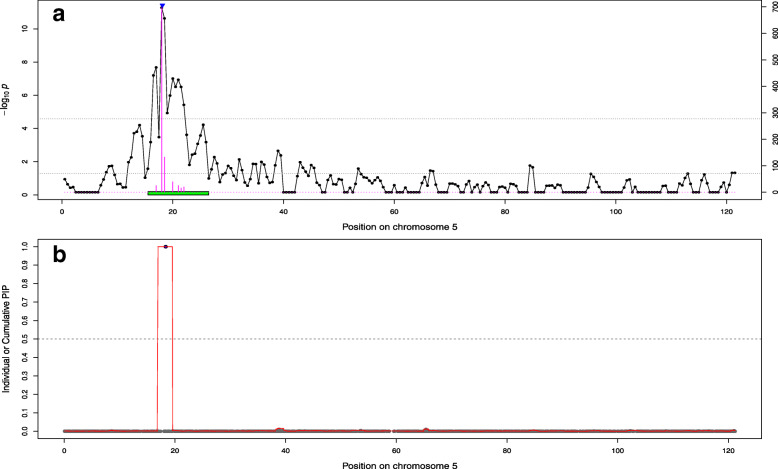
Association curves for skin thickness on chromosome **5. a**) Association with the Adaptive MultiBLUP approach. The line indicates the significance of association with 1 Mb chunks. The upper horizontal dashed line indicates the significance threshold obtained by permutations, the lower horizontal line is the lower limit to include chunks in a significant region whereas the green box represent the boundaries of the region used in the bootstrapping. The height of barplots indicates the number of time the segment was best in a bootstrapping sample. The blue triangle represents the position of the variant in *KITLG* associated with roan coat colour. **b**) Association with BSLMM. The red curve represents the cumulated posterior inclusion probability (PIP) of 25 SNP windows whereas the grey points indicate individual SNP PIP

**Fig. 5:**
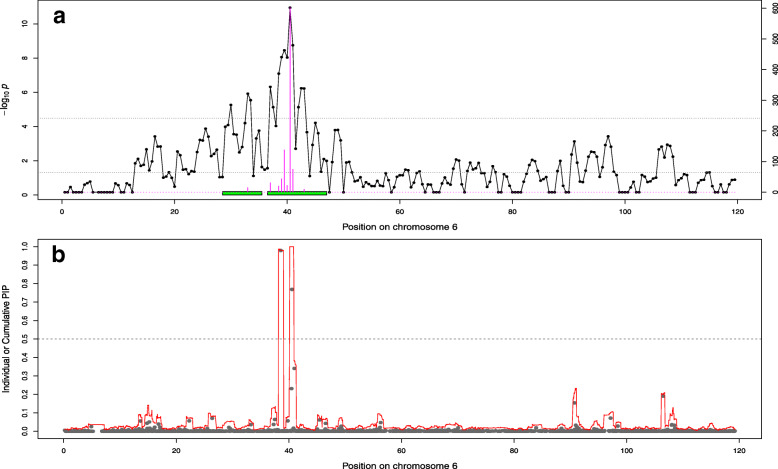
Association curves for height on chromosome 6. **a**) Association with the Adaptive MultiBLUP approach. The line indicates the significance of association with 1 Mb chunks. The upper horizontal dashed line indicates the significance threshold obtained by permutations, the lower horizontal line is the lower limit to include chunks in a significant region whereas the green box represent the boundaries of the region used in the bootstrapping. The height of barplots indicates the number of time the segment was best in a bootstrapping sample. **b**) Association with BSLMM. The red curve represents the cumulated posterior inclusion probability (PIP) of 25 SNP windows whereas the grey points indicate individual SNP PIP

We then used permutations techniques to define significance thresholds and obtained higher values (less conservative) compared to the default threshold (they ranged from 1.19 × 10^− 5^ to 3.26 × 10^− 5^). As a result, more significant regions were detected although several neighboring regions were sometimes merged into a single one (Table 3 and Supp. File 1). In addition, we used bootstrapping techniques to optimize the definition of confidence intervals (**CI**). This allowed to obtain smaller regions than the use of default thresholds (on average 6.6 Mb vs. 9.3 Mb or 104.7 SNPs vs. 140.3 SNPs). Nevertheless, the total genetic variance captured by these smaller regions was on average as high as with the default option (the proportion increased from 13.4 to 15.5%) and the average genetic variance captured per SNP from selected regions increased (from 0.019 to 0.026% per SNPs). Interestingly, the five coding variants previously identified were always included in the bootstrap confidence interval (for the 18 cases for which the coding variant and the surrounding region were simultaneously identified with BSLMM and the AM-BLUP). In most cases, they were even located in the SNP window identified most frequently as best across bootstraps (16 out of 18). To illustrate this, for a QTL on chromosome 3 that affects top muscling and is associated to the variant in *RNF11*, we plotted the significance of 1 Mb chunks, the boundaries of the region selected with AM-BLUP approach, the frequency a chunk was identified as best in the bootstrap samples (and the resulting confidence intervals), and the PIP of individual SNPs or the cumulative PIP from 25 SNPs windows (Fig. 3). A similar picture is available for the QTL affecting skin thickness on chromosome 5 and associated with the variant in *KITLG* (Fig. 4). In both cases, we observed a good overlap between SNPs / regions identified by different methods. Similar figures are available for all QTLs in Supp. File 3. Such fine-scale inspection of BSLMM results also suggests that the region on BTA6 affecting height and length might harbor two QTLs since we observed two distinct windows with PIP close to 1 (Fig. 5, Supp. File 3). This might also be the case for the association between height and the region on BTA19 encompassing *MRC2,* although with less evidence because both windows do not reach values of 1 (Supp. File 3).

**Table 3:** Summary of regions identified with alternative strategies: 1) the AM-BLUP approach when significance thresholds are defined by permutations and the confidence interval with a bootstrapping approach (BAM-BLUP) and 2) windows of 25 SNPs having a cumulative posterior inclusion probability (PIP) larger than 0.50 (WPIP-GBLUP). The table reports also the percentage of additive genetic variance (${\upsigma}_{\mathrm{g}}^2$) captured by the identified regions

Trait	BAM-BLUP				WPIP-GBLUP			
	Number of regions	Total length of regions (Mb)	Proportion ${\upsigma}_{\mathrm{g}}^2$ (in %)	Number of SNPs	Number of regions	Total length of regions (Mb)	Proportion ${\upsigma}_{\mathrm{g}}^2$ (in %)	Number of SNPs
Shoulder muscling	6	40.5	10.7	636	1	0.0	3.3	1
Top muscling	9	45.5	19.4	714	3	0.4	6.5	4
Skin thickness	2	15.5	14.5	225	1	0.0	6.8	1
Height	14	95.0	32.8	1546	12	11.7	18.7	46
Muscular development	7	57.0	17.0	909	2	0.6	4.7	3
Rump	4	26.0	14.0	419	2	0.0	4.0	2
Length	8	38.5	25.3	599	6	4.1	12.8	15
Chest width	5	32.5	11.9	488	1	1.1	1.9	3
Pelvis width	4	26.0	9.0	414	6	7.1	11.0	41
Rib shape	3	22.0	7.2	357	1	0.0	3.1	1
Pelvis length	4	25.0	18.0	405	5	2.4	10.0	17
Tail-set	3	15.5	8.5	252	1	0.5	5.2	10
Buttock muscling (side)	7	47.5	14.2	740	2	1.9	2.6	9
Buttock muscling (rear)	4	43.5	14.1	668	3	0.0	6.2	3

Finally, we also selected large-variance regions according to the cumulative PIP of 25 SNPs windows. With this alternative strategy, fewer regions were selected (Table 3). These regions contained also fewer SNPs (11.1 per trait and 3.4 per window) because we selected only SNPs with significant contribution to the window PIP with that approach (see methods), whereas low PIP SNPs within the window were filtered out. Overall, with this strategy, the total amount of genetic variance (estimated with the GREML approach implemented in LDAK) captured by large-variance regions decreased, on average, to 6.9%. Interestingly, the variance captured per SNP was much larger, increasing from 0.019% to 0.618% of the genetic variance.

### Reliability of genomic predictions

**Fig. 6:**
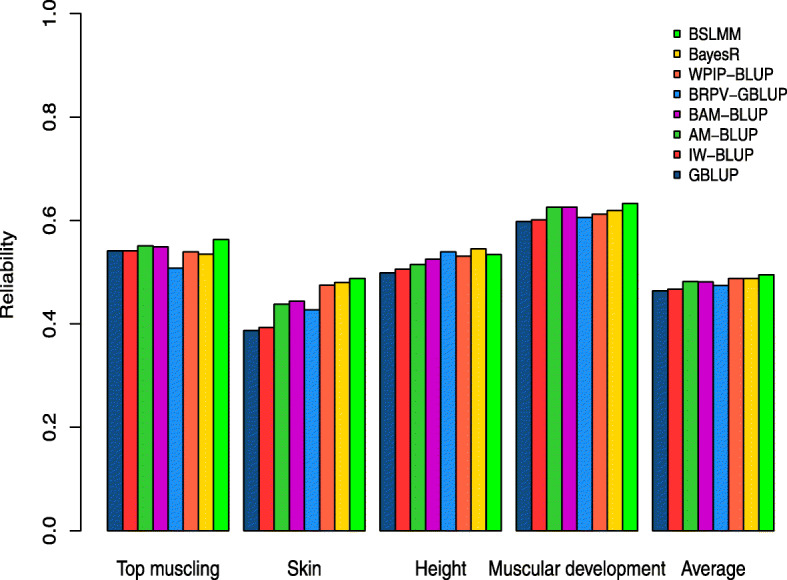
Individual and average reliabilities of predicted genomic EBVs in Belgian Blue Beef cattle using different methods and SNP weighting strategies. The reliabilities are reported for 4 individual traits (top muscling, skin thickness, height and muscular development) and for the average of 14 traits related to muscular development and conformation. The genomic prediction methods are standardized GBLUP, iterative weighted GBLUP (IW-GBLUP), Adaptive MultiBLUP (AM-BLUP), Bootstrap Adaptive MultiBLUP (BAM-BLUP), BayesR Posterior Variance GBLUP (BRPV-GBLUP), Window Posterior Inclusion Probability GBLUP (WPIP-GBLUP), BayesR and Bayesian Sparse Linear Mixed Model (BSLMM)

Reliability of different genomic prediction methods are reported in Table 4 (see also Fig. 6 for results for a subset of traits). The AM-BLUP approach slightly improved reliabilities compared to the standardized GBLUP approach (average values increased from 0.46 to 0.48), except for rib shape. Application of BSLMM resulted in better results (0.49 on average), with increased accuracy for all traits (except for rib shape again). Nevertheless, the overall increase in reliability was modest, although a few traits, e.g., skin thickness, had a larger benefit. BayesR showed results (0.49) that were similar to those from BSLMM, albeit slightly lower. We also tested two alternative weighted GBLUP strategies that did not perform better than the two Bayesian approaches. First, the iterative procedure using SNP solutions as described in Wang et al. [13] promoted a slight increase in reliabilities (0.47) compared to the standardized GBLUP. When posterior variances obtained from BayesR were used as weights in GBLUP, the performance was on average better than the standardized GBLUP (0.47 average reliability), but not consistently across traits. Standardized genotypes were used in all these methods, and therefore, we compared accuracy with a standardized GBLUP that gives more weight to SNP with low MAF (see Methods). The reliability with a GBLUP assuming equal variance of SNP effects was slightly better, with an average of 0.47 across 14 traits (Table 4).

**Table 4:** Reliabilities of predicted genomic EBVs for conformation and muscular traits in Belgian Blue beef cattle using different methods and SNP weighting strategies

Trait	GBLUP	Standardized GBLUP	AM-BLUP^a^	BAM-BLUP^b^	BSLMM^c^	BayesR	WPIP-GBLUP^d^	IW-GBLUP^e^	BRPV-GBLUP^f^
Shoulder muscling	0.57	0.56	0.58	0.58	0.59	0.57	0.58	0.56	0.55
Top muscling	0.56	0.54	0.55	0.55	0.56	0.53	0.54	0.54	0.51
Skin thickness	0.39	0.39	0.44	0.44	0.49	0.48	0.48	0.39	0.43
Height	0.49	0.50	0.52	0.52	0.53	0.54	0.53	0.51	0.54
Muscular development	0.62	0.60	0.63	0.63	0.63	0.62	0.61	0.60	0.61
Rump	0.44	0.44	0.47	0.48	0.47	0.46	0.47	0.44	0.45
Length	0.37	0.37	0.38	0.38	0.40	0.40	0.41	0.37	0.39
Chest width	0.41	0.40	0.41	0.39	0.44	0.43	0.40	0.41	0.44
Pelvis width	0.32	0.31	0.33	0.34	0.35	0.34	0.35	0.31	0.33
Rib shape	0.49	0.51	0.51	0.51	0.50	0.50	0.51	0.51	0.49
Pelvis length	0.30	0.30	0.32	0.32	0.32	0.32	0.34	0.30	0.31
Tail set	0.49	0.48	0.46	0.45	0.49	0.47	0.48	0.48	0.46
Buttock muscling (side)	0.54	0.53	0.54	0.53	0.55	0.54	0.53	0.53	0.54
Buttock muscling (rear)	0.58	0.57	0.60	0.60	0.61	0.61	0.59	0.57	0.60
**Average**	**0.47**	**0.46**	**0.48**	**0.48**	**0.49**	**0.49**	**0.49**	**0.47**	**0.47**

^a^Adaptive MultiBLUP

^b^Bootstrap Adaptive MultiBLUP

^c^Bayesian sparse linear mixed model (BSLMM)

^d^Window Posterior Inclusion Probability GBLUP

^e^iterative weighted GBLUP (IW-GBLUP)

^f^BayesR Posterior Variance GBLUP (BRPV-GBLUP)

When parameters of the AM-BLUP were optimized with permutation and bootstrapping strategies, resulting reliabilities remained close to those obtained with default parameters (0.48). However, this result was realized with fewer SNPs in the large variance regions. When regions were selected based on cumulative PIP in 25 SNPs windows, higher reliabilities (0.49) were achieved with even fewer SNPs, close to obtained with the Bayesian methods.

Methods were also compared in terms of mean-square error (MSE) and bias (see Supp. File 1). These metrics resulted in even lower differences among methods. The methods with highest reliability had lowest MSE, and vice versa. The bias in the scale of genomic prediction was measured as the deviation from 1 of the regression coefficients of TD on genomic predictions. In general, this regression coefficient was lower than 1 indicating inflation, and values were similar across all methods, with the exception of BRPV-GBLUP that presented larger deviations from 1 (and IW-GBLUP to a lesser extent).

### Reliability of genomic predictions with single-step GBLUP

Application of the ssGBLUP model resulted in higher reliabilities (0.52 on average for the tested traits with the standardized ssGBLUP), providing clearer benefits compared to other tested methods (Table 5). Differences between ssGBLUP and standardized GBLUP were twice as large as those between Bayesian methods and standardized GBLUP, indicating that the main gain in the present situation might come from adding ungenotyped individuals and not from optimally weighting SNPs (with our genotyped sample and marker panel).

**Table 5:** Reliabilities of predicted genomic EBVs for conformation and muscular traits in Belgian Blue beef cattle using single-step GBLUP with different weighting strategies

Trait	Weighting strategy				
	Unweighted	Unweighted standardized^a^	AM-BLUP^b^	IW-GBLUP^c^	WPIP-GBLUP^d^
Shoulder muscling	0.65	0.64	0.66	0.64	0.66
Top muscling	0.60	0.58	0.59	0.58	0.58
Skin thickness	0.45	0.44	0.51	0.45	0.52
Height	0.57	0.58	0.59	0.58	0.61
Muscular development	0.64	0.63	0.66	0.63	0.64
Rump	0.58	0.58	0.61	0.59	0.60
Length	0.41	0.42	0.44	0.42	0.46
Chest width	0.48	0.47	0.48	0.47	0.47
Pelvis width	0.41	0.39	0.41	0.39	0.43
Rib shape	0.56	0.56	0.57	0.56	0.57
Pelvis length	0.36	0.36	0.38	0.36	0.40
Tail set	0.55	0.53	0.53	0.53	0.54
Buttock muscling (side)	0.59	0.58	0.59	0.58	0.58
Buttock muscling (rear)	0.60	0.59	0.62	0.59	0.61
**Average**	**0.53**	**0.52**	**0.55**	**0.53**	**0.55**

^a^Unweighted ssGBLUP using standardized genotypes

^b^Adaptive MultiBLUP

^c^iterative weighted GBLUP (IW-GBLUP)

^d^Window Posterior Inclusion Probability GBLUP

Nevertheless, ssGBLUP can also incorporate weighted GRM constructed with all the methods previously tested (e.g., [13, 16, 38]). For instance, we implemented ssGBLUP using the GRM weighted with the AM-BLUP model, with the iterative procedure implemented in postGSf90 [39], and with the strategy relying on the selection of windows with large cumulative PIP (combined with a subsequent estimation of their specific variance by REML). In all cases, the reliability of predictions (averaged across the 14 traits) from ssGBLUP increased, to 0.53 with the iterative procedure and to 0.55 with both the AM-BLUP GRM and the PIP-based approach (Table 5).

In terms of MSE, ssGBLUP approaches had slightly lower MSE than previous approaches, and ssGBLUP with weighted GRM had lower MSE than unweighted standardized ssGBLUP, confirming observations based on reliability of genomic predictions. Regarding bias in scaling, ssGBLUP approaches had also on average regression coefficients closer to 1, although the differences were limited.

## Discussion

We herein compared several genomic prediction methods that differ in their SNP weighting strategy (e.g., uniform weighting, window weighting, and individual SNP weights). Some of these methods also provided information on genomic regions significantly associated with traits of interest. In the vast majority of cases, LMM-based association studies, Bayesian approaches such as BSLMM or methods based on association between chunks of SNPs (similar to regional heritability mapping approaches) captured the same variants / regions and provided similar description of the genetic architecture of the traits. We analyzed here mostly traits related to muscular development scoring or to animal dimensions. Overall, all traits appeared polygenic as 75 to 95% of the genetic variance was captured by the background random polygenic effect (composed of many polymorphisms of small effect), whereas each QTL region captured approximately 2 to 4% of the genetic variance. Traits such as length or height presented the highest number of significant associations (around 10) whereas for individual muscular scoring measures, the number varied from around five for buttock muscularity (profile and rear views) or shoulder muscling to nine for top muscling. Note that the perception of certain muscular development scores might be influenced by animal’s height (i.e., these traits are correlated). Height is a trait selected in several cattle breeds and domestic species, and this for many generations (see for instance discussion in Bouwman et al. [31]). Among the variants associated with height or length, we found several regions also associated with height in other breeds [31] and hence potentially associated with old variants (although different mutations might also segregate in different breeds). The *NCAPG-LCORL* region (on chromosome 6), associated with the largest fraction of genetic variance, has been associated with height in several cattle breeds (see review by Takasuga [30]) and also in other species including human, dogs and horses [32–34]. It has also been associated with large selective sweeps in cattle (e.g., [31, 40]). The QTL region on BTA5 matches the highly significant association in Bouwman et al. [31] linked to *CCND2* (cyclin D2) whereas the BTA26 QTL corresponds to a hit in the vicinity of *PLEKHA1* (pleckstrin homology domain containing A1) [31]. For these three cases, the SNP with high PIP was less than 250 kb from the top SNP in Bouwman et al. [31] despite that we used a medium density array. Other QTL regions (merged chunks at the end of BTA2 and BTA4 or the Hapmap38123-BTA-72477 and rs109090354 markers on BTA2 and BTA7) might be related to other hits from the meta-analysis from Bouwman et al. [31] but with less evidence. Beside these variants that may be from pre-breed formation, three other highly significant variants in *RNF11*, *WWP1* and *MRC2* [28, 29, 35] are recent variants specific to the BBB breed. These variants have also an effect on muscular development traits and have recessive deleterious effects while presenting selective advantages at heterozygous state (e.g., [28]). According to the trait, they account from 1.0 to 5.4% of the genetic variance. High contribution of the *MRC2* variant to genetic variance of muscular development traits has been previously reported [27] but its allele frequency has been since decreasing as a result of selection implemented against carrier bulls as for the variants in *RNF11* and *ATP2A1*. The *WWP1* variant presents now higher significance levels and is more frequent in the population (14%). Compared to QTLs observed in other breeds, these variants would correspond to a more recent and extreme selection for increased muscularity and shorter animals. Finally, skin thickness presented an interesting architecture with only two significant regions but each one capturing more than 5% of the genetic variance. Despite these different architectures, we did not observe a clear relationship between the total percentage of genetic variance explained by the QTL or the number of QTL and the benefits of weighting these regions (measured as increased reliability). It is noteworthy to mention that prediction of skin thickness, a trait for which the variance captured by each of the 2 significant QTL was maximal, responded best to weighting strategies.

Although the regions identified by the tested methods were highly similar, the underlying variants were exploited differently, resulting in variable gains in accuracy. When considering only genotyped individuals, Bayesian approaches relying on mixture of distributions (including distributions for large SNP effects) achieved the highest accuracies, consistently better than the GBLUP approach. This agrees with previous reports showing higher accuracies with similar Bayesian approaches when large effect variants are segregating in the population (e.g., [6, 11]). On average, none of the weighted GBLUP achieved better results than BSLMM. Calus et al. [12] and Su et al. [11] obtained nevertheless similar accuracies between Bayesian variable selection models and weighted GBLUP for dairy traits. The AM-BLUP resulted in higher reliability than standardized GBLUP but it was most often less accurate than BSLMM, contrary to comparisons performed with human complex traits by Speed and Balding [22]. The data structures were however different since LD extends at shorter range in human populations and higher density maps are used, allowing to work with smaller chunks of markers. The number and size of independent chromosome segments in both genomes are also expected to be very different [41]. For instance, a cattle population would typically contain 10,000 to 15,000 segments that are 200 kb long, whereas in humans, a genome of similar physical size, segments would be only 2 kb long [41]. The lower accuracy of the AM-BLUP approach compared to BSLMM in the present study might be due to the fact that the AM-BLUP selected large regions (several Mb, containing multiple segments). A large variance is then assigned to many SNPs, or segments, although only a few of them (eventually one) have truly larger effects. Conversely, Bayesian methods relying on Gibbs sampling usually estimate SNP effects sequentially, and when a specific SNP is assigned a large effect, adjacent effects may automatically be assigned a small variance (BSLMM) or no effect (BayesR). Therefore, these methods work well for traits where causative SNP with large effects are identified and present in the data. We tried to address that issue associated with the AM-BLUP by optimizing significance thresholds and by defining CI through bootstrapping. This allowed to achieve the same accuracy with less SNPs, but with reliability levels still below BSLMM. That refined strategy still assigned the same variance to all SNPs within a chunk, including SNPs without effect. We then used the PIP values estimated by BSLMM to identify SNPs (or group of SNPs) with higher probability to have a large effect and subsequently estimated their variance with a REML procedure. That strategy improved prediction accuracy (still slightly below BSLMM) and strongly reduced the number of large effect SNPs. Overall, these results suggest that it is essential to identify unambiguously large-effect variants and to exclude neighboring SNPs with small effects. Inclusion of functional annotation might help to identify such SNPs [25, 42] and efforts to put putative causal variants on custom genotyping arrays would also be beneficial in that sense [23]. Here, we observed that such known causal variants present on the array were clearly identified by the BSLMM approach and that their weighting resulted in increased prediction accuracy. When the custom SNPs were not included in the analysis, the average reliabilities across 14 traits were 1% lower with the standardized GBLUP and 2% lower with the BSLMM. This illustrates that both approaches benefited from the inclusion of the custom SNPs, but that the BSLMM exploited them better. Note also that these custom SNPs were not selected for their strong association with phenotypes, but contained variants predicted to have potential deleterious effects such as loss-of-function variants [35].

Although individual SNP weighting methods avoid some of the caveats associated with chunk-based strategies, the iterative procedure based on the SNP effect solutions or based on posterior SNP variance did not perform as well as BSLMM. The first strategy estimates all SNP effects simultaneously and does not eliminate or reduce variance of SNP adjacent to potential causative SNP automatically. It proved, however, efficient in simulations studies (e.g., [16]), but was not compared to Bayesian approaches. In Calus et al. [12] and Su et al. [11], using posterior SNP variance to weight SNP in a GRM resulted in predictions that were almost as accurate as the ones form a Bayesian mixture model. In general, efficiency of these approaches is dependent on the number of large-effect variants (e.g., [10]). Conversely, methods relying on window of SNPs [21, 22] are computationally effective and could better capture multi-allelic variants or multiple co-localized SNP effects (including rare alleles not captured as single SNPs). Therefore, methods that operate on segments may be more efficient for traits with few causative SNP in the data or with multiple QTL per segment. They might also be more recommended at higher marker density since windows would be physically shorter (more precise mapping) but would contain equivalent number of markers. In addition, their computational efficiency would be a stronger asset. Similarly, methods partitioning the genetic variance in different groups of markers would be efficient with large number of variants (e.g., sequence information) and when functional information is available to classify these variants (e.g., [25]).

Compared to other studies (e.g., [6, 11, 23]), the application of Bayesian methods (or other SNP weighting strategies) resulted in relatively modest gains. One possible reason would be that the traits were highly polygenic (very few variants explaining more than 5% of the genetic variance and none capturing 10%). In addition, some large-effect variants might be poorly captured with our medium density marker panel and more records would be required to increase the power to identify them (or more accurate phenotypes such as daughter yield deviations from proven bulls). However, it has been previously observed that SNP selection increases reliability with small data sets but less so with large ones [43]. When the amount of information is small, only a fraction of all chromosome segments can be well estimated, and indirect selection of segments with larger effects by SNP selection is useful [44]. When all chromosome segments can be estimated well, preselection of segments is less useful. The data size in this study was relatively large whereas the number of independent chromosome segments was probably limited (the data came from a single breed with a low effective population size). Consequently, the impact of SNP selection was small. The impact will be even smaller in the future, when the amount of information in Belgian Blue cattle increases. In that situation, SNP effects would be estimated more accurately and large SNP effects would be less regressed towards the mean in GBLUP, making weighting less necessary. Conversely, the ssGBLUP approach resulted in higher gains in reliabilities indicating that optimal inclusion of ungenotyped animals was more important than optimal SNP weighting in our current setting. Nevertheless, individual SNP weighting strategies can also be incorporated in the ssGBLUP [13, 15, 16, 38]. Here, we observed that such a strategy could further improve reliability of ssGBLUP. An alternative strategy could be to apply the model presented by Fernando et al. [45] that combines genotyped and ungenotyped individuals in a Bayesian setting.

Although weighting strategies might increase prediction accuracies, their impact on long term evolution of genetic progress, maintenance of genetic variance and genetic diversity should also be considered. Some studies suggested that higher weights to frequent or large effects SNPs might lead to their rapid fixation and reduce long term genetic gain (e.g., [46]). In particular, in the case of the Belgian Blue cattle breed, we should be careful in selecting variants with deleterious effects at homozygous state.

## Conclusions

The AM-BLUP is an attractive method to automatically identify regions in the genome with large effects on complex traits and to estimate the proportion of genetic variance they capture. As a result, an optimally weighted GRM is automatically created and can be used in weighted GBLUP or ssGBLUP settings to improve genomic prediction accuracy. Although the approach proved very efficient on human data, it was less adapted to medium density genotyping arrays and high LD levels as typically observed in cattle. An alternative strategy, using Bayesian methods to identify group of SNPs with large effects and estimating their variance with a REML approach resulted in higher accuracies. Nevertheless, the computational efficiency of the AM-BLUP might be valuable at higher marker density, including with whole-genome sequencing data, or to estimate variance associated with different functional categories of SNPs.

## Materials and methods

### Data

A set of 10,192 cows genotyped with four different versions of the Illumina Bovine Low Density genotyping array [47] was used in the present study. We selected 8002 markers shared by the four low density arrays and also present on the first two versions of the Illumina Bovine 50 K array (v1 and v2). Only individuals with call rate above 90% were included in the analysis. Markers with a low call rate (< 0.95), deviating from Hardy-Weinberg proportions (*p* < 0.001) or with more than 10 incompatible parent/offspring pairs (e.g., opposite homozygous genotypes) were discarded. Imputation to a higher density using a reference panel of 1839 individuals genotyped for 35,207 SNPs common to the Illumina Bovine50K and Illumina BovineHD genotyping arrays was done using Beagle 3.3.2 [48]. The target and reference panels were phased with Beagle 3.3.2 prior to imputation with default settings. In addition to these 35,207 markers, we also included a set of 984 customer-specific probes passing the same filtering rules and present on the low density marker panels. These markers were selected for research projects (e.g., [35]), for genetic testing for recessive diseases and by the Walloon breeder organisation. These specific probes contained variants predicted to have potential deleterious effects such as loss-of-function variants. As a result, a total of 36,191 SNPs was available per individual.

The 10,192 selected cows were scored from 1993 to 2017 for 22 linear classification traits. From these, we selected 14 traits related to muscular development and conformation including length, chest-width, rib-shape, rump, tail-set, pelvis-length, buttock-muscling (side view), buttock-muscling (rear view), pelvis-width, shoulder-muscling, top-muscling and muscular development (available for 9830 to 9888 individuals) and also height and skin thickness (available for 8863 and 8823 cows, respectively). Trait deviations (TD) were obtained by correcting these records for fixed effects estimated in the official genetic evaluations.

## Methods

### Reference genomic prediction methods

We start by describing three reference methods for whole genome prediction, the genomic BLUP (**GBLUP**) that assumes that all SNP effects have the same variance, BayesR [7] and the Bayesian Sparse Linear Mixed Model (**BSLMM**) from Zhou and Stephens [8] that both allow some SNP to have large effects.

#### Genomic BLUP prediction model

The GBLUP model can be described as follows:

$$ \boldsymbol{y}=\mathbf{1}\mu +\boldsymbol{g}+\boldsymbol{e} $$

where ***y*** is a vector of the trait deviations (**TD**) for the cows, μ is the general mean effect, **g** is the vector of additive genetic random effects normally distributed, i.e., **g** ~ N(0,**G**${\sigma}_g^2$), **e** is a vector of residual effects normally distributed, i.e., **e** ~ N(0,**I**${\sigma}_e^2$), ${\sigma}_g^2$ and ${\sigma}_e^2$ are respectively the additive genetic and residual variances, **I** is an identity matrix and **G** is the GRM built as in VanRaden et al. [2].

$$ \mathbf{G}=\frac{\mathbf{XX}^{\prime }}{\sum 2{f}_j\left(1-{f}_j\right)} $$

Here, **X** is a matrix of dimension (*n* x *m*) of centered SNP genotypes, where *n* is number of animals and *m* is the number of SNPs, with values *x*_*ij*_ equal to:

$$ {x}_{ij}={s}_{ij}-2{f}_j $$

where *s*_*ij*_ is the number of copies of the reference allele carried by individual *i* at marker *j*. The reference allele frequencies *f*_*j*_ are used to centre these allele dosages. Under this model, SNP effects have equal variance.

#### Standardized GBLUP prediction model

Alternatively, a standardized GRM, **G**^**S**^, can be built with rules proposed by Amin et al. [5] or Astle and Balding [49].

$$ {\mathbf{G}}^{\mathbf{S}}=\frac{1}{m}\mathbf{WD}{\mathbf{W}}^{\mathrm{T}} $$

Where **W** is a matrix of dimension (*n* x *m*) of normalized SNP genotypes with values w_*ij*_ equal to:

$$ {w}_{ij}=\frac{\left({s}_{ij}-2{f}_j\right)}{\sqrt{2{f}_j\left(1-{f}_j\right)}} $$

The division by $\sqrt{2{f}_j\left(1-{f}_j\right)}$ is performed to obtain unit variance. In this formulation, all SNPs have equal expected contribution to the genetic variance and SNPs with low MAF have larger effects; SNPs are thus weighted according the inverse of their expected variance [2] (higher weights for low MAF). Bouwman et al. [50] showed that this weighting affected solutions of SNP effects, in particular those with a low MAF (0 < . < 0.01). Note, that in the present study, we conserved only SNP with a MAF > 0.01 (and most SNP had a MAF > 0.05). The matrix **D**, a diagonal matrix with diagonal elements *d*_*jj*_ equal to 1 when all SNP have equal contribution to the genetic variance, allows the introduction of different weights (see below).

We will refer to the standardized GBLUP when using **G**^S^. It allows better comparisons with the other tested genomic prediction methods, and with the adaptive MultiBLUP approach in particular, because these rely also on standardized genotypes. In the present study, we used BLUPF90 [51] to apply both GBLUP approaches.

#### Bayesian sparse linear mixed model

The BSLMM assumes that all SNPs have at least a relatively small effects but also that a few SNP can have a large effect [8]. The model can be presented as follows:

$$ \boldsymbol{y}=\mathbf{1}\mu +\mathbf{X}\boldsymbol{\upbeta } +\boldsymbol{e} $$

Where **β** is the vector of SNP effects. The individual SNP effects β_i_ are sampled from a mixture of two normal distributions, ${\beta}_i\sim \pi N\left(0,{\sigma}_a^2+{\sigma}_b^2\right)+\left(1-\pi \right)N\left(0,{\sigma}_b^2\right)$ where ${\sigma}_b^2$ is the variance of small effects, ${\sigma}_a^2$ is the additional variance associated to large effects and π is the proportion of SNPs with large effects. The model is implemented in a Bayesian framework and MCMC simulations are realized to obtain approximate samples from the posterior distribution. In particular, we obtain a posterior inclusion probability (**PIP**) for each SNP that indicates the proportion of samples in which that SNP has a large effect. That proportion can be used for fine-mapping approaches but also for SNP prioritization. Here, we used the standardized SNP genotypes matrix (i.e., **W)** when applying BSLMM.

#### BayesR

In this model, SNP effects are sampled from a mixture of four normal distributions with mean zero and variances equal to 0, 0.0001 ${\sigma}_g^2$, 0.001 ${\sigma}_g^2$ and 0.01 ${\sigma}_g^2$:

$$ {\beta}_i\sim {\pi}_1N\left(0,0{\sigma}_g^2\right)+{\pi}_2N\left(0,0.0001{\sigma}_g^2\right)+{\pi}_3N\left(0,0.001{\sigma}_g^2\right)+{\pi}_4N\left(0,0.01{\sigma}_g^2\right) $$

SNP effects were estimated using MCMC simulations and the software implemented in Moser et al. [9].

### Adaptive MultiBLUP model (AM-BLUP)

#### Description of the original approach

The adaptive MultiBLUP approach is described in Speed and Balding [22] and implemented in the LDAK software. It starts with a genome-scan where associations are tested for windows of SNPs called chunks. These chunks have a constant physical size (e.g., 500 kb, 1 Mb) and the overlap between successive chunks is 50%. For each window, a GRM is estimated using the SNPs in the window and the rules described above. A likelihood ratio test (**LRT**) is then performed to test whether the variance associated with the region is significantly different from 0 (the default threshold *thr1* for genome-wide significance is set to 10^− 5^). In the second step, regions are formed around significant chunks by merging them with all adjacent chunks with a *p*-value < *thr2* (where *thr2* is a second threshold set by default to 0.01). A GRM is estimated for each of these K merged regions and the background kinship matrix is estimated with SNPs outside the selected regions. Variances associated to each of the K + 1 GRMs, ${\sigma}_k^2$, are then estimated simultaneously with LDAK by a REML procedure (where *k* is the index for the region (or its GRM) and 0 is used for the background kinship). Finally, genomic predictions are obtained by using the K + 1 GRMs and the estimated variance components. This is equivalent to apply a GBLUP model with a single GRM where each SNP is weighted by its own variance (e.g., the variance associated to its GRM divided by the number of SNPs in the corresponding region):

$$ {\mathbf{G}}^{AM}=\mathbf{W}{\boldsymbol{D}}^{\mathrm{AM}}\boldsymbol{W}^{\prime } $$

Where **G**^AM^ is the Adaptive MultiBLUP GRM and **D**^AM^ is a diagonal matrix with the corresponding weights, ${d}_i^{AM}$, equal to:

$$ {d}_i^{AM}=\frac{\sigma_k^2}{m_k}{\left(\frac{\sigma_T^2}{m}\right)}^{-1} $$

Where m_k_ is the number of SNPs in region k encompassing SNP i and ${\sigma}_T^2$ is the total genetic variance. Division by the mean SNP variance ensures that the trace of **D**^AM^ is maintained constant as for instance in Wang et al. [13]:

$$ tr\left(\boldsymbol{D}\right)=\sum \limits_{k=0}^K{m}_k\frac{\sigma_k^2}{m_k}{\left(\frac{\sigma_T^2}{m}\right)}^{-1}=\frac{m}{\sigma_T^2}\sum \limits_{k=0}^K{\sigma}_k^2=m $$

#### Using permutations and bootstrapping techniques to define significance thresholds and confidence intervals

Thresholds *thr1* and *thr2* are used to declare regions significant and define their confidence interval (**CI**). To optimize these thresholds, we generated the empirical distribution of the test statistics under H0 through 1000 random permutations of the phenotypes [52], without changing individual genotypes. For each permutation, a genome scan was performed as described above and the window with the lowest *p-value* was selected. The empirical threshold at α = 0.05 was set at the 5% quantile of this distribution of 1000 best *p*-values. The CI was subsequently determined with a bootstrapping strategy [53]. For this, we started by selecting all windows with a *p*-value below 0.01 surrounding the most significant position (as in the original procedure described above). In addition, all significant regions less than 10 Mb apart were merged. For each significant region, 1000 bootstrap samples were obtained by randomly selecting individuals with replacement. For each sample, the most significant window was flagged. The 95% CI was then defined as the smallest set of windows being flagged 950 times or more (95%). These windows were then selected to build the GRM. Hereafter, we used BAM-BLUP as abbreviation (for Bootstrap-Adaptive-MultiBLUP) to refer to this method.

#### Alternative strategy for region selection

We also tested whether the BSLMM could be used to identify regions of interest. To that end, we used the posterior inclusion probabilities (PIP) to perform SNP selection. Since several SNPs in high LD might capture the effect of the same variant, we decided to work with 25 SNPs windows. For each window, we computed the region PIP as the sum of 25 individual PIP (one per SNP). Following Barbieri and Berger [54], regions with a PIP above 0.5 were selected (since SNPs from these regions are included in the model in the majority of iterations). Subsequently, we conserved only the SNPs that most contributed to the window PIP (excluding SNP with low PIP). To do that we ranked the SNPs from the window according to their PIP, and conserved the smallest set of SNPs that contributed to 95% of the total PIP. Then, we used LDAK to estimate the variance associated with each selected window and these variances were then used to build a weighted GRM as described above. This method will be referred to as WPIP-GBLUP (Window Posterior Inclusion Probability GBLUP).

### Other strategies to weight SNPs in the GBLUP approach

Other strategies to compute a GRM using weights for SNPs have been previously proposed. Wang et al. [13] developed an iterative scheme based on estimated SNP effects obtained by linear transformation of genomic breeding values (e.g., [55]) and implemented in the postGSf90 tool from the blupf90 package [39]. We performed 10 iterations of SNP weighting using non-linear methods [2] and limited the increase in variance to a factor of 2.6 as recommended by Fragomeni et al. [56]. We will use IW-GBLUP (iterative weighted GBLUP) as abbreviation for this method. Legarra et al. [17] or Su et al. [11] used rather the posterior variance obtained from Bayesian methods modelling SNP effects as a mixture of different distributions. Here we estimated individual SNP effect variances from the BayesR MCMC simulations (BRPV-GBLUP for BayesR Posterior Variance GBLUP). Individual SNP variances were stored at each iteration. These were obtained as a function of the sampled values for ${\sigma}_g^2$ and the sampled distribution for the SNP effect at that iteration (e.g., a SNP in the second distribution had a variance equal to 0.0001 ${\sigma}_g^2$). The posterior SNP variance was then computed as the average variance across all iterations. Finally, the weights were re-scaled so that the trace of **D** was equal to *m*.

### Extension to single step GBLUP approaches

Accuracy of GBLUP approaches using the different weighted GRM were compared. All the weighted GRM approaches were also applied in the single step GBLUP (ssGBLUP) method, which combines information from genotyped and non-genotyped individuals [3, 4]. The ssGBLUP relied on the official evaluation model, with raw instead of pre-corrected phenotypes. Pedigree files contained from 618,962 to 653,008 individuals and the total number of available records ranged from 456,746 to 494,541 according to the trait. The BLUPF90 package was used to run the ssGBLUP models.

### Association study

Regions or SNPs identified by the adaptative MultiBLUP and BSLMM approaches were compared to results from a genome-wide association study (GWAS) performed using GLASCOW [57] that uses a score test for each SNP in a linear mixed model (**LMM**) framework. We set the significant threshold at 1.39e-6 after application of a Bonferroni correction for 36,191 independent tests. Peaks were declared independent when two significant SNPs were more than 10 Mb apart.

### Evaluation of different genomic prediction models

To compare the prediction accuracy from different models, the data was divided in training and validation sets. To that end, we removed all performances recorded after August 23th of 2018 which corresponded to individuals born more recently. As a result, the training set was composed of 7316 to 8532 individuals (according to the trait), whereas the validation group contained from 1356 to 1507 individuals (14 to 17% of the total individuals with both phenotypes and genotypes). The prediction accuracy was estimated as the correlation between the trait deviation and the genomic estimated breeding values (predictive abilities). The reliability was obtained as the square of the accuracy divided by the heritability:

$$ \mathrm{REL}=\frac{\mathrm{cor}{\left(\mathbf{y},\hat{\mathbf{g}}\right)}^2}{{\mathrm{h}}^2} $$

Methods were also compared in terms of mean-square errors (MSE) between predicted and observed TD, eventually standardized by the variance of the TD. Bias of genomic predictions (i.e., “scale” bias or inflation) were measured using the coefficients of the regression of the TD on the genomic predictions.

## Supplementary information

**Additional file 1.**

**Additional file 2.**

**Additional file 3.**

## Data Availability

The data that support the findings of this study are available from Elevéo asbl and Inovéo (Awé Group, Belgium) but restrictions apply to the availability of these data, which were used under license for the current study, and so are not publicly available. Data are however available from the authors upon reasonable request and with permission of Elevéo asbl and Inovéo (Awé Group, Belgium).

## Abbreviations

GBLUP: Genomic best linear unbiased prediction
GRM: Genomic relationship matrices
GEBV: Genomic breeding values
BBB: Belgian Blue beef
REML: Restricted maximum likelihood
LD: Linkage disequilibrium
CI: Confidence interval
LRT: Likelihood ratio test
AM-BLUP: Adaptive MultiBLUP
BAM-BLUP: Bootstrap Adaptive MultiBLUP
BSLMM: Bayesian sparse linear mixed model
WPIP-GBLUP: Window Posterior Inclusion Probability GBLUP
IW-GBLUP: Iterative weighted GBLUP
BRPV-GBLUP: BayesR Posterior Variance GBLUP
ssGBLUP: Single step GBLUP
REL: Reliability
*NCAPG-LCORL*: Ligand dependent nuclear receptor corepressor like / non-SMC condensin I complex subunit G
CMD1: Congenital muscular dystonia 1
CCND2: Cyclin D2
PLEKHA1: Pleckstrin homology domain containing A1
MRC2: Mannose receptor C type 2
RNF11: Ring finger protein 11
WWP1: WW domain containing E3 ubiquitin protein ligase 1
ATP2A1: ATPase sarcoplasmic/endoplasmic reticulum Ca2+ transporting 1
KITLG: KIT ligand
GWAS: Genome-wide association study
LMM: Linear mixed model
MSE: Mean-square errors
PIP: Posterior inclusion probability
TD: Trait deviation
