# Supplementary material for: Performances of Adaptive MultiBLUP, Bayesian regressions, and weighted-GBLUP approaches for genomic predictions in Belgian Blue beef cattle
Source: BMC Genomics. 2020 Aug 6;21:545. doi: 10.1186/s12864-020-06921-3 (PMC7430838; doi:10.1186/s12864-020-06921-3)
Supplement: Supplementary file 2 — Additional file 2. [file 12864_2020_6921_MOESM2_ESM.pdf]

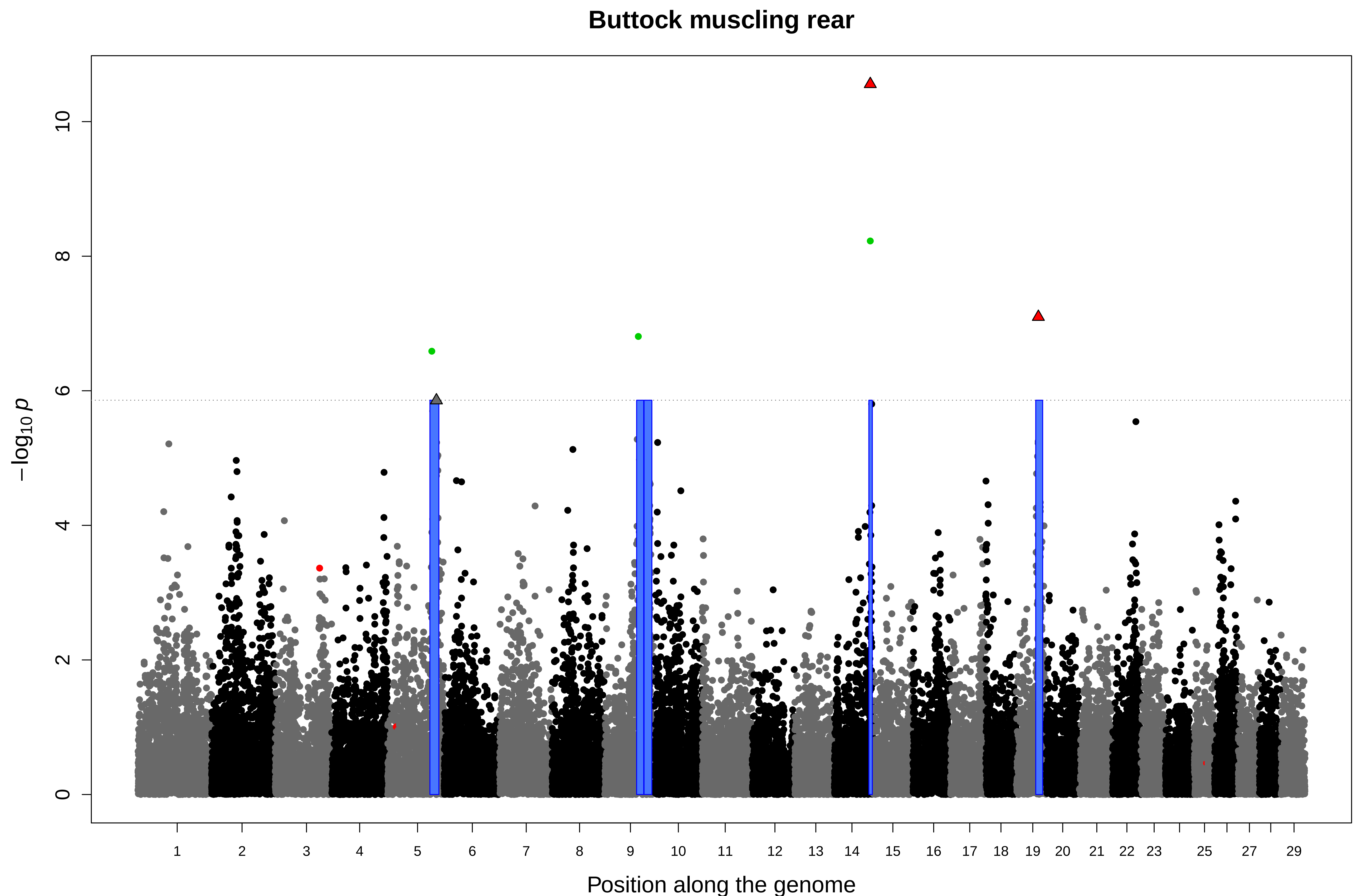

Buttock muscling rear
−log10 p
0
2
4
6
8
10
1
2
3
4
5
6
7
8
9
10
11
12
13
14
15
16
17
18
19
20
21
22
23
25
27
29
Position along the genome

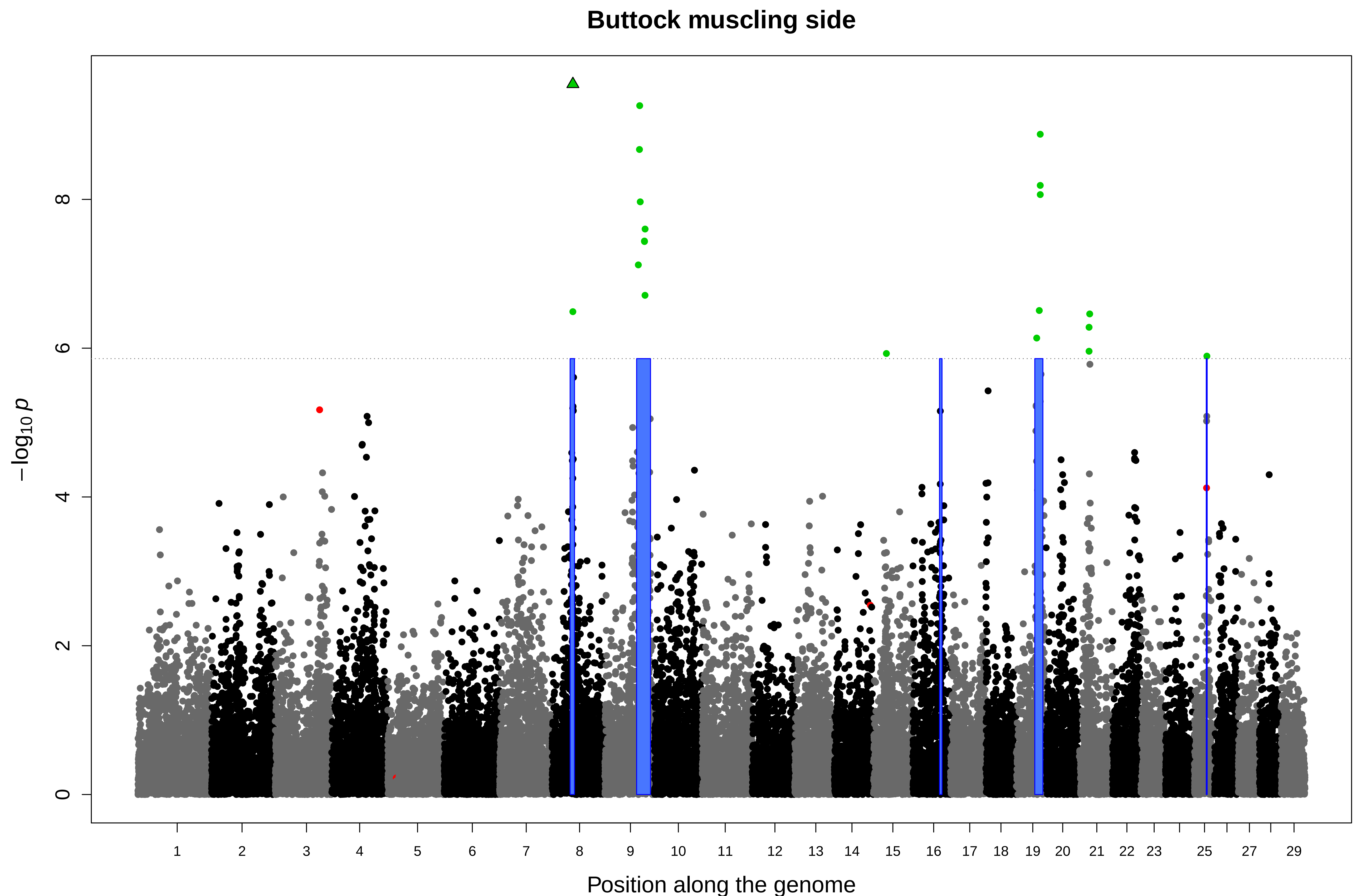
Buttock muscling side
−log10 p
0
2
4
6
8
Position along the genome
1
2
3
4
5
6
7
8
9
10
11
12
13
14
15
16
17
18
19
20
21
22
23
25
27
29

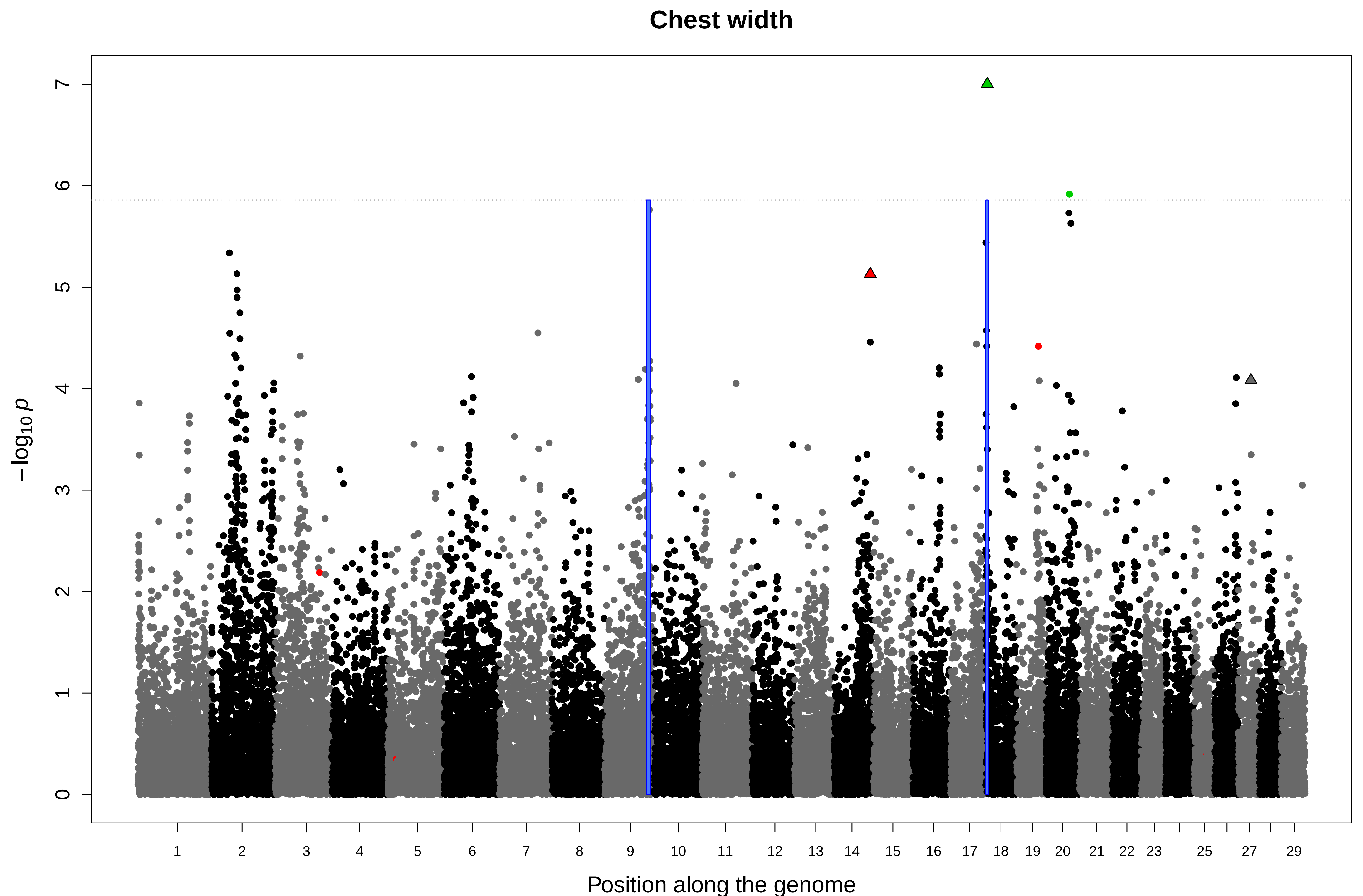

Chest width
7
6
5
4
3
2
1
0
$-\log_{10} p$
1
2
3
4
5
6
7
8
9
10
11
12
13
14
15
16
17
18
19
20
21
22
23
25
27
29
Position along the genome

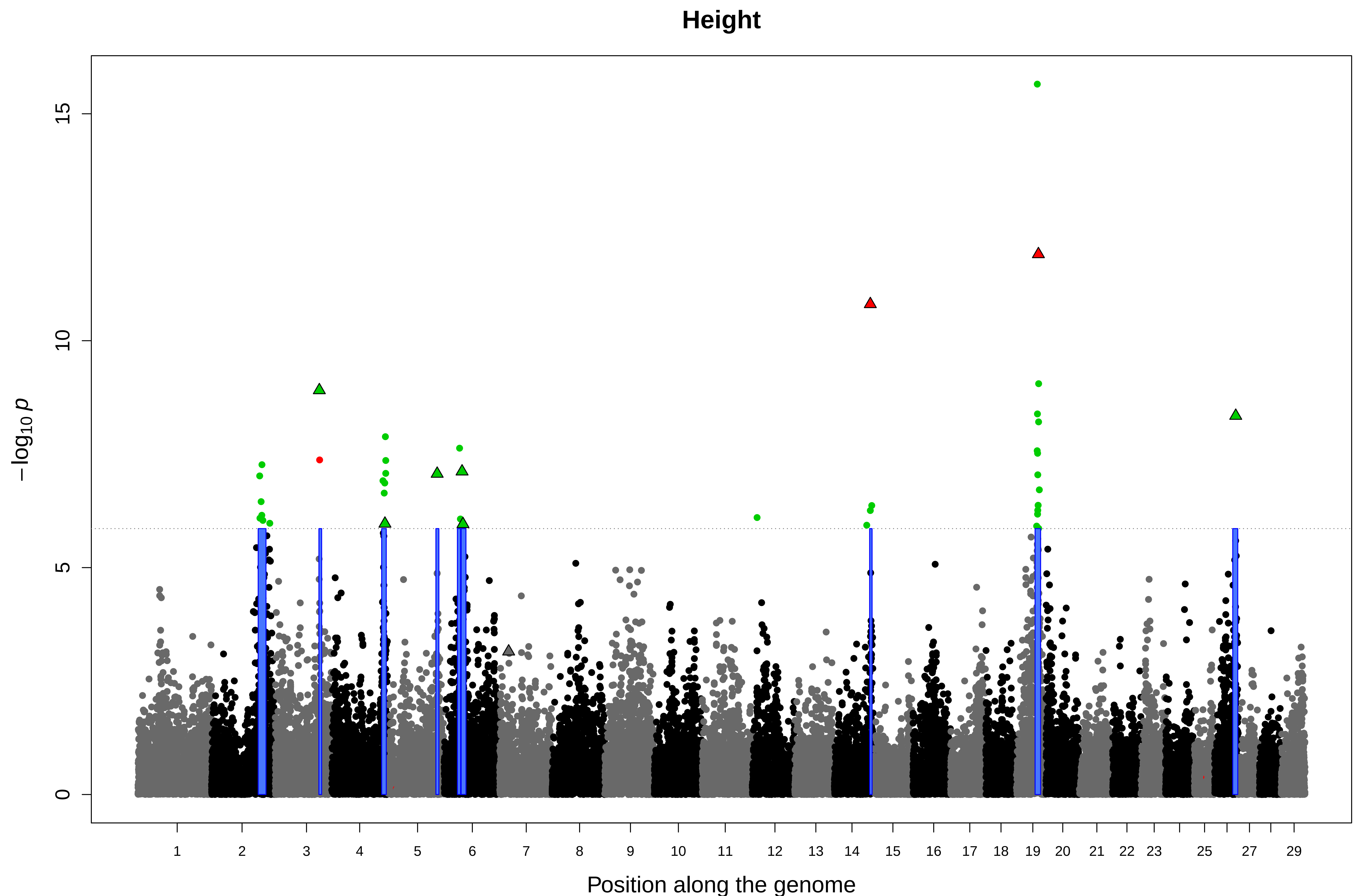

Height
−log10 p
0
5
10
15
1
2
3
4
5
6
7
8
9
10
11
12
13
14
15
16
17
18
19
20
21
22
23
25
27
29
Position along the genome

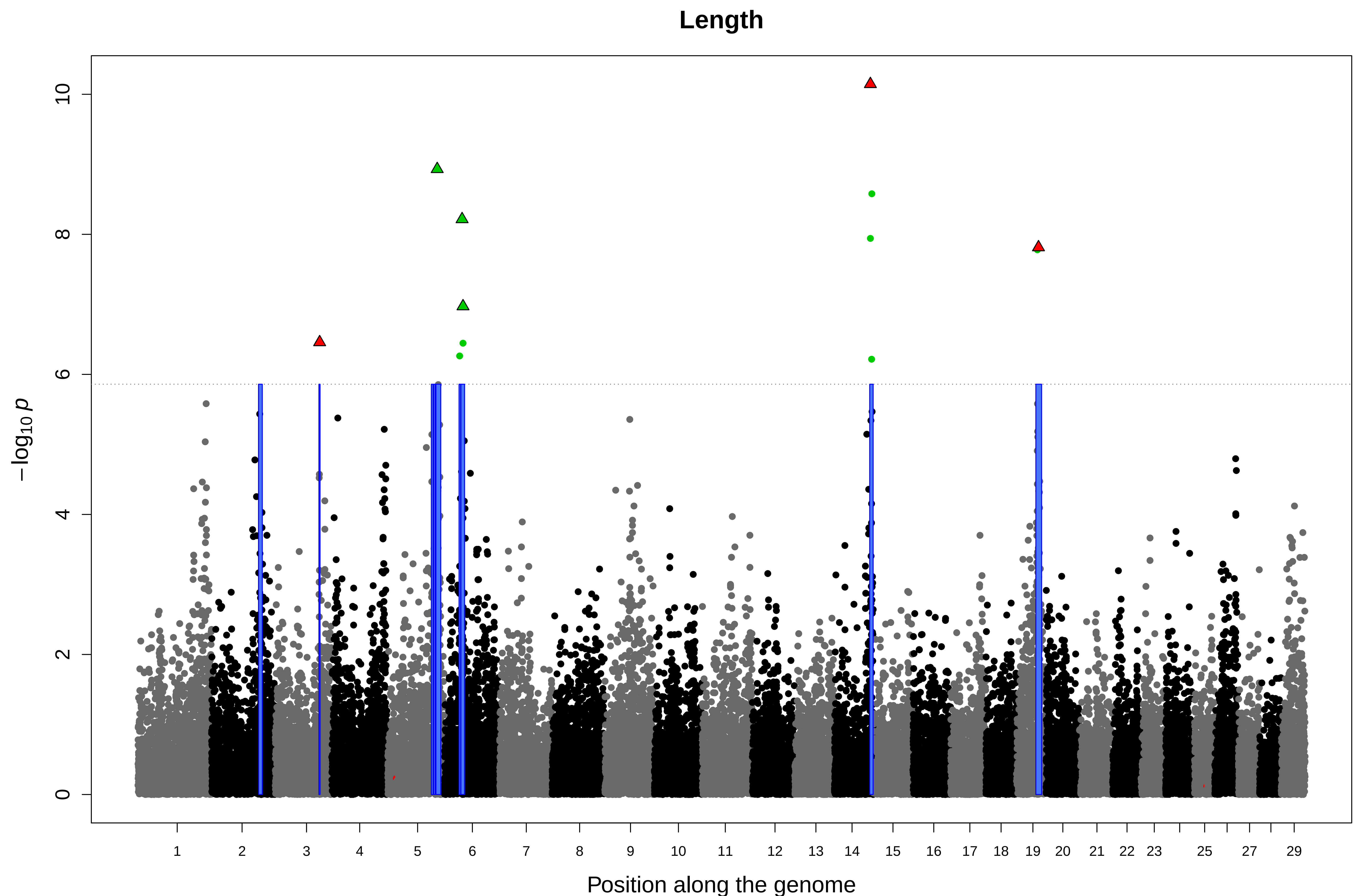

Length
10
8
6
4
2
0
−log10 p
1
2
3
4
5
6
7
8
9
10
11
12
13
14
15
16
17
18
19
20
21
22
23
25
27
29
Position along the genome

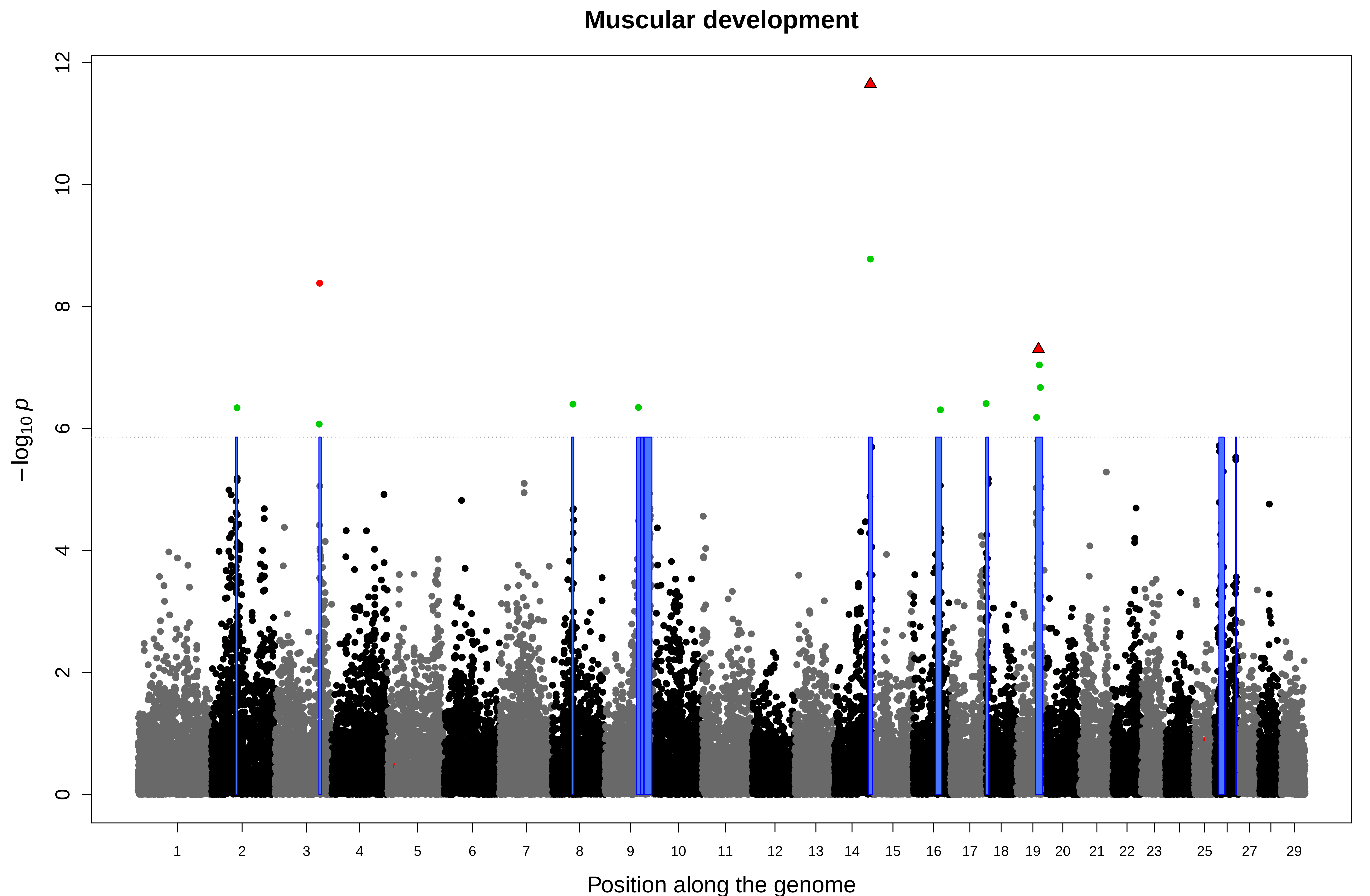
Muscular development
$-\log_{10} p$
0
2
4
6
8
10
12
1
2
3
4
5
6
7
8
9
10
11
12
13
14
15
16
17
18
19
20
21
22
23
25
27
29
Position along the genome

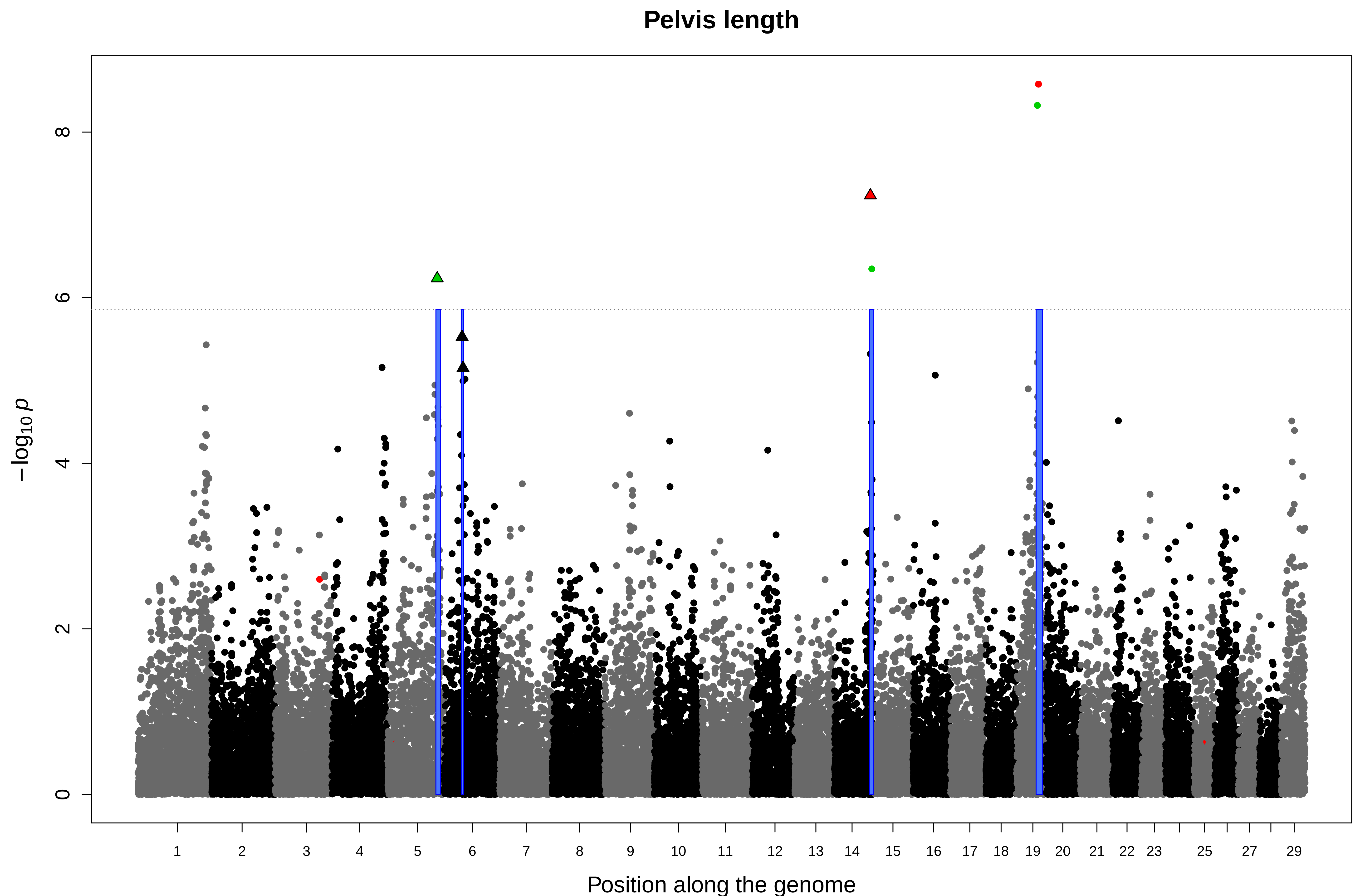

Pelvis length
−log10 p
0
2
4
6
8
1
2
3
4
5
6
7
8
9
10
11
12
13
14
15
16
17
18
19
20
21
22
23
25
27
29
Position along the genome

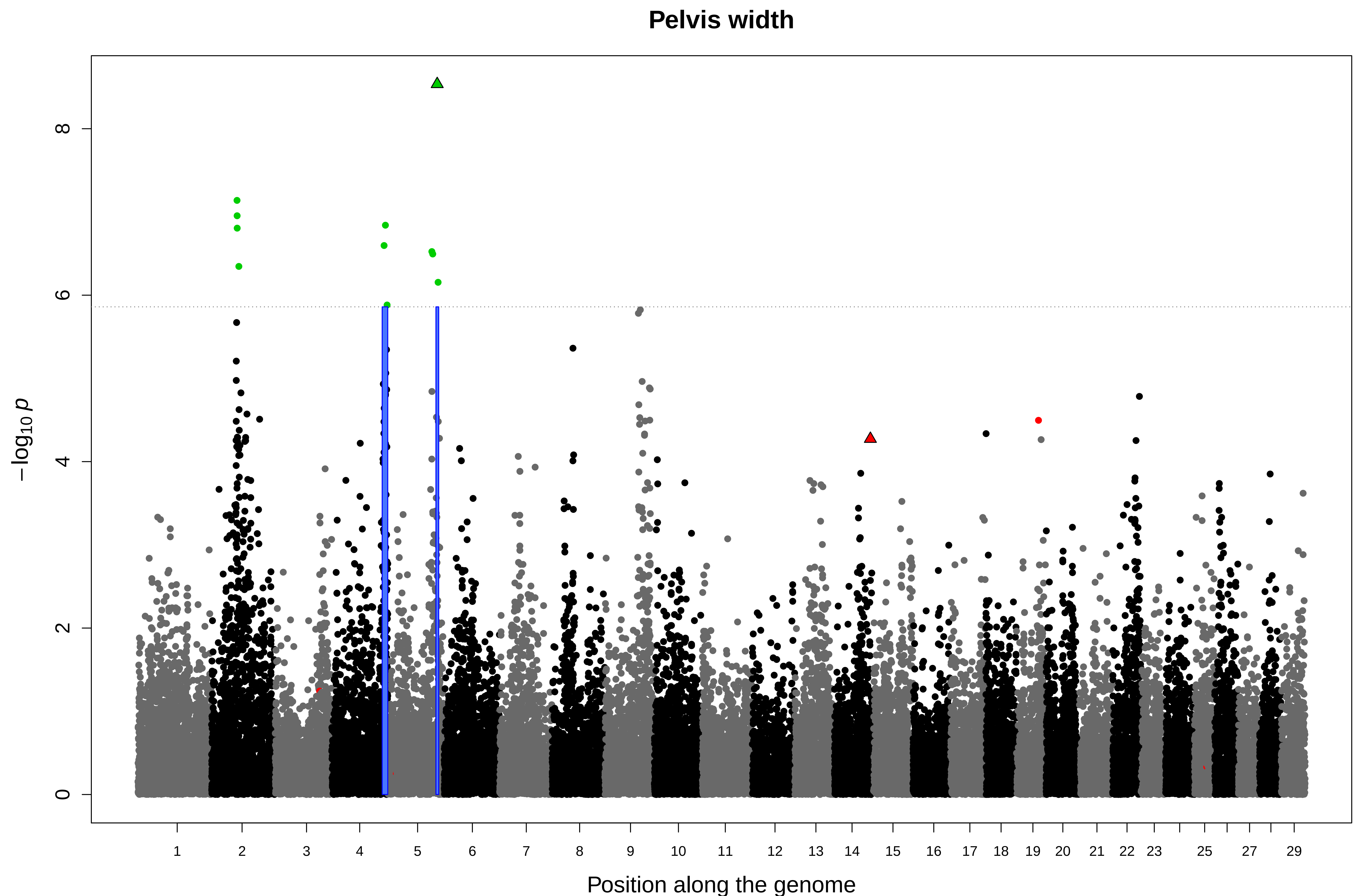

Pelvis width
$-\log_{10} p$
0
2
4
6
8
Position along the genome
1
2
3
4
5
6
7
8
9
10
11
12
13
14
15
16
17
18
19
20
21
22
23
25
27
29

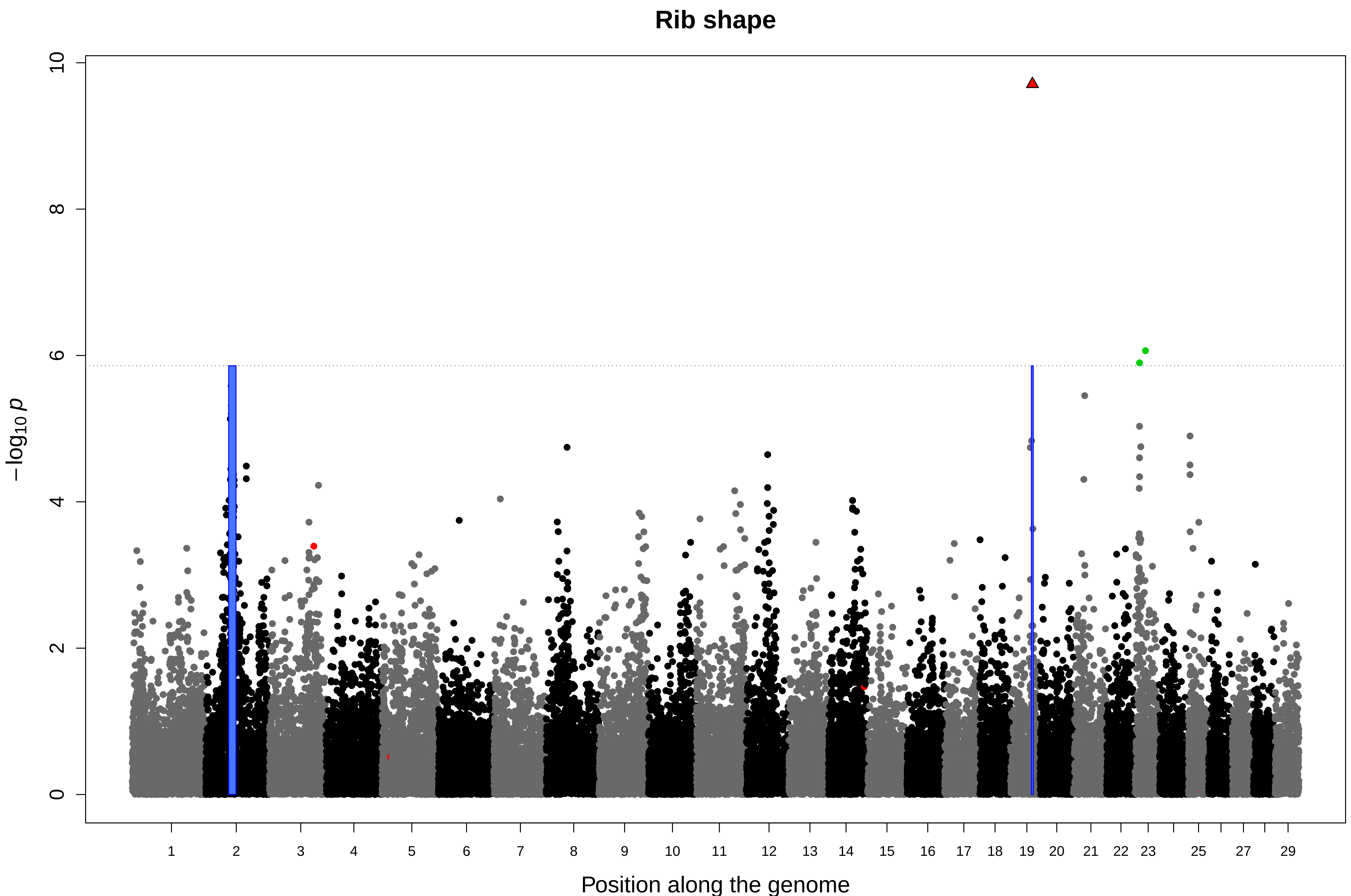

Rib shape
−log10 p
0
2
4
6
8
10
Position along the genome
1
2
3
4
5
6
7
8
9
10
11
12
13
14
15
16
17
18
19
20
21
22
23
25
27
29

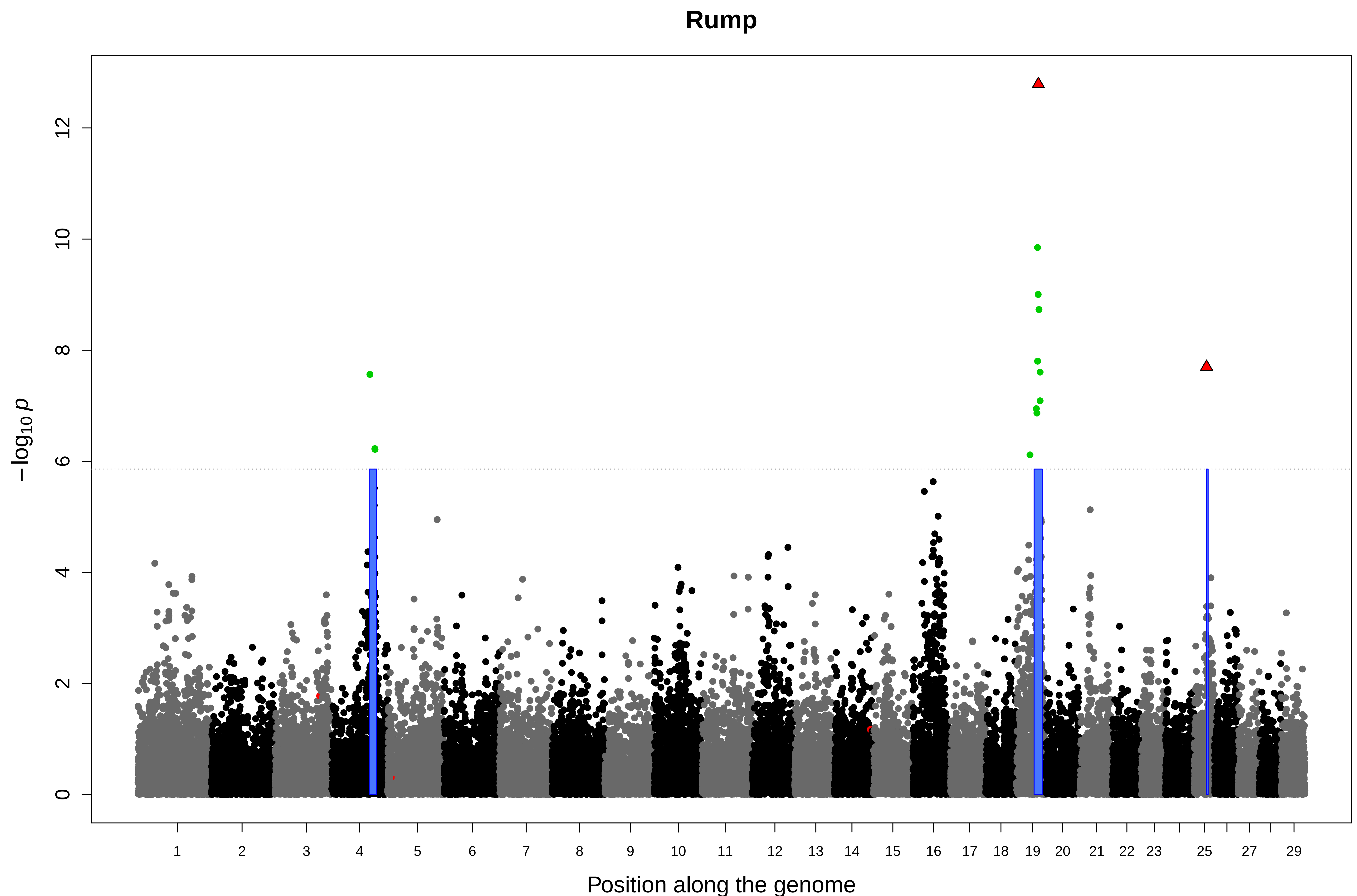

Rump
−log10 p
0
2
4
6
8
10
12
1
2
3
4
5
6
7
8
9
10
11
12
13
14
15
16
17
18
19
20
21
22
23
25
27
29
Position along the genome

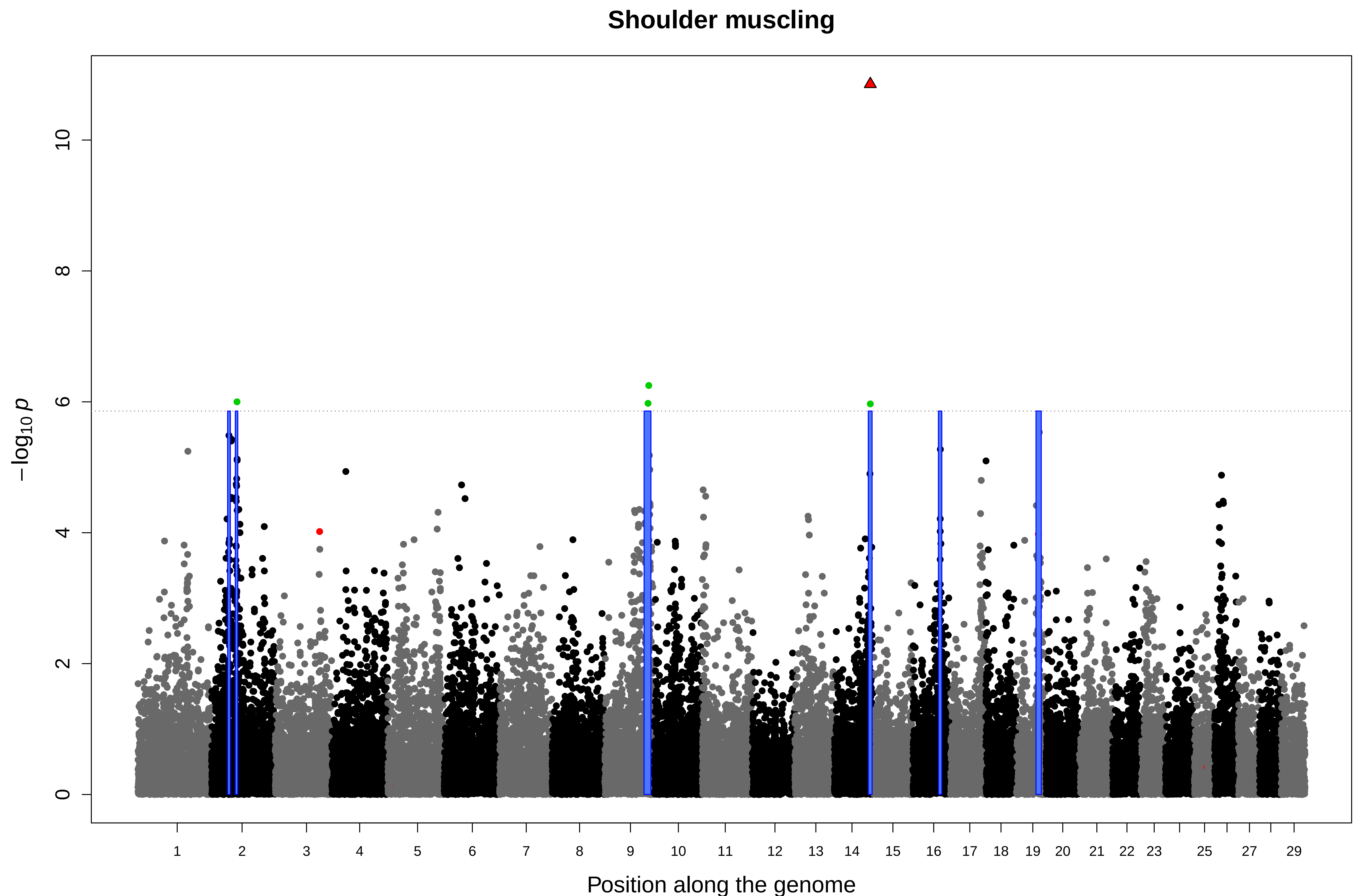

Shoulder muscling
−log10 p
0
2
4
6
8
10
1
2
3
4
5
6
7
8
9
10
11
12
13
14
15
16
17
18
19
20
21
22
23
25
27
29
Position along the genome

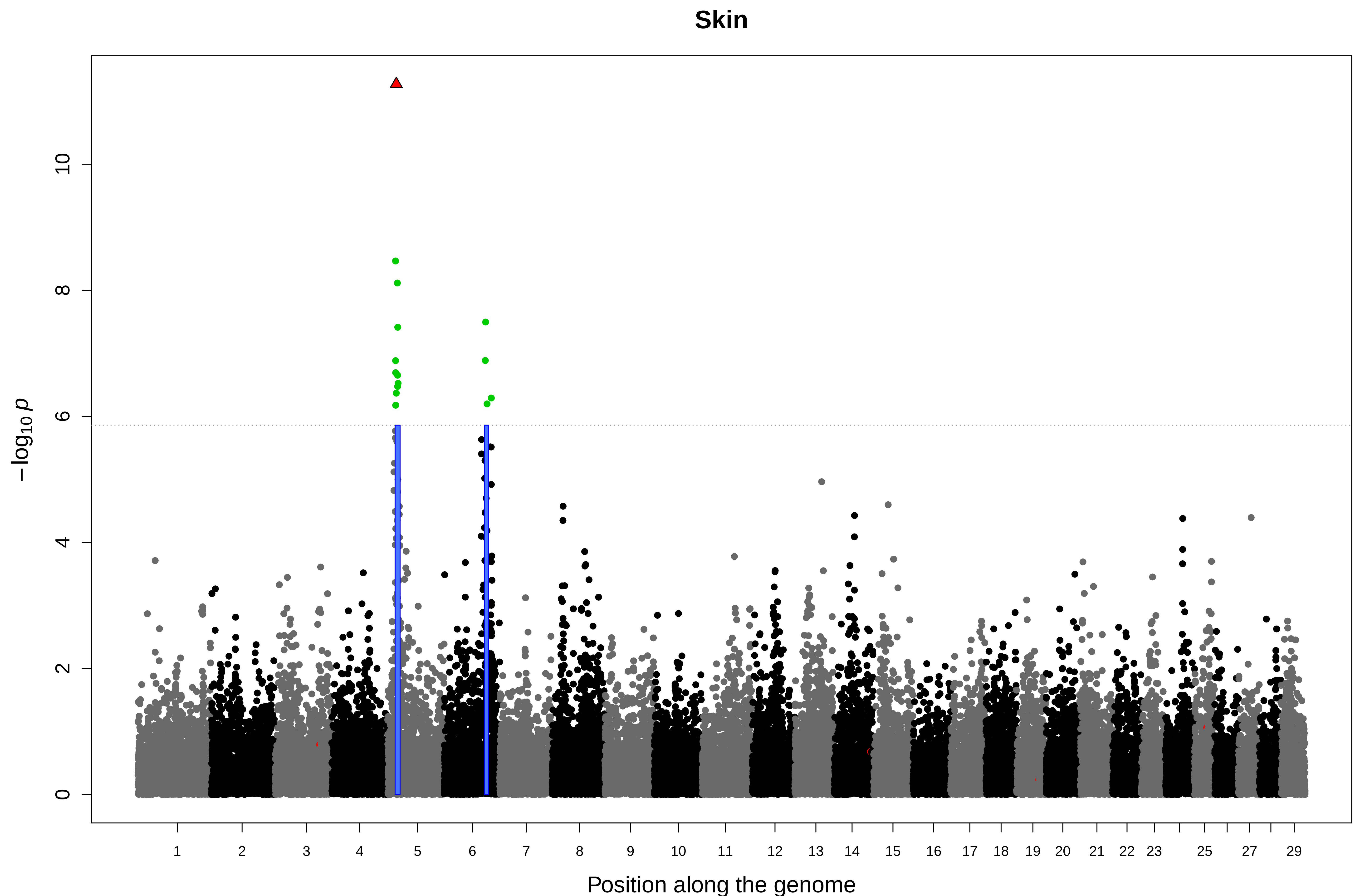

Skin
−log10 p
0
2
4
6
8
10
1
2
3
4
5
6
7
8
9
10
11
12
13
14
15
16
17
18
19
20
21
22
23
25
27
29
Position along the genome

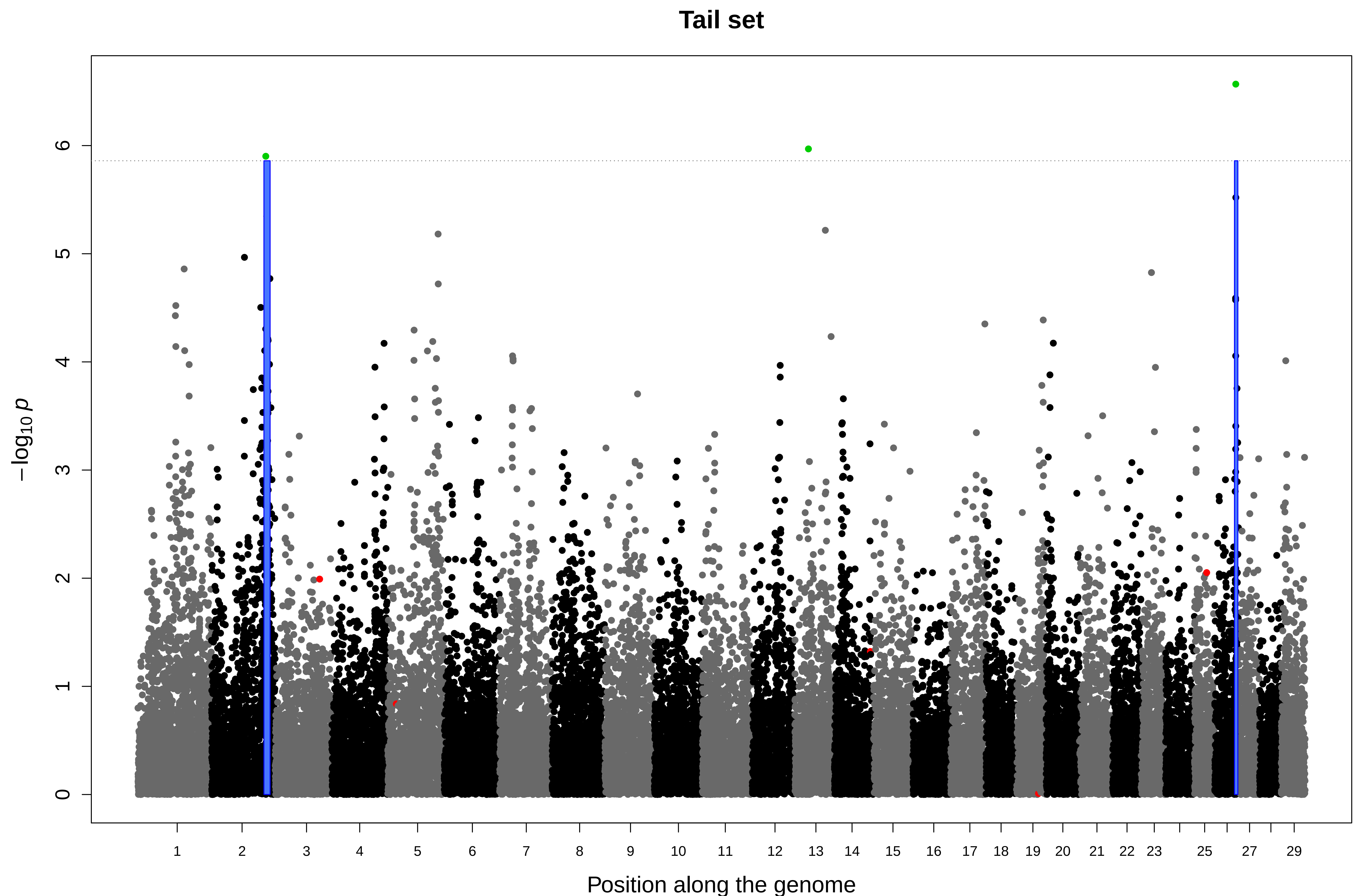
Tail set
−log10 p
0
1
2
3
4
5
6
1
2
3
4
5
6
7
8
9
10
11
12
13
14
15
16
17
18
19
20
21
22
23
25
27
29
Position along the genome

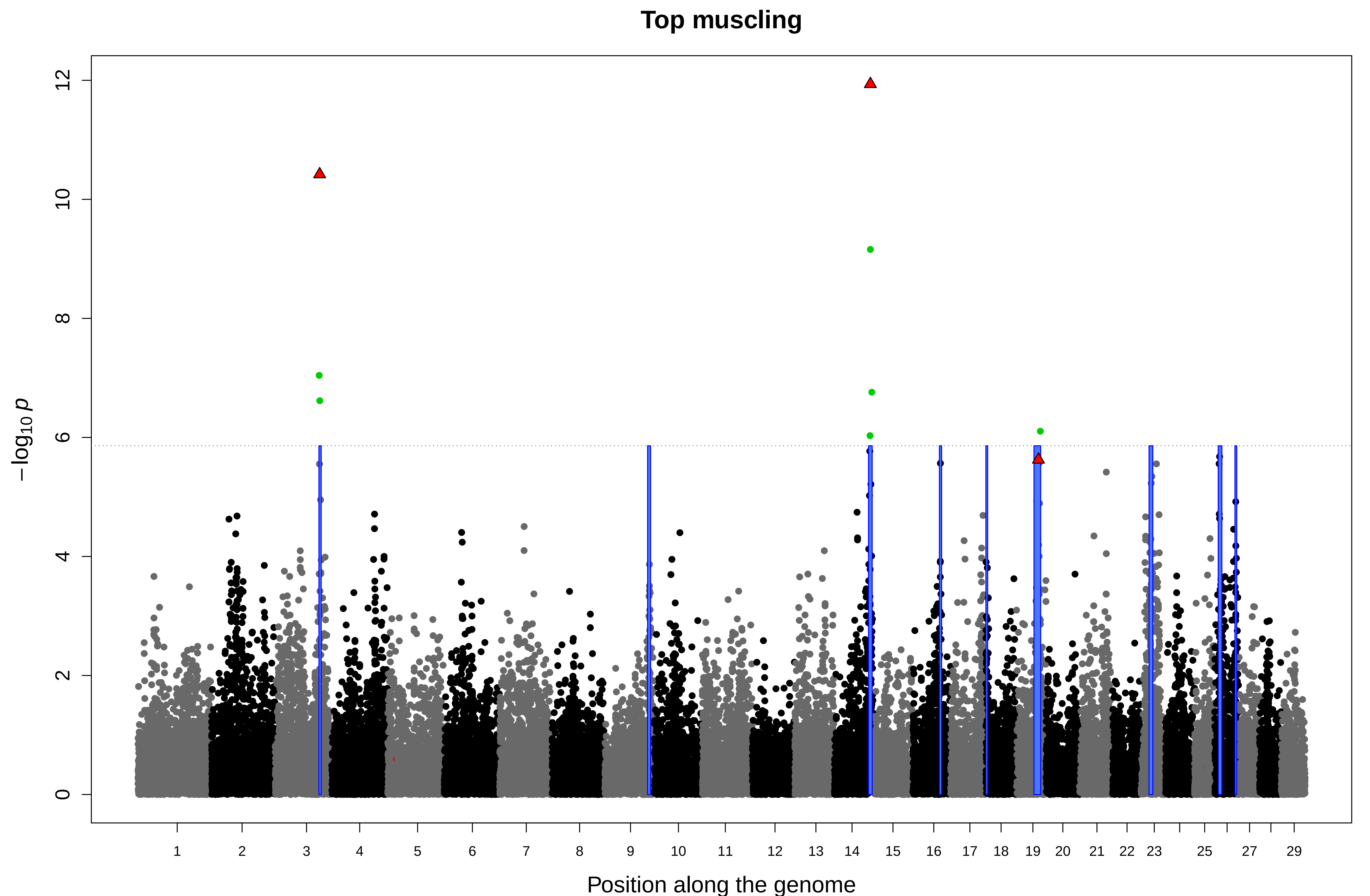

Top muscling
−log10 p
0
2
4
6
8
10
12
Position along the genome
1
2
3
4
5
6
7
8
9
10
11
12
13
14
15
16
17
18
19
20
21
22
23
25
27
29
