# Supplementary material for: Performances of Adaptive MultiBLUP, Bayesian regressions, and weighted-GBLUP approaches for genomic predictions in Belgian Blue beef cattle
Source: BMC Genomics. 2020 Aug 6;21:545. doi: 10.1186/s12864-020-06921-3 (PMC7430838; doi:10.1186/s12864-020-06921-3)
Supplement: Supplementary file 3 — Additional file 3. [file 12864_2020_6921_MOESM3_ESM.pdf]

Buttock muscling rear

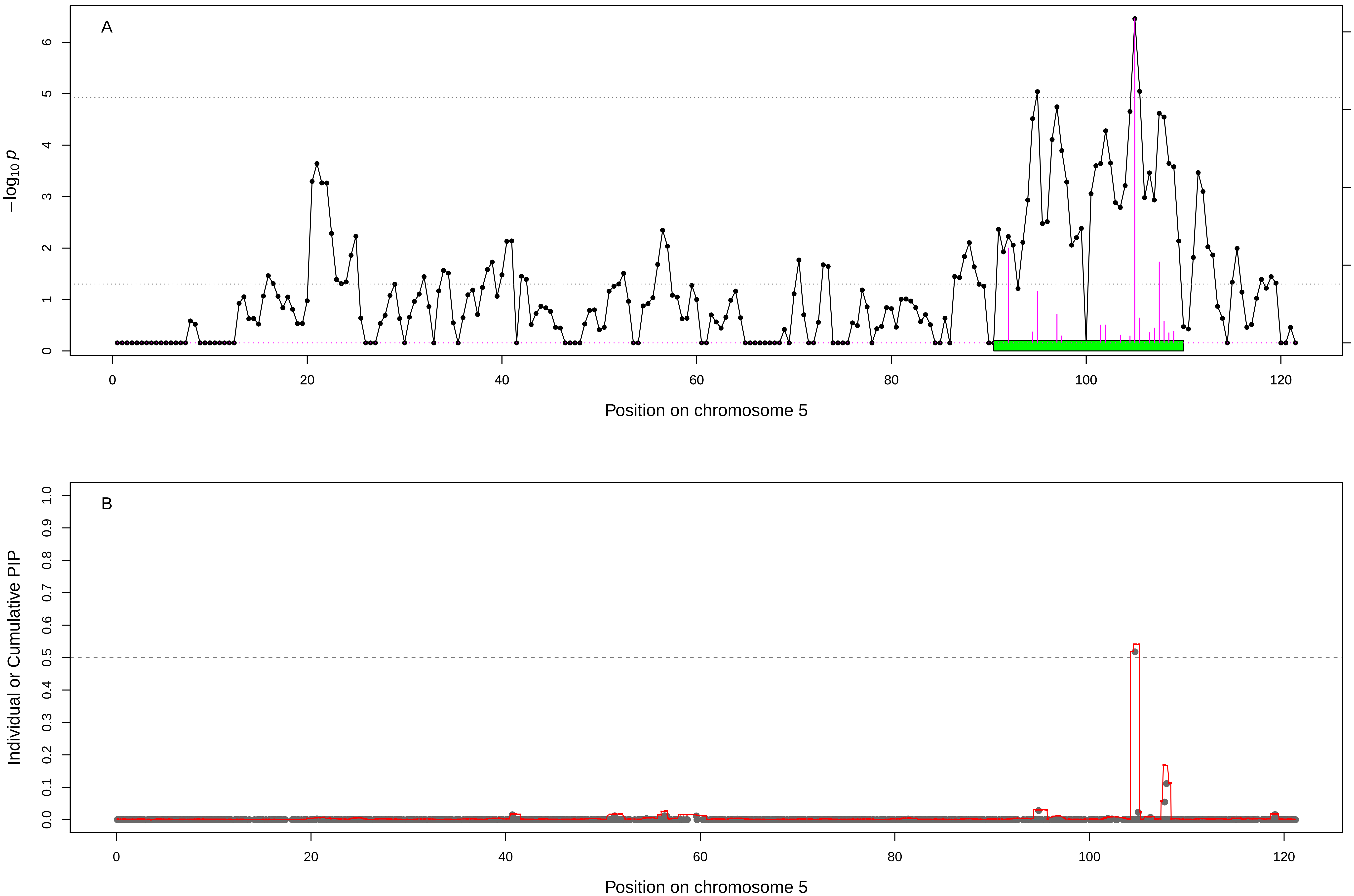

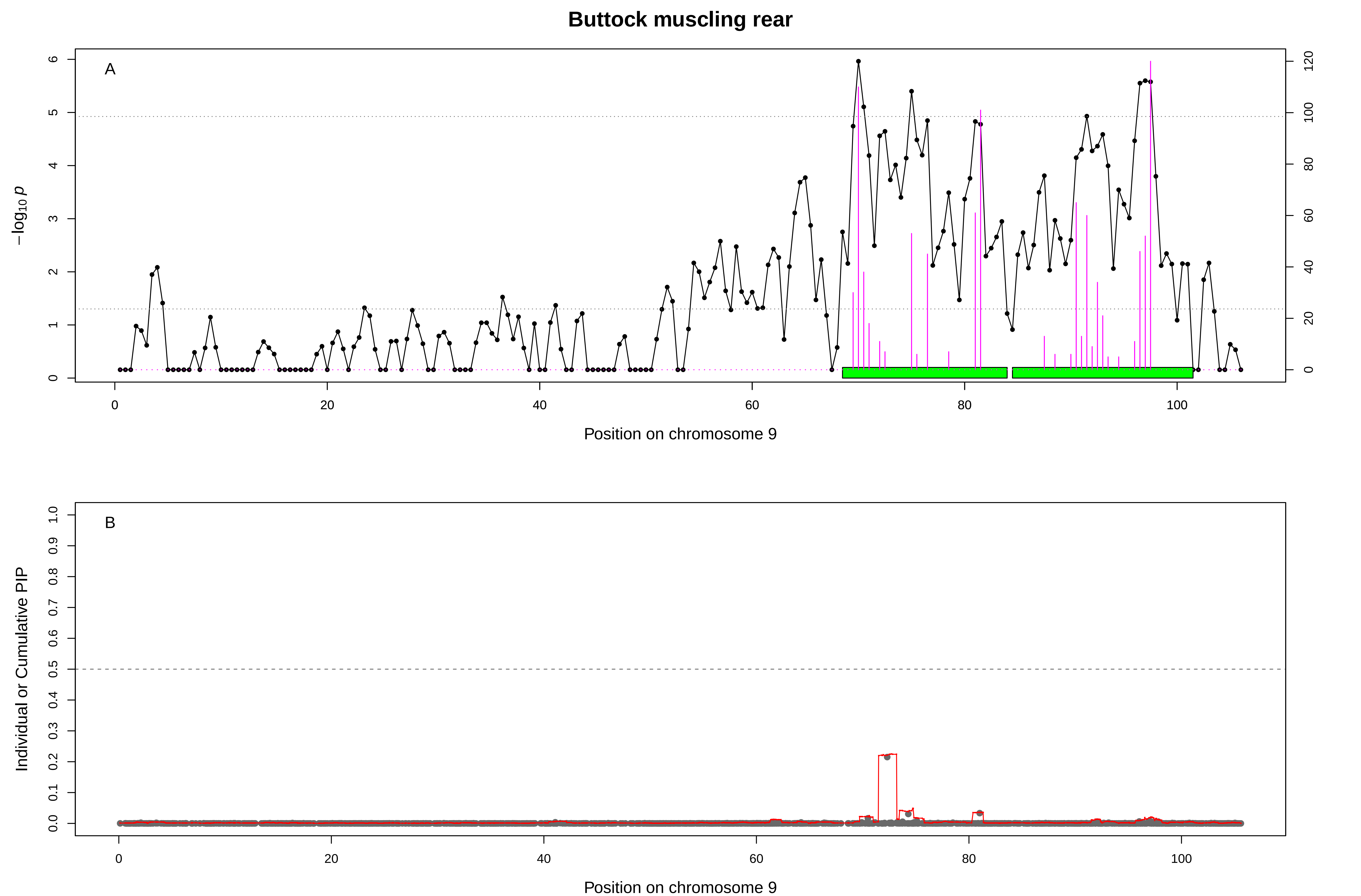

Buttock muscling rear
A
−log10 p
Position on chromosome 9
B
Individual or Cumulative PIP
Position on chromosome 9

Buttock muscling rear

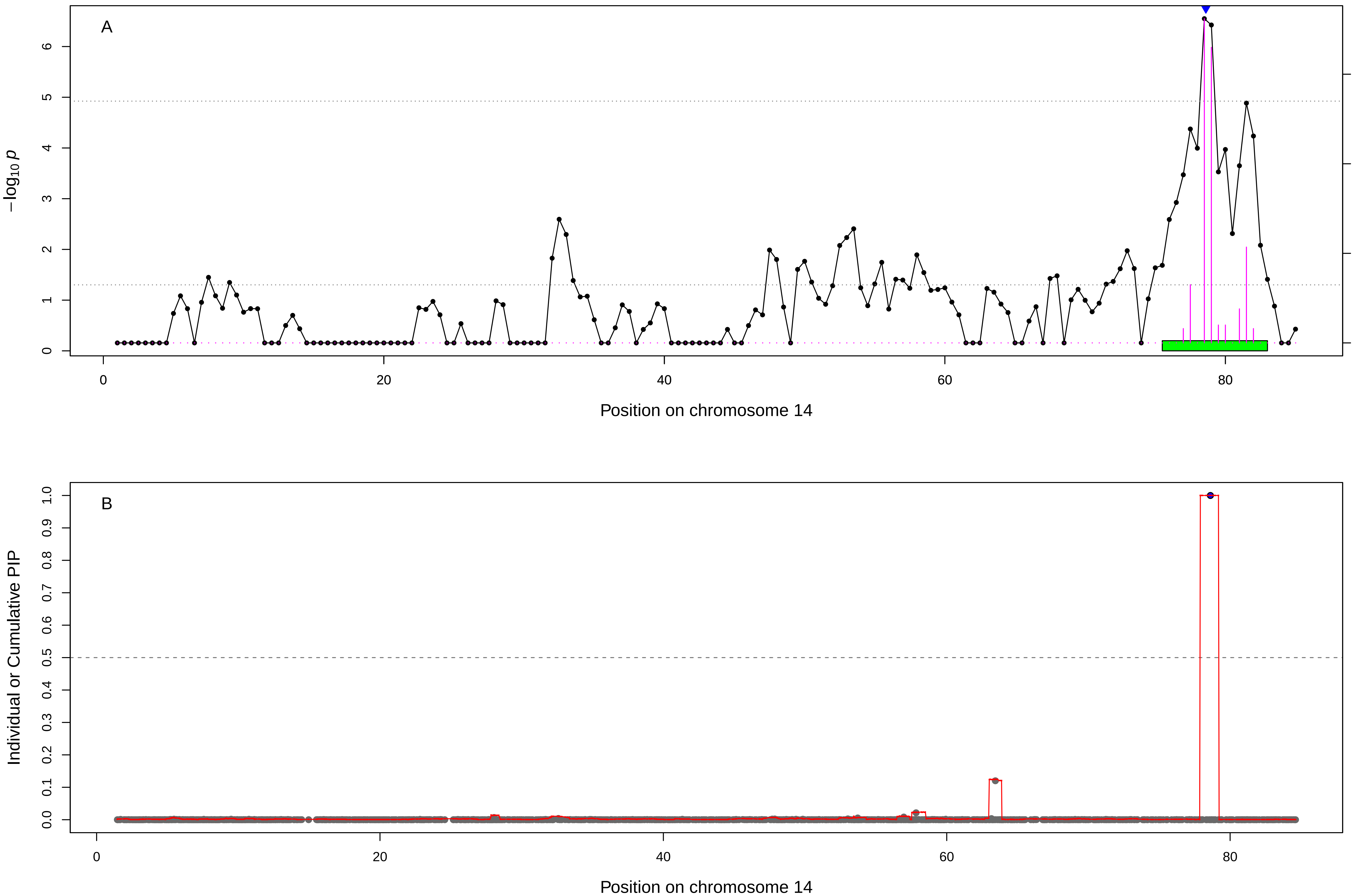

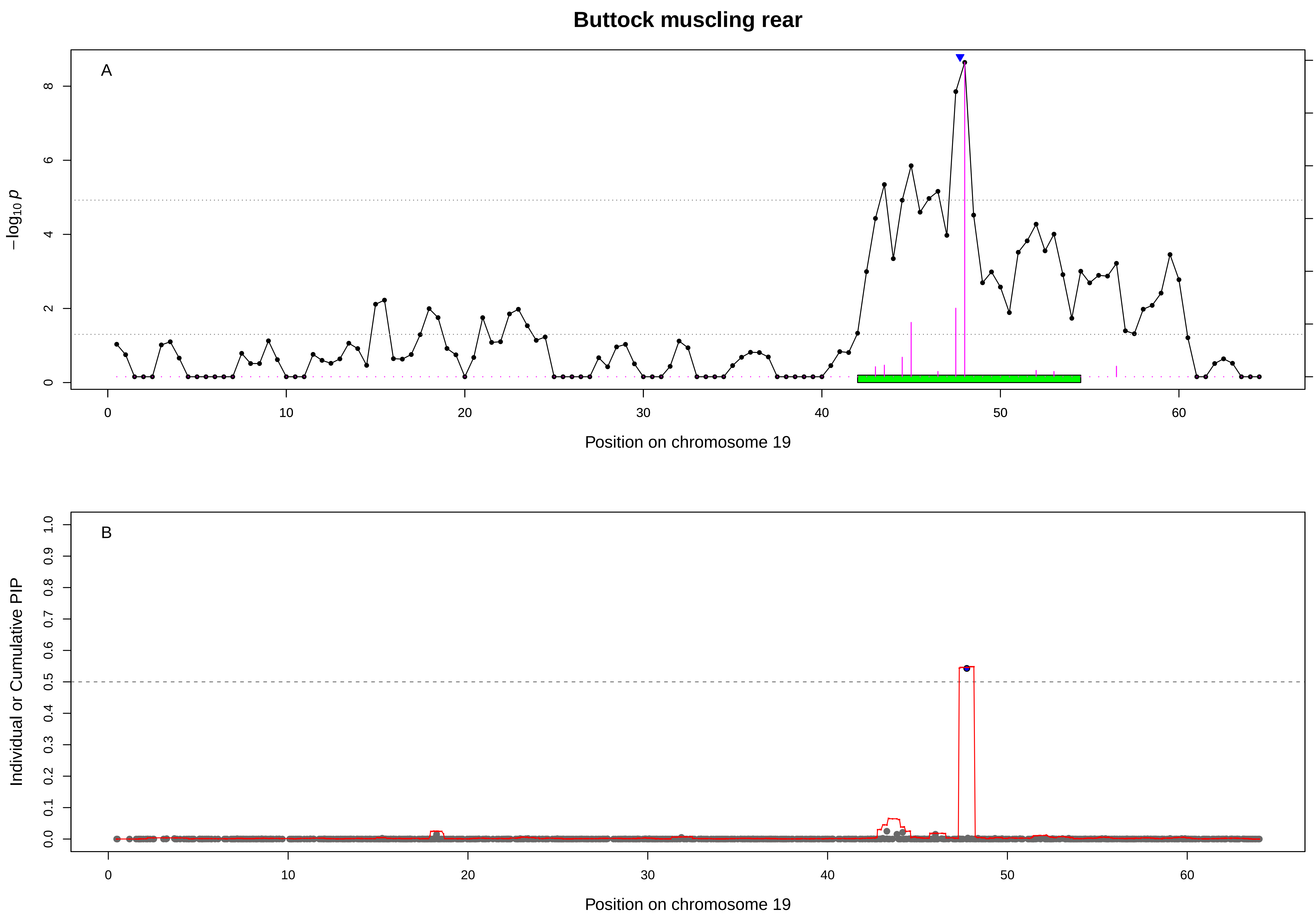

Buttock muscling rear
A
−log10 p
Position on chromosome 19
B
Individual or Cumulative PIP
Position on chromosome 19

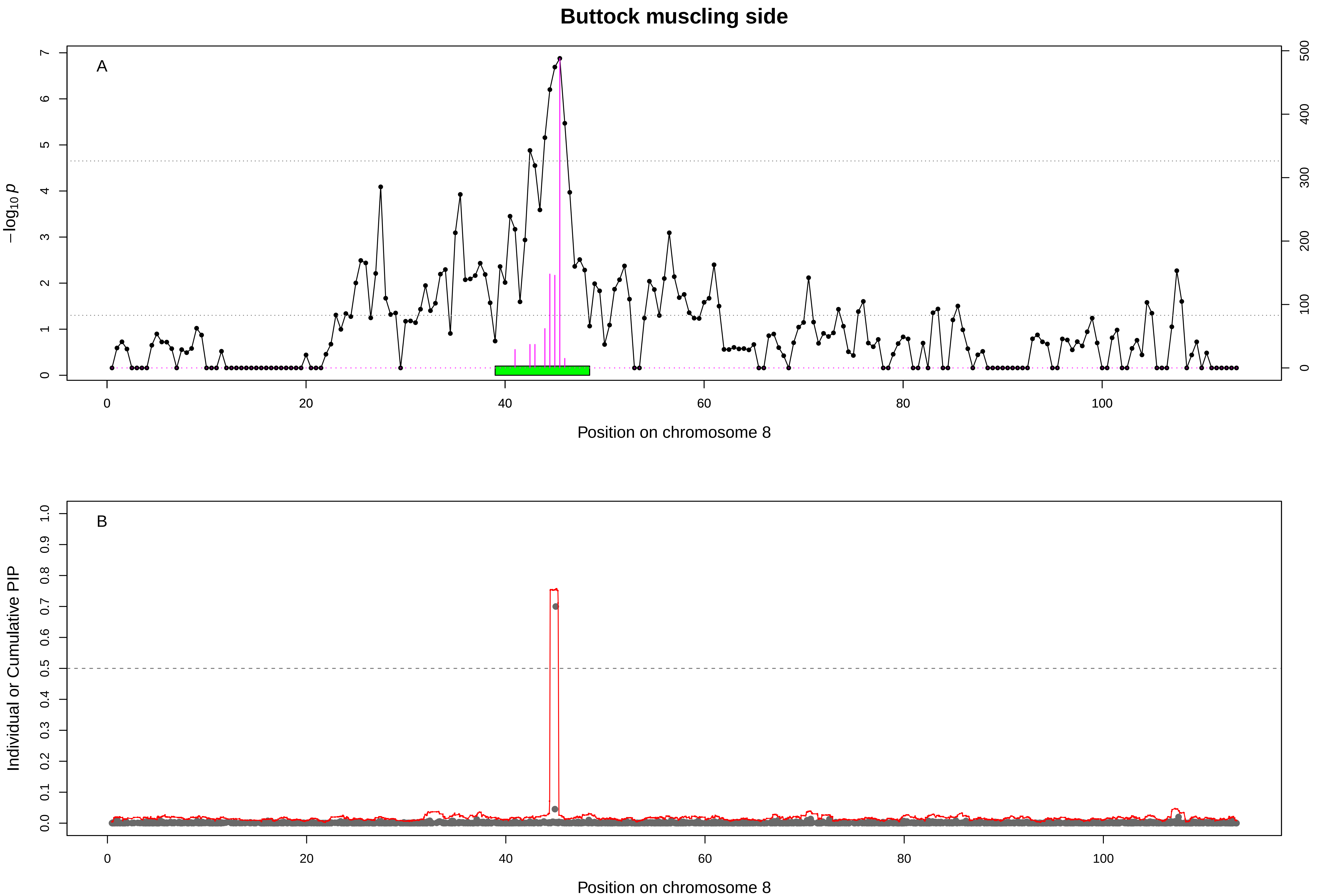
Buttock muscling side
A
−log10 p
Position on chromosome 8
0
20
40
60
80
100
0
1
2
3
4
5
6
7
0
100
200
300
400
500
B
Individual or Cumulative PIP
0.0
0.1
0.2
0.3
0.4
0.5
0.6
0.7
0.8
0.9
1.0
Position on chromosome 8

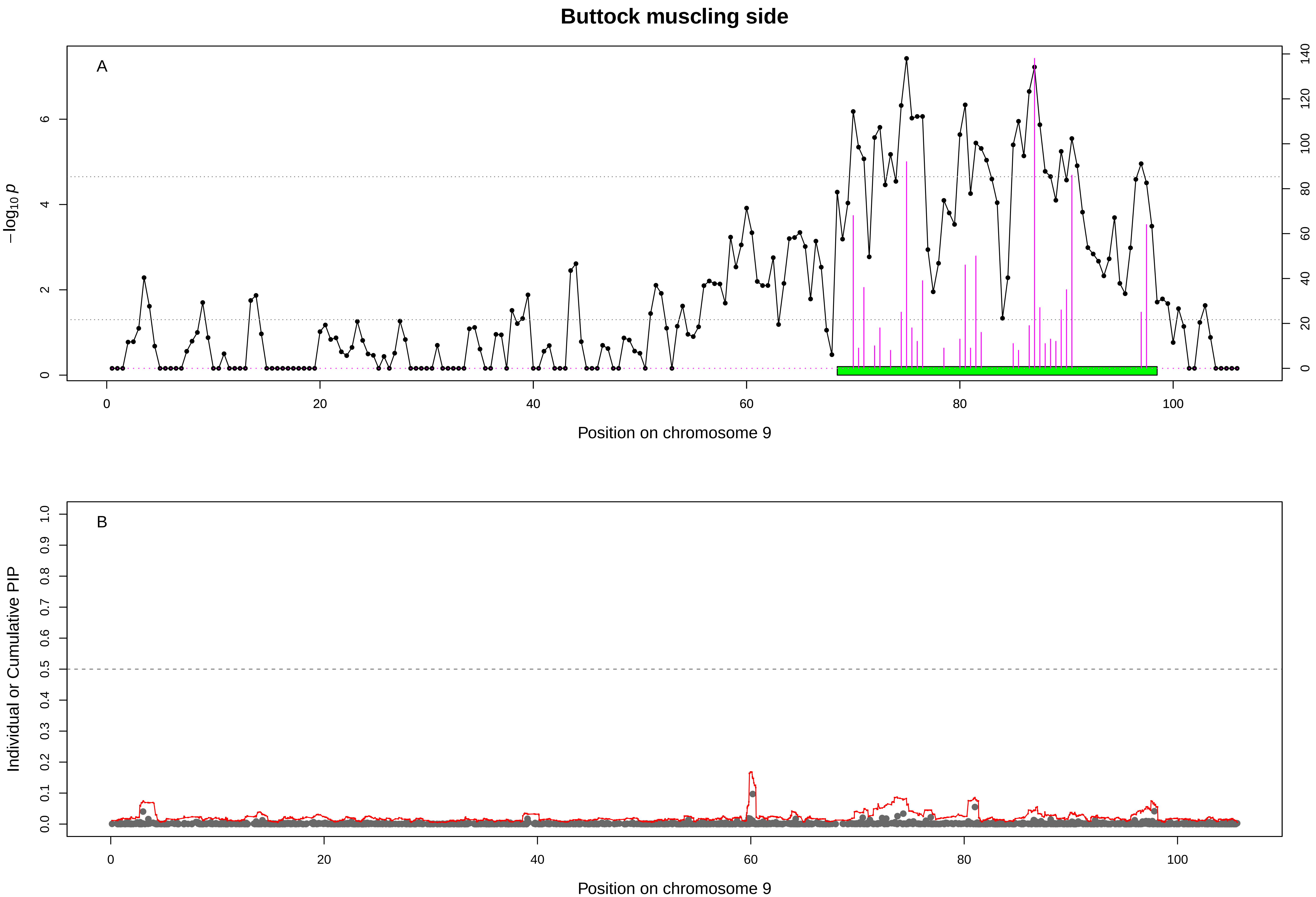

Buttock muscling side
A
$-\log_{10} p$
Position on chromosome 9
B
Individual or Cumulative PIP
Position on chromosome 9

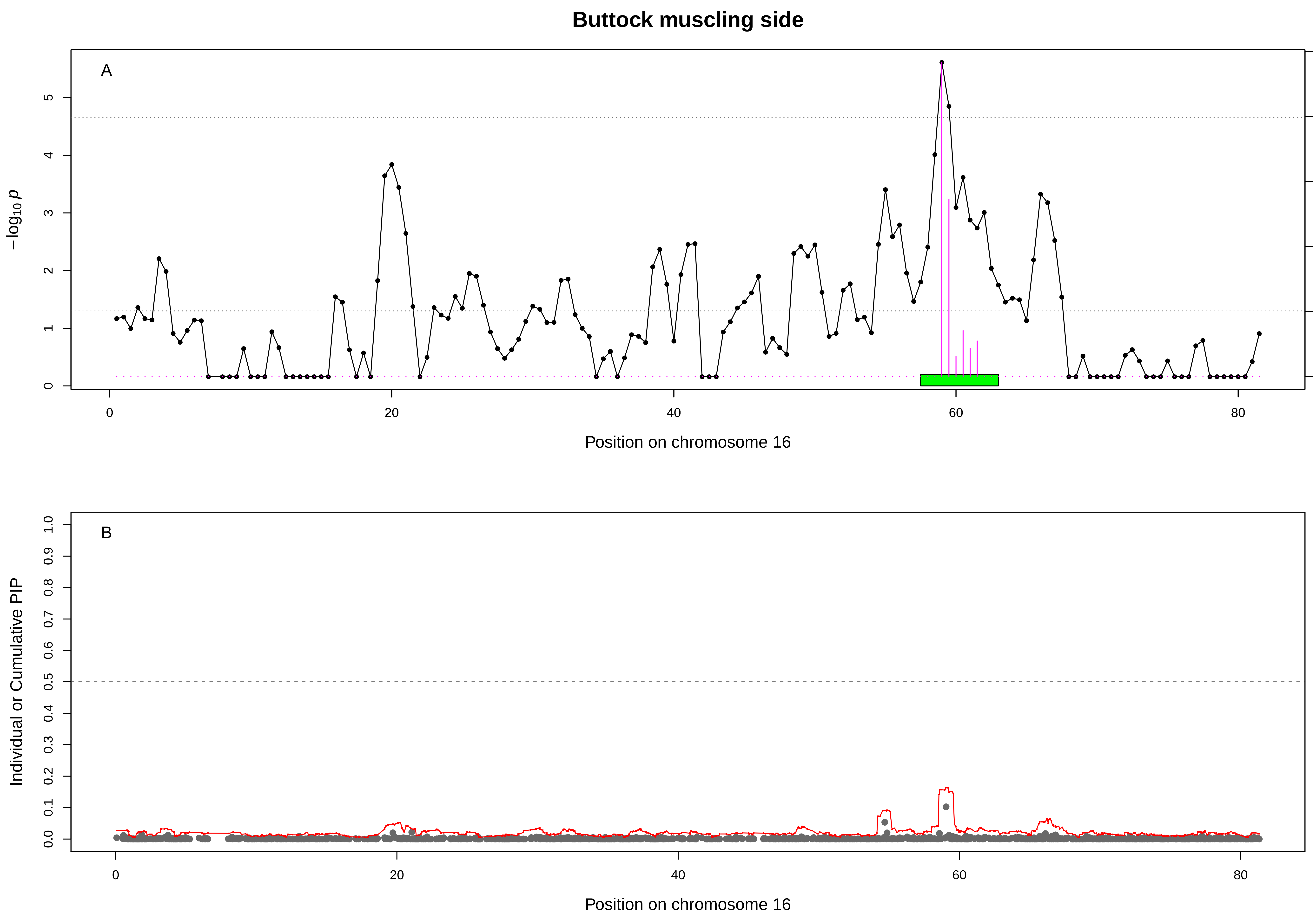

Buttock muscling side
A
−log10 p
Position on chromosome 16
B
Individual or Cumulative PIP
Position on chromosome 16

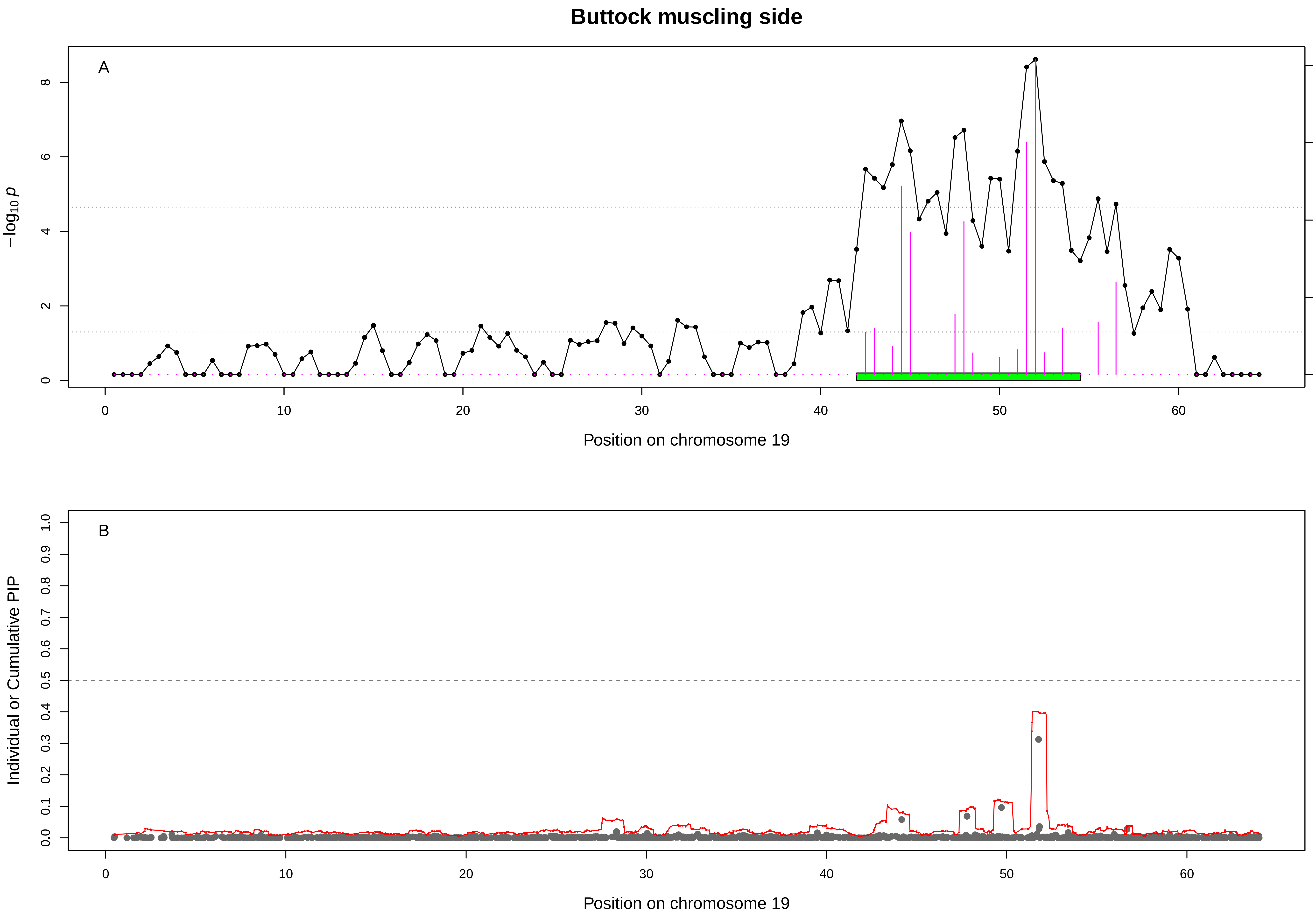

Buttock muscling side
A
−log10 p
Position on chromosome 19
B
Individual or Cumulative PIP
Position on chromosome 19

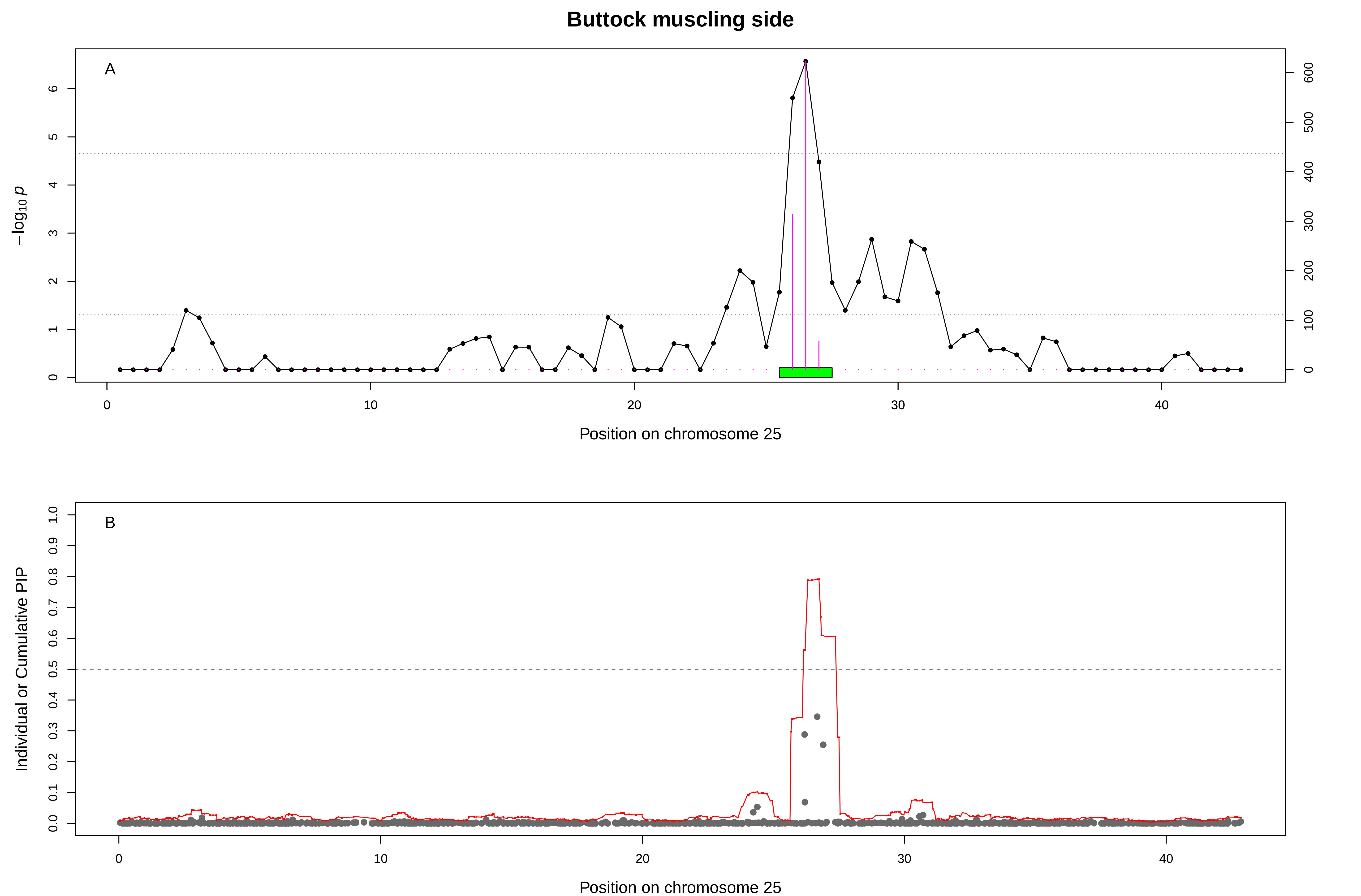

Buttock muscling side
A
$-\log_{10} p$
Position on chromosome 25
B
Individual or Cumulative PIP
Position on chromosome 25

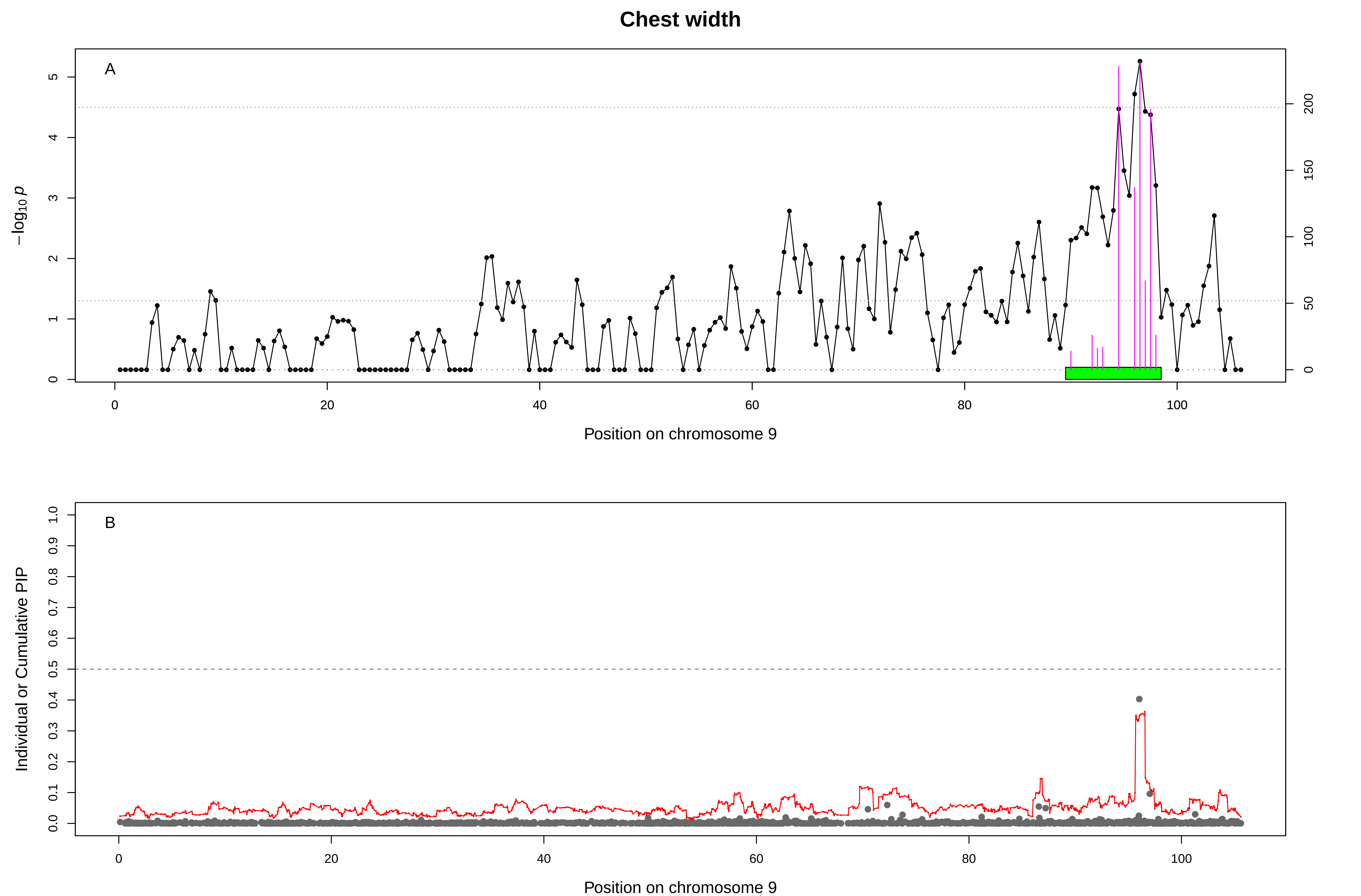
Chest width
A
$-\log_{10} p$
Position on chromosome 9
B
Individual or Cumulative PIP
Position on chromosome 9

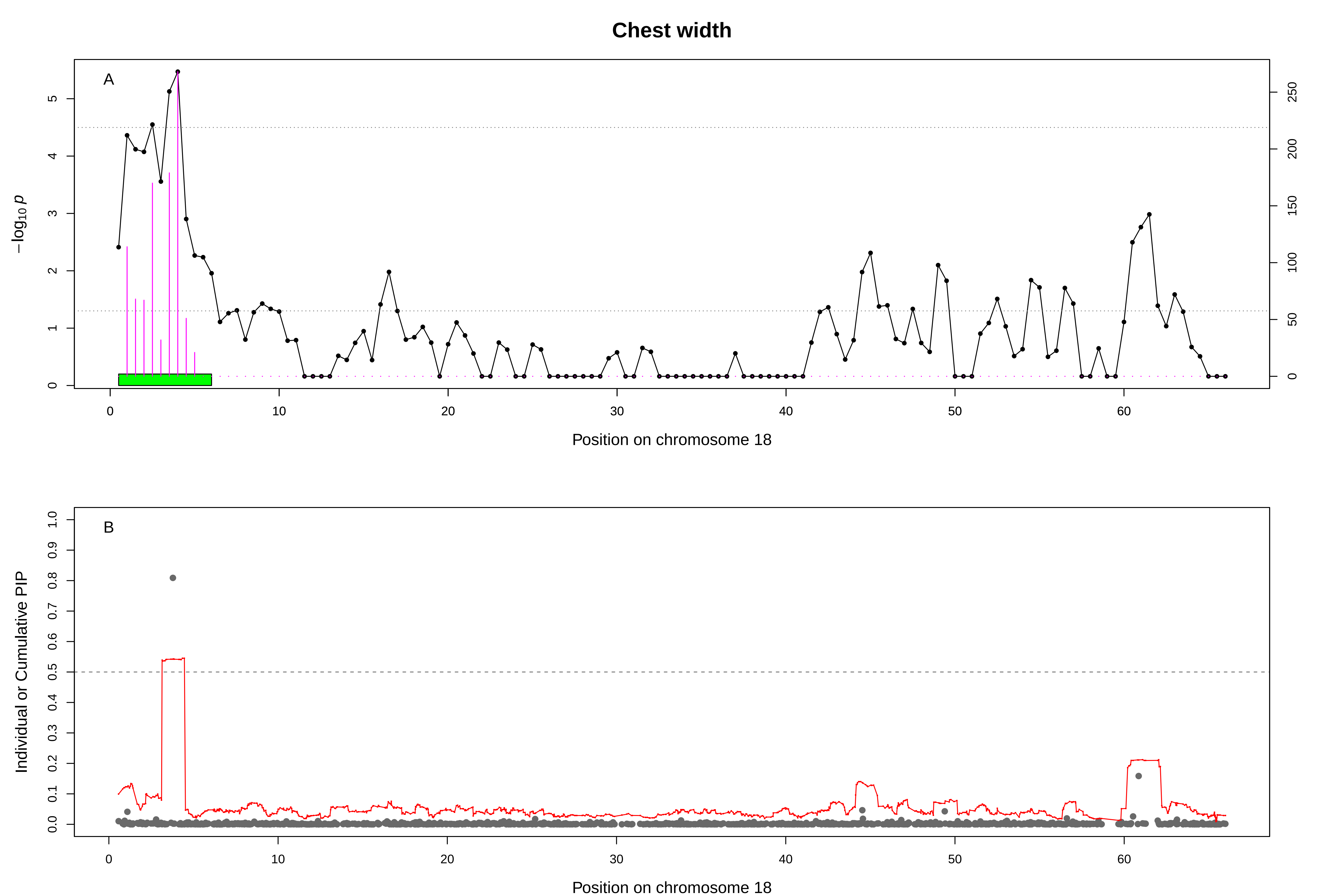

Chest width
A
B
−log10 p
Individual or Cumulative PIP
Position on chromosome 18
Position on chromosome 18

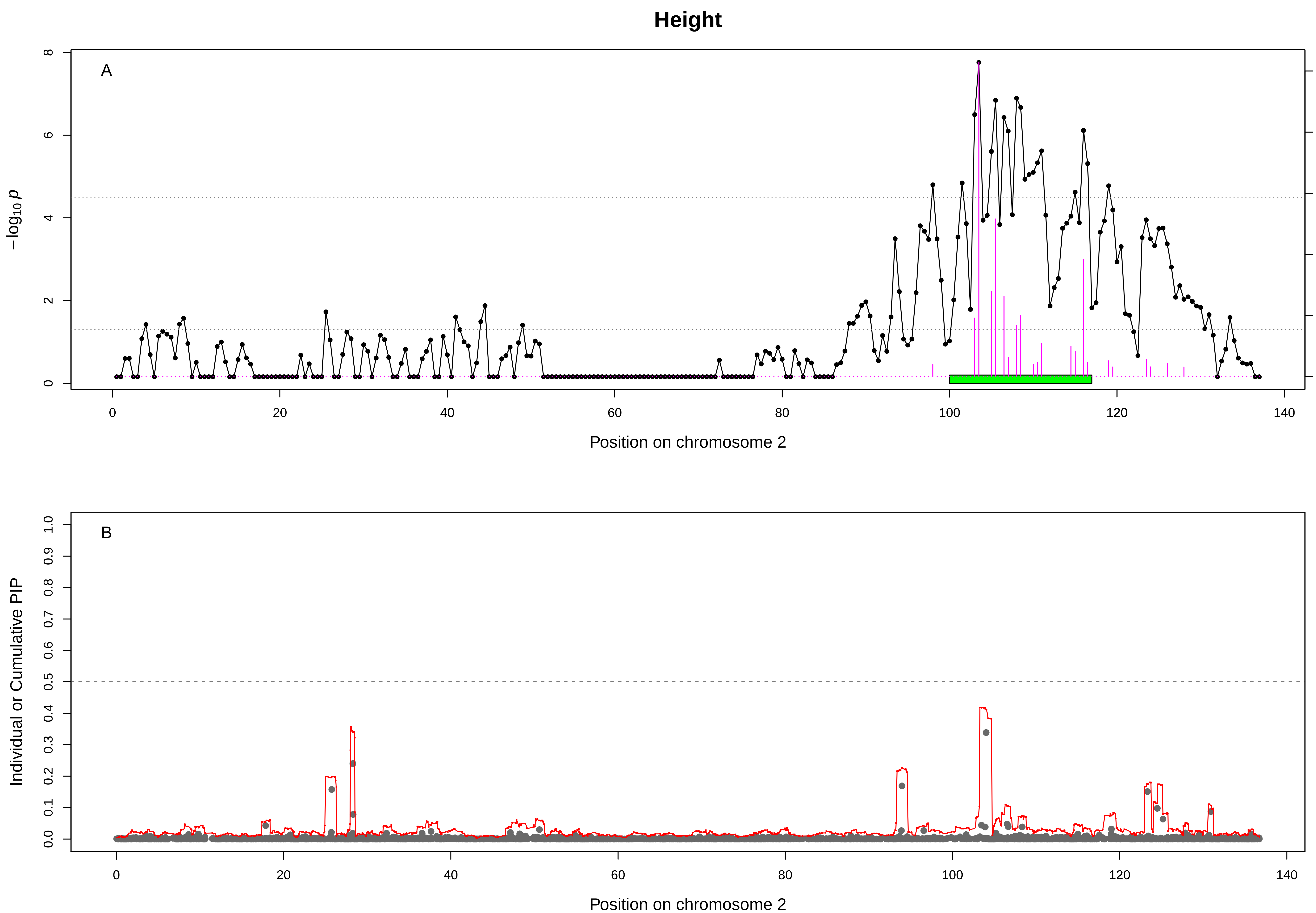
Height
A
B
−log10 p
Individual or Cumulative PIP
Position on chromosome 2
Position on chromosome 2

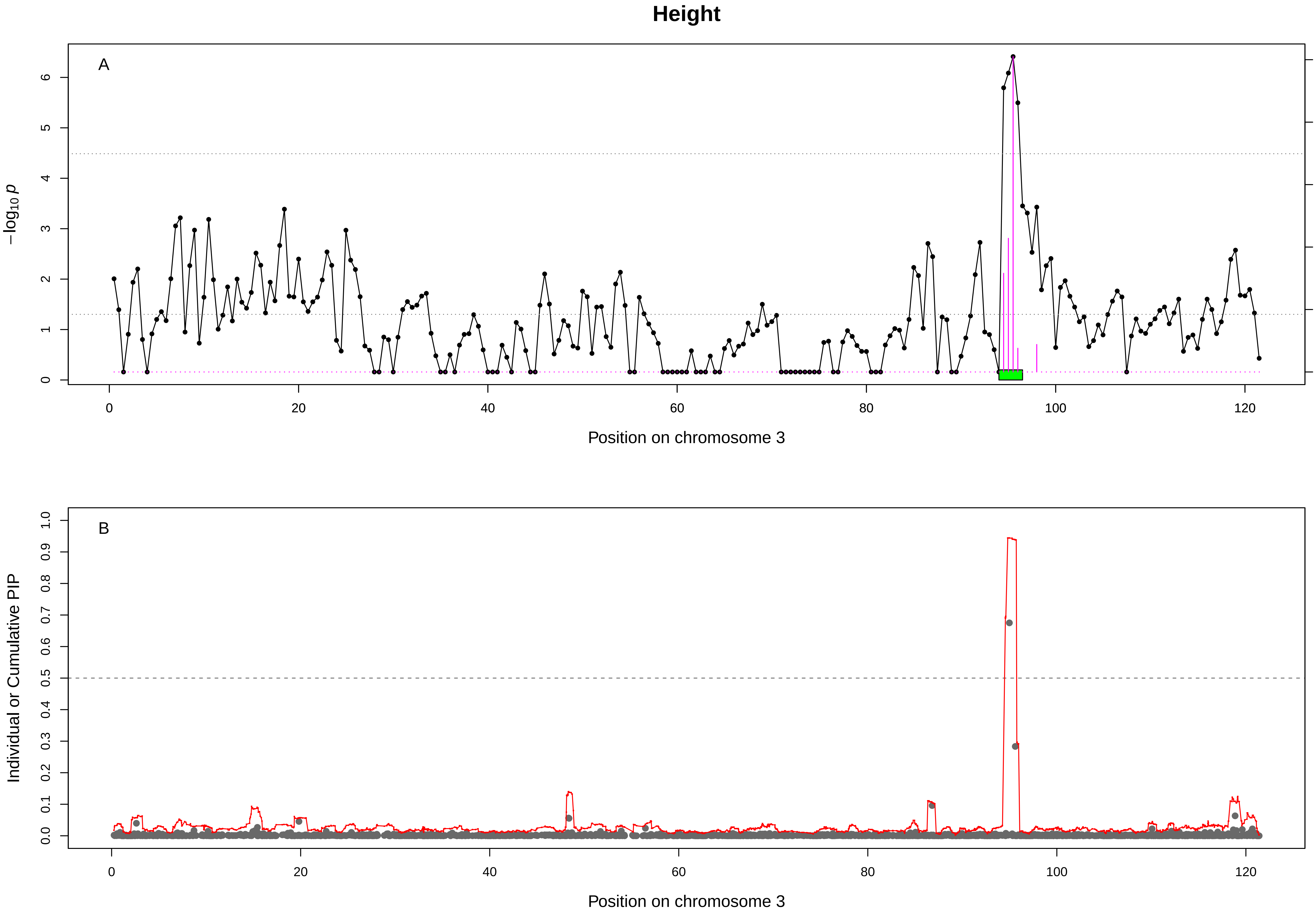

Height
A
$-\log_{10} p$
Position on chromosome 3
B
Individual or Cumulative PIP
Position on chromosome 3

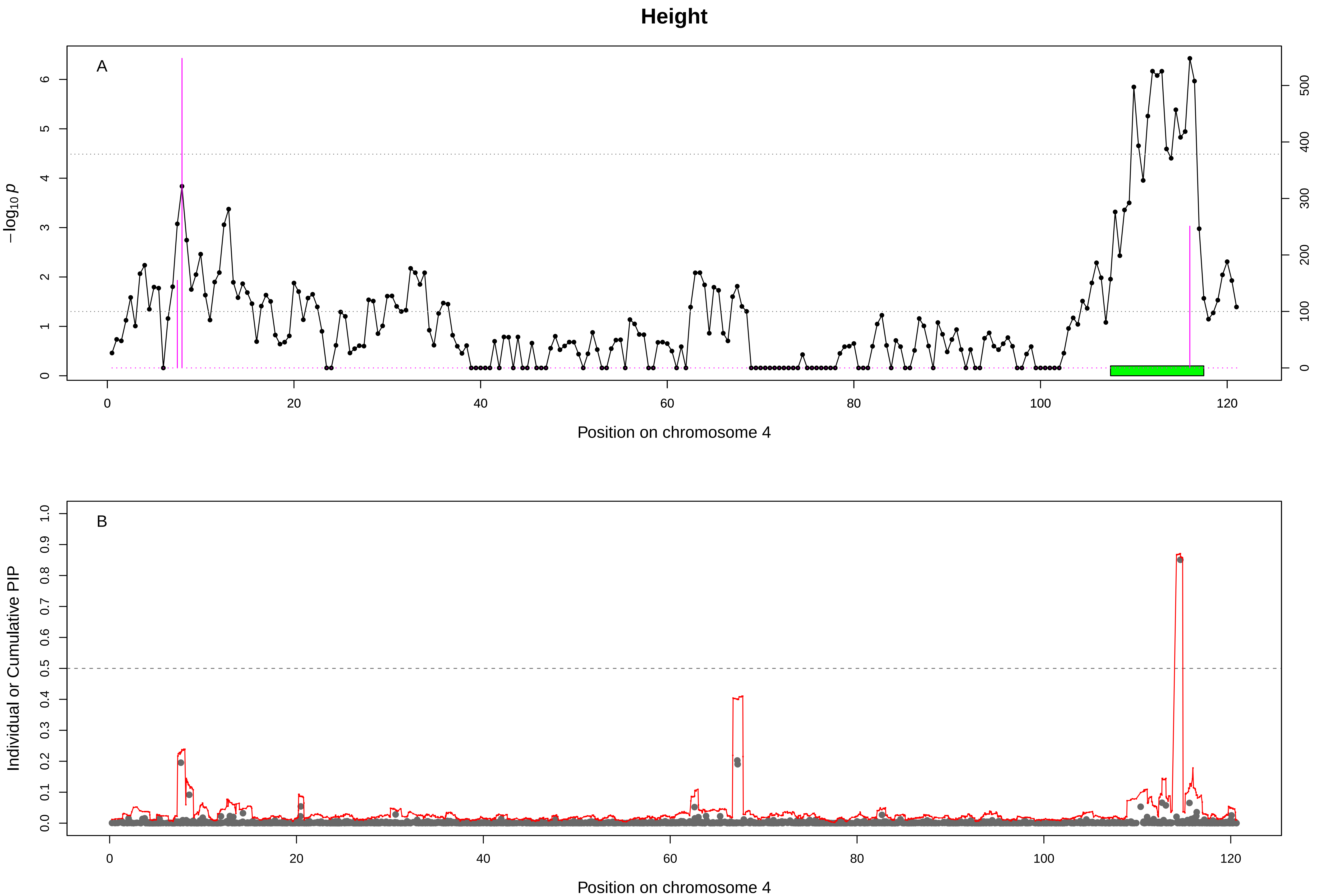

Height
A
B
−log10 p
Position on chromosome 4
Individual or Cumulative PIP

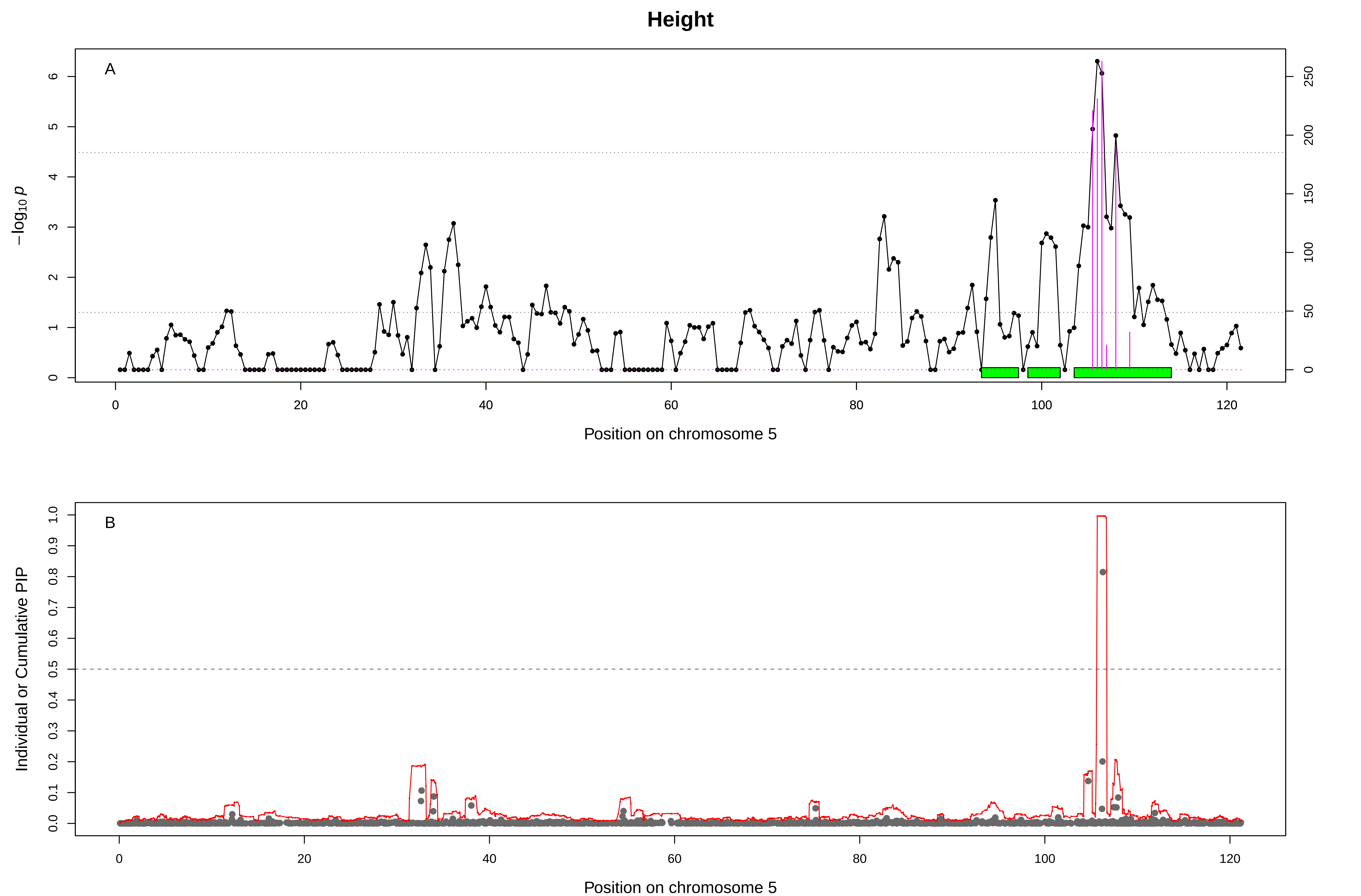
Height
A
B
−log10 p
Individual or Cumulative PIP
Position on chromosome 5
Position on chromosome 5

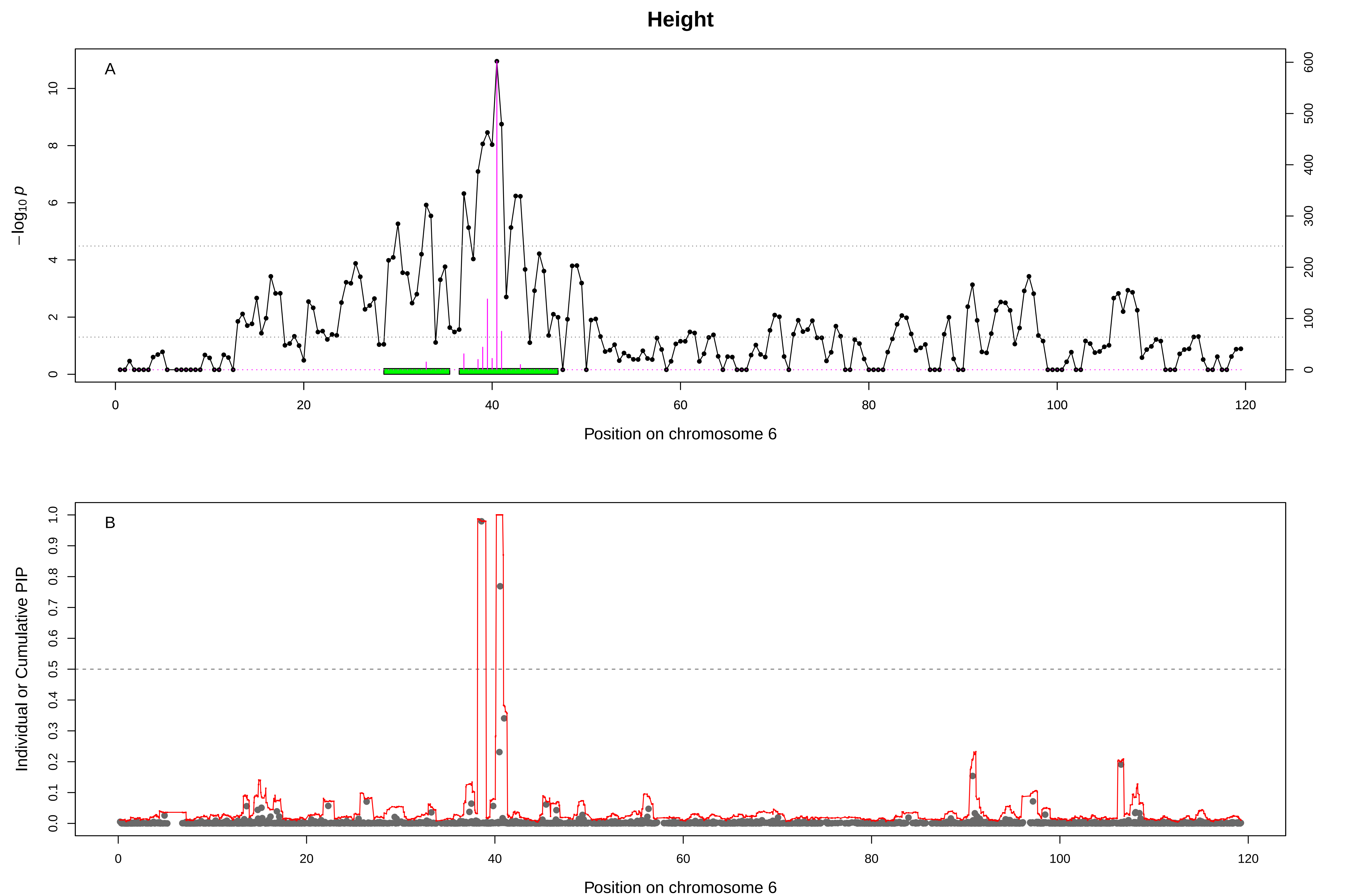

Height
A
−log10 p
Position on chromosome 6
B
Individual or Cumulative PIP
Position on chromosome 6

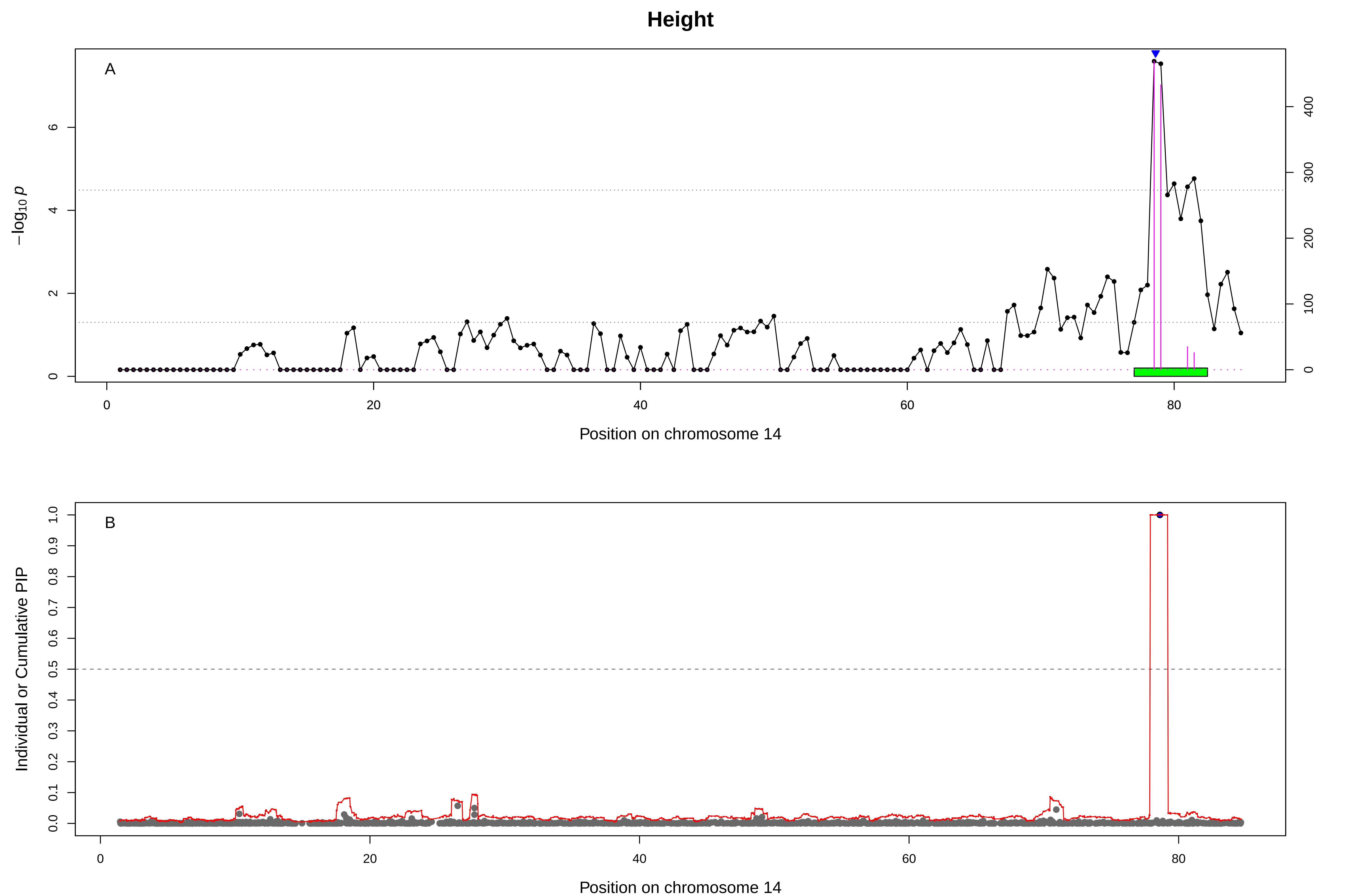
Height
A
−log10 p
Position on chromosome 14
B
Individual or Cumulative PIP
Position on chromosome 14

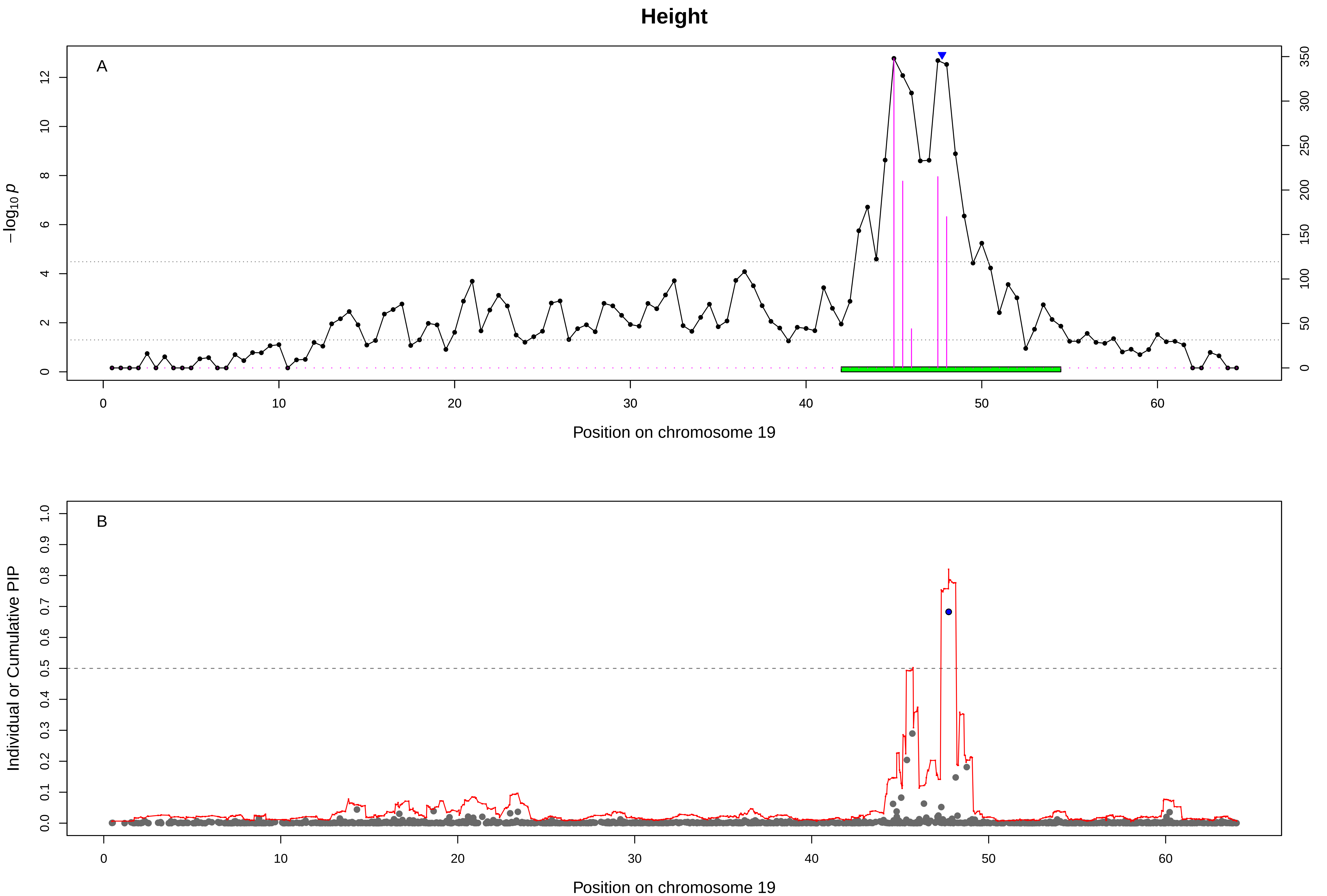
Height
A
B
−log10 p
Individual or Cumulative PIP
Position on chromosome 19
Position on chromosome 19

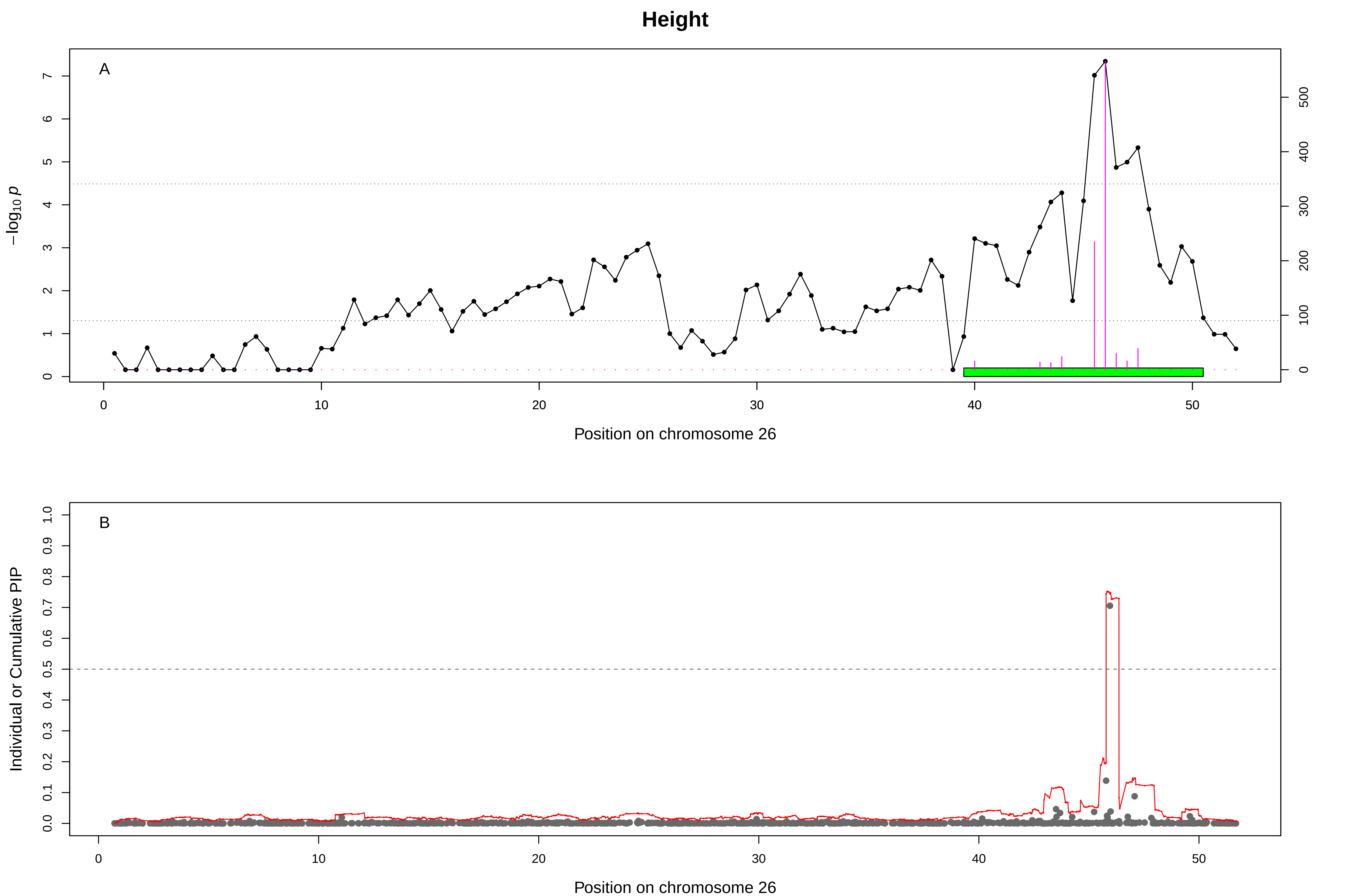
Height
A
B
$-\log_{10} p$
Individual or Cumulative PIP
Position on chromosome 26
Position on chromosome 26

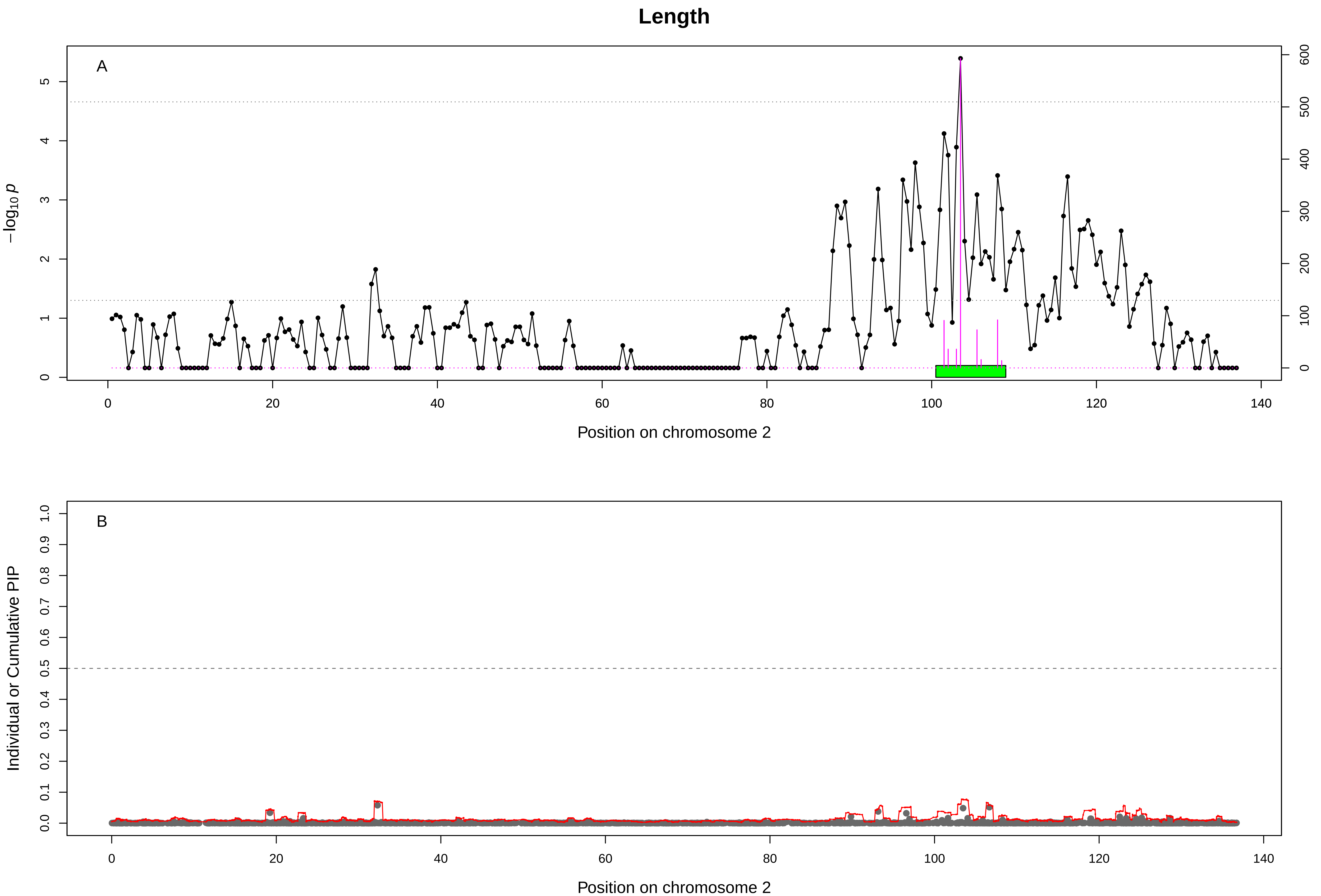

Length
A
B
−log10 p
Individual or Cumulative PIP
Position on chromosome 2

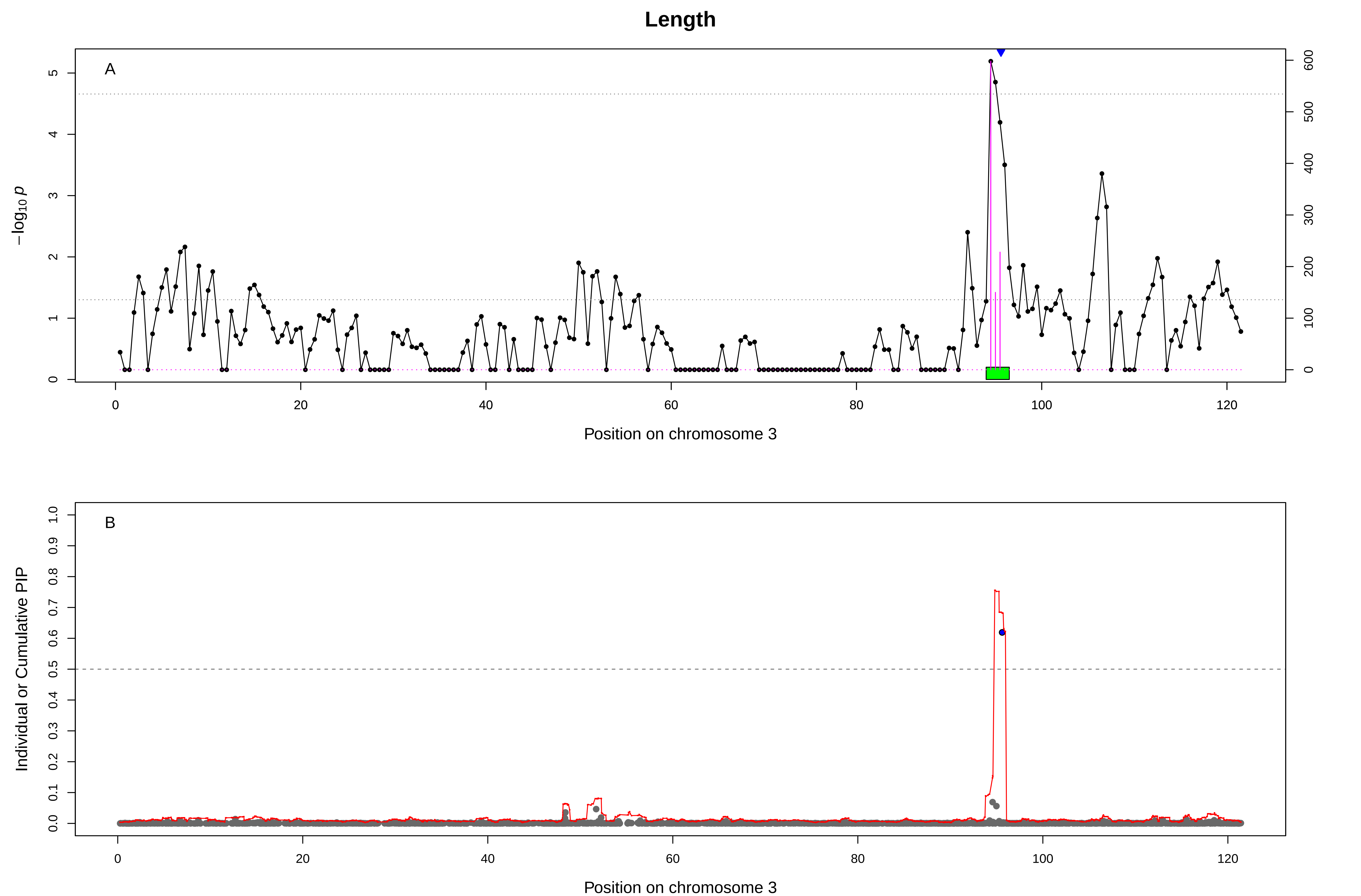

Length
A
B
$-\log_{10} p$
Position on chromosome 3
Individual or Cumulative PIP
Position on chromosome 3

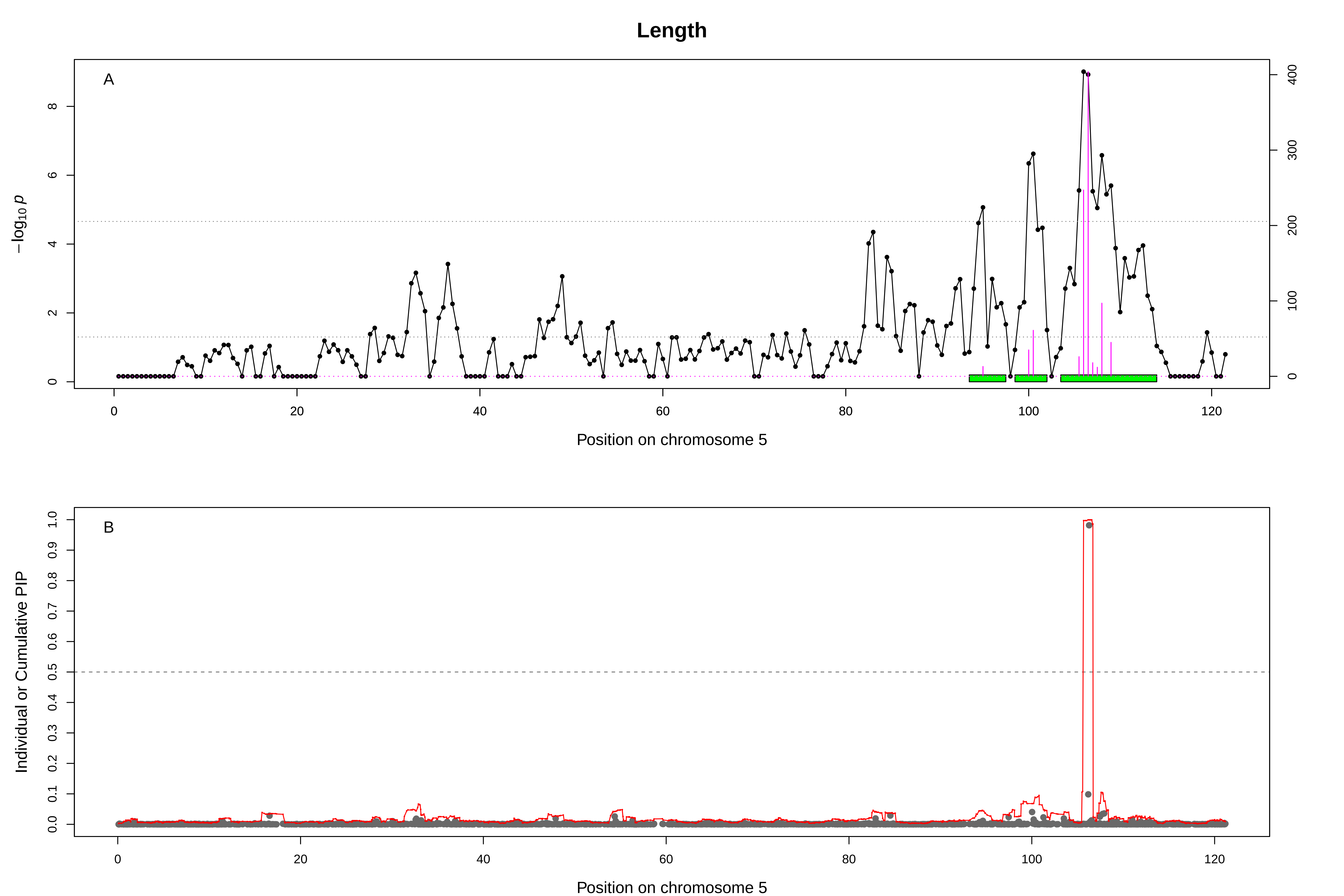

Length
A
−log10 p
Position on chromosome 5
B
Individual or Cumulative PIP
Position on chromosome 5

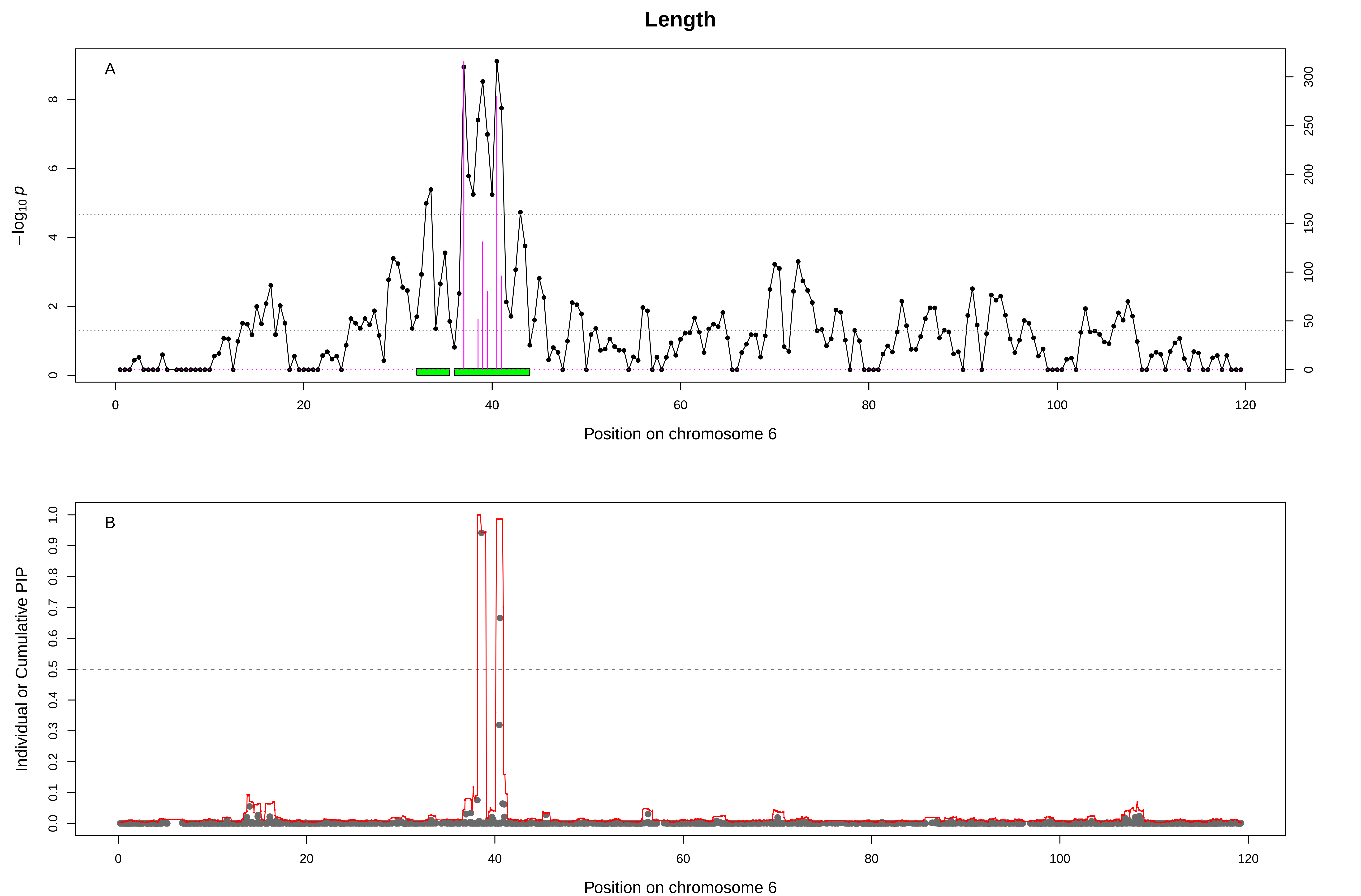

Length
A
−log10 p
Position on chromosome 6
B
Individual or Cumulative PIP
Position on chromosome 6

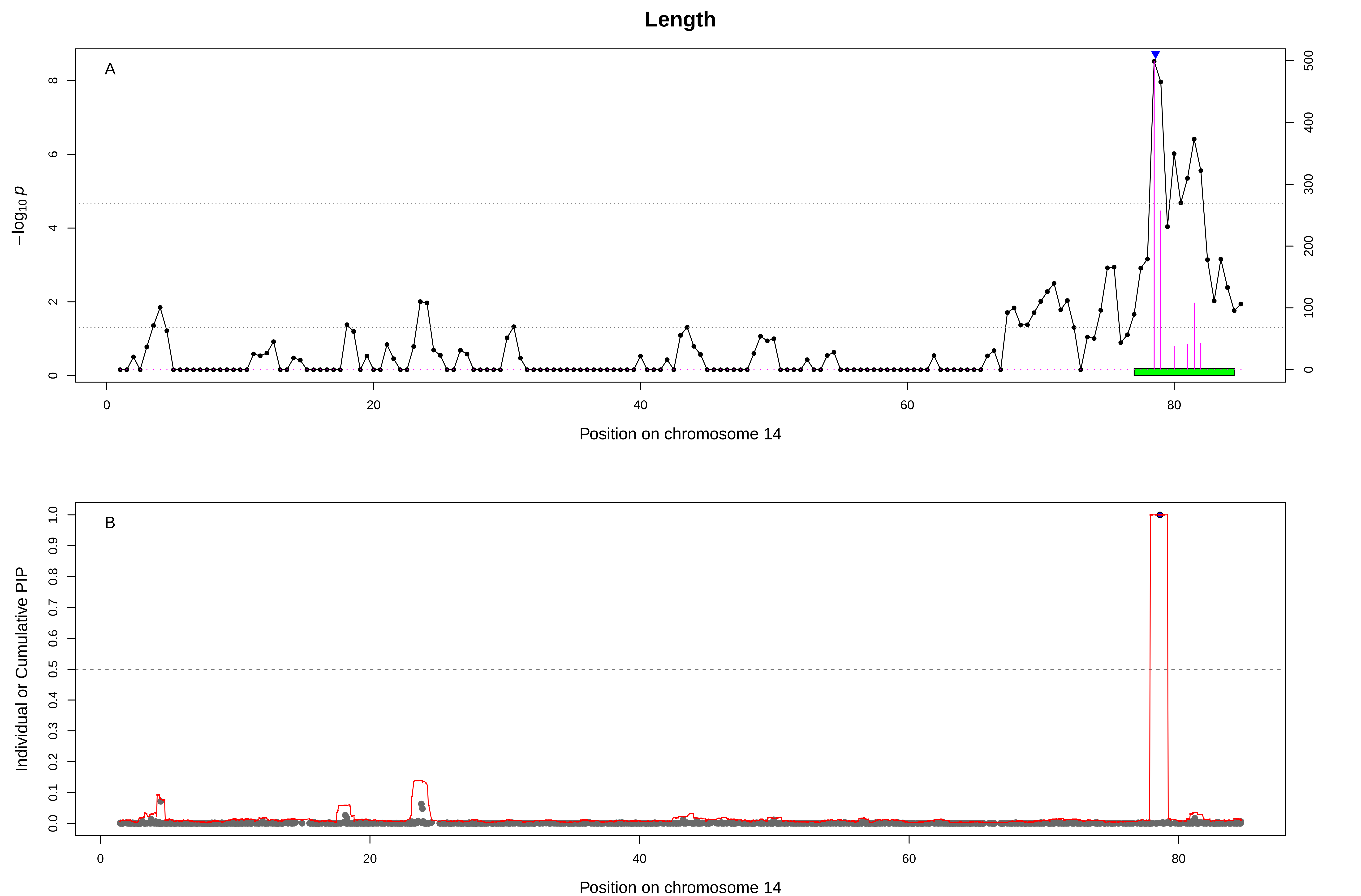
Length
A
B
$-\log_{10} p$
Individual or Cumulative PIP
Position on chromosome 14
Position on chromosome 14
0
20
40
60
80
0
2
4
6
8
0
100
200
300
400
500
0.0
0.1
0.2
0.3
0.4
0.5
0.6
0.7
0.8
0.9
1.0

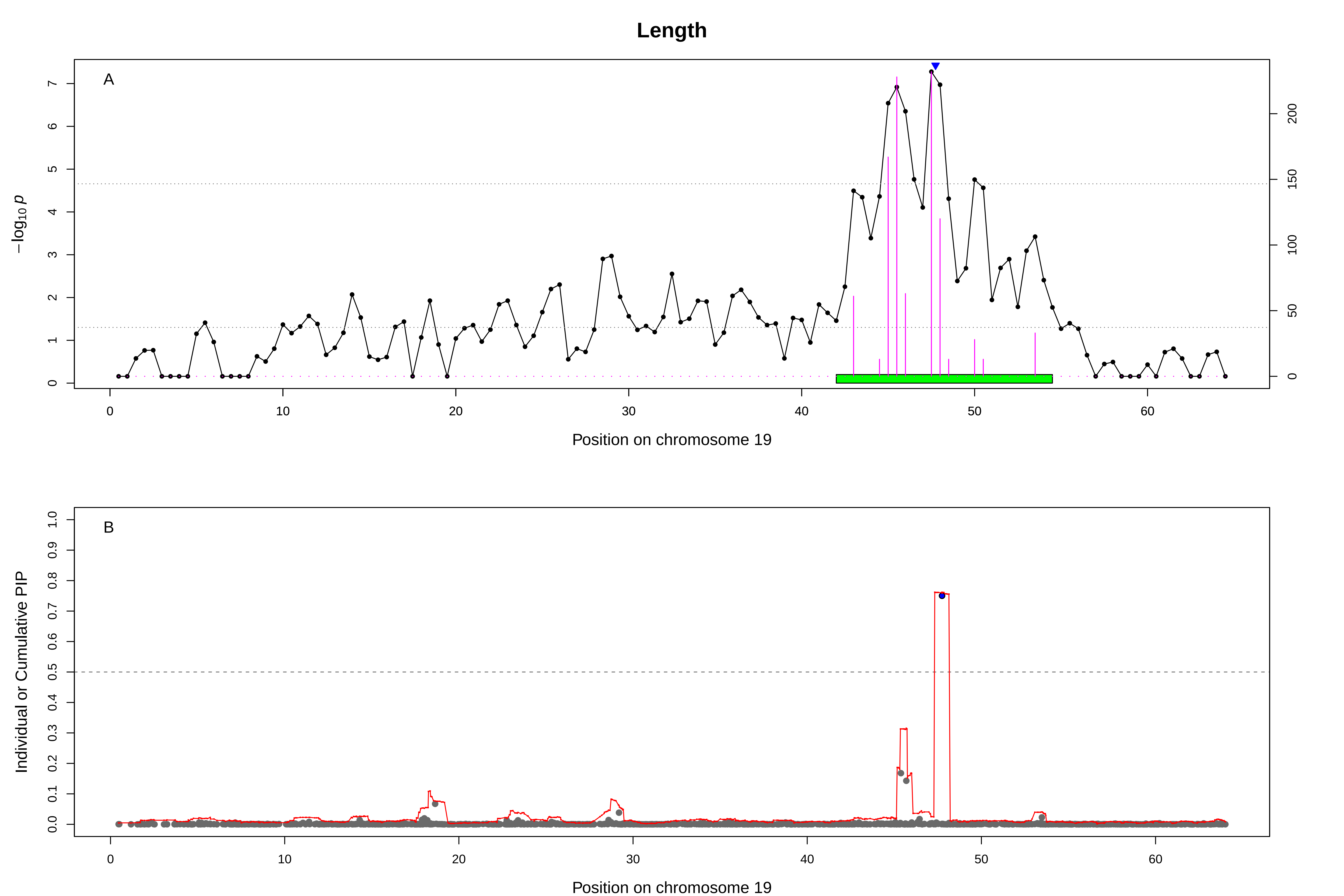

Length
A
$-\log_{10} p$
Position on chromosome 19
B
Individual or Cumulative PIP
Position on chromosome 19

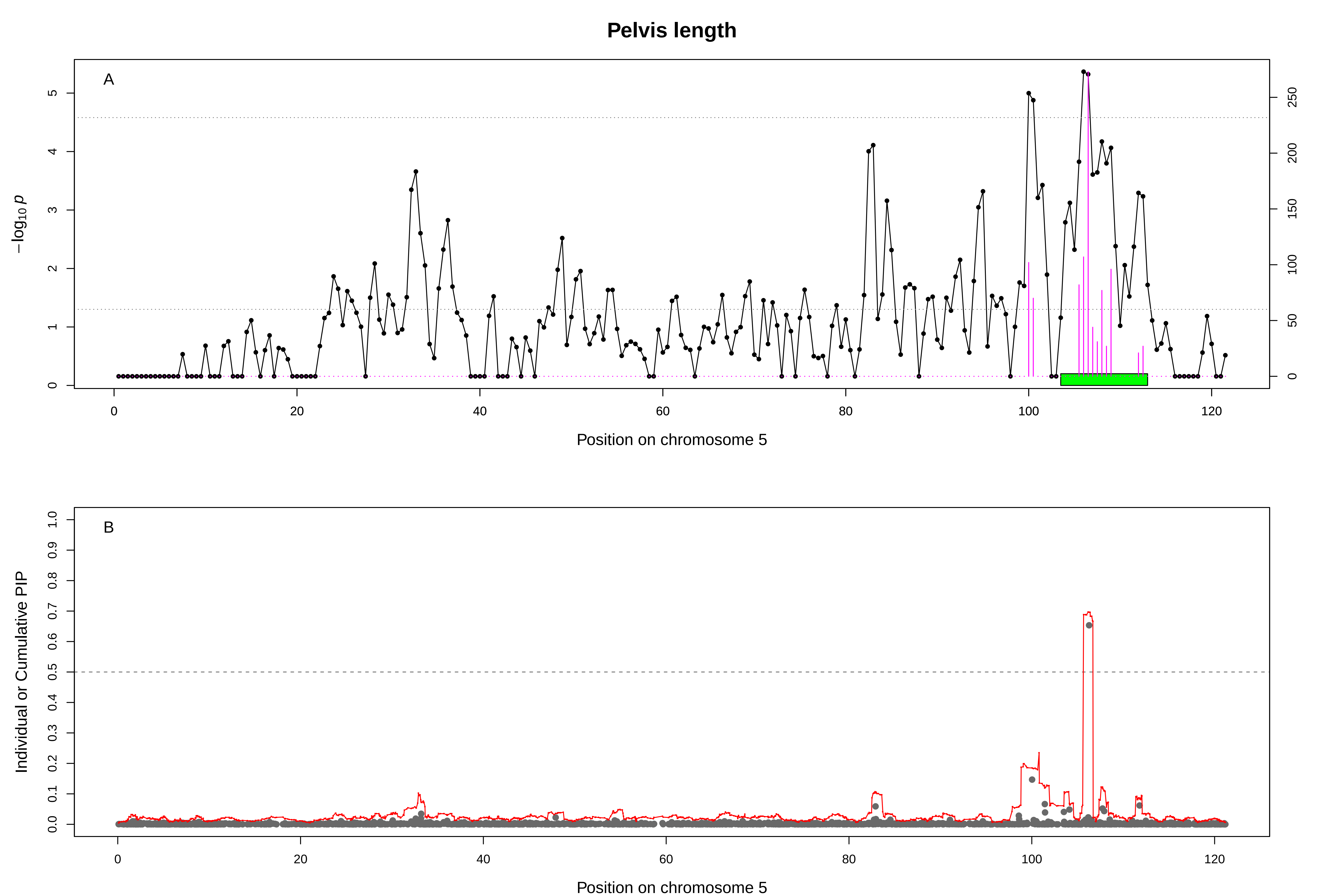

Pelvis length
A
B
−log10 p
Individual or Cumulative PIP
Position on chromosome 5
Position on chromosome 5

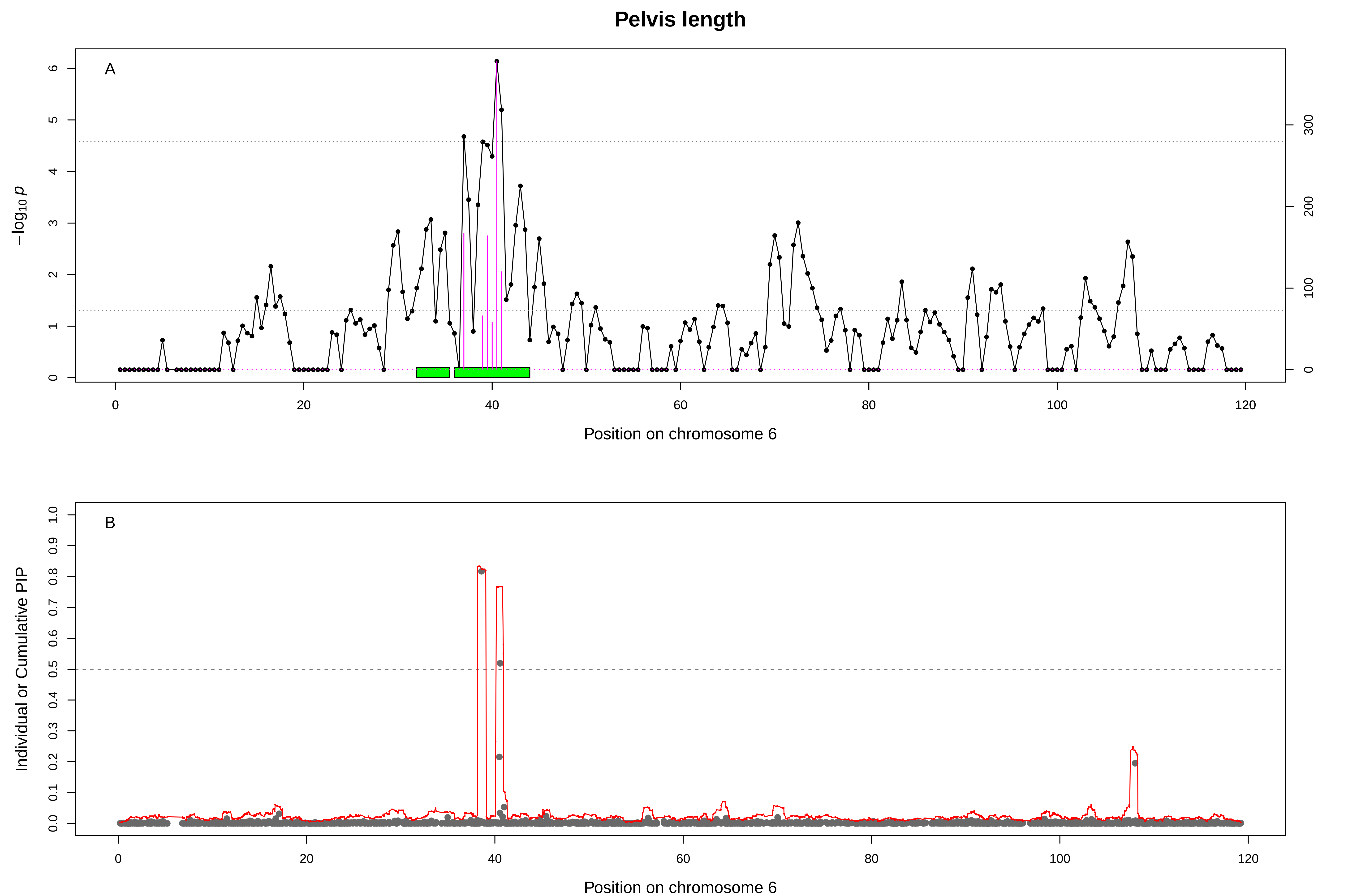
Pelvis length
A
−log10 p
Position on chromosome 6
B
Individual or Cumulative PIP
Position on chromosome 6

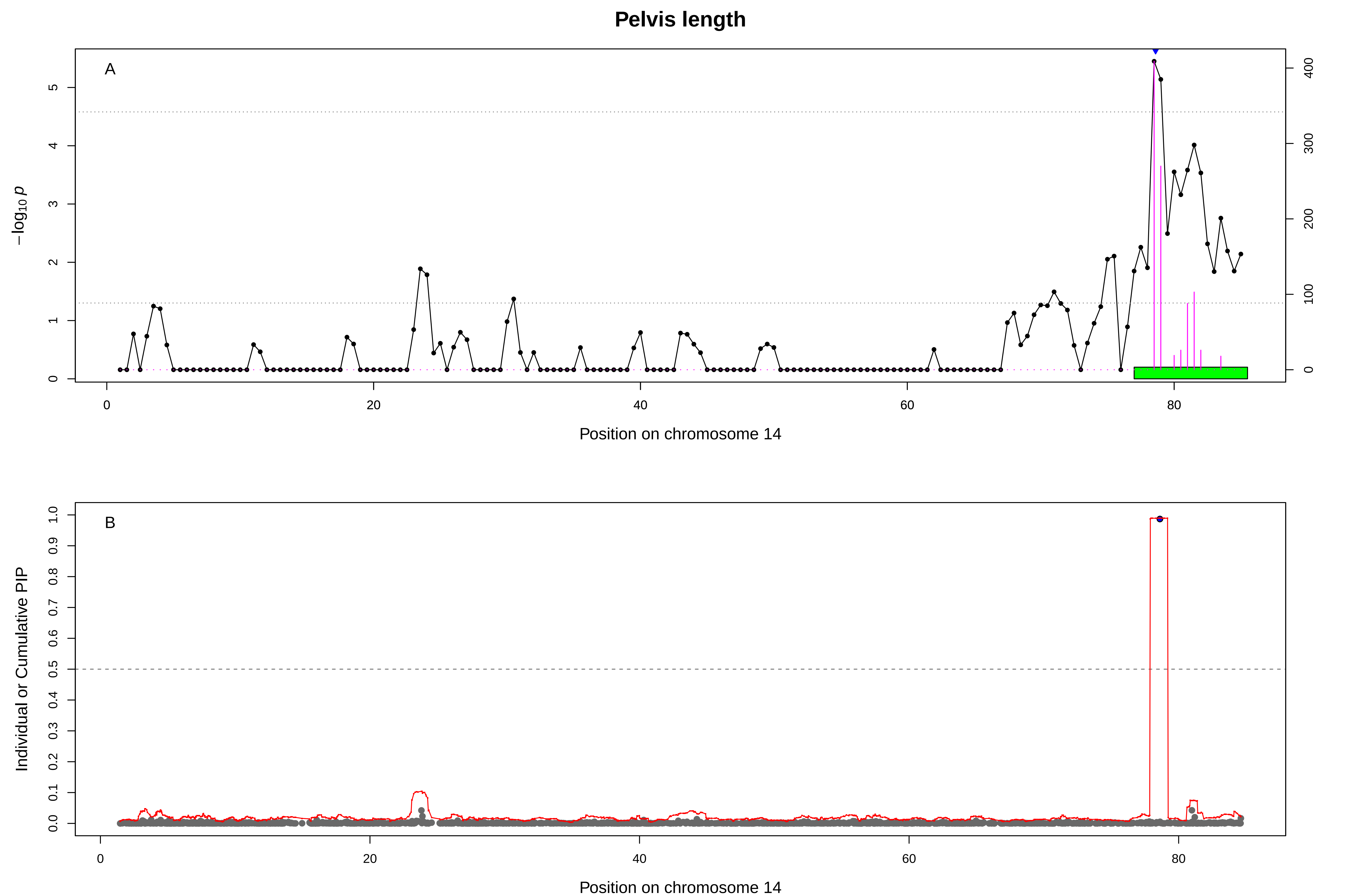
Pelvis length
A
−log10 p
Position on chromosome 14
B
Individual or Cumulative PIP
Position on chromosome 14

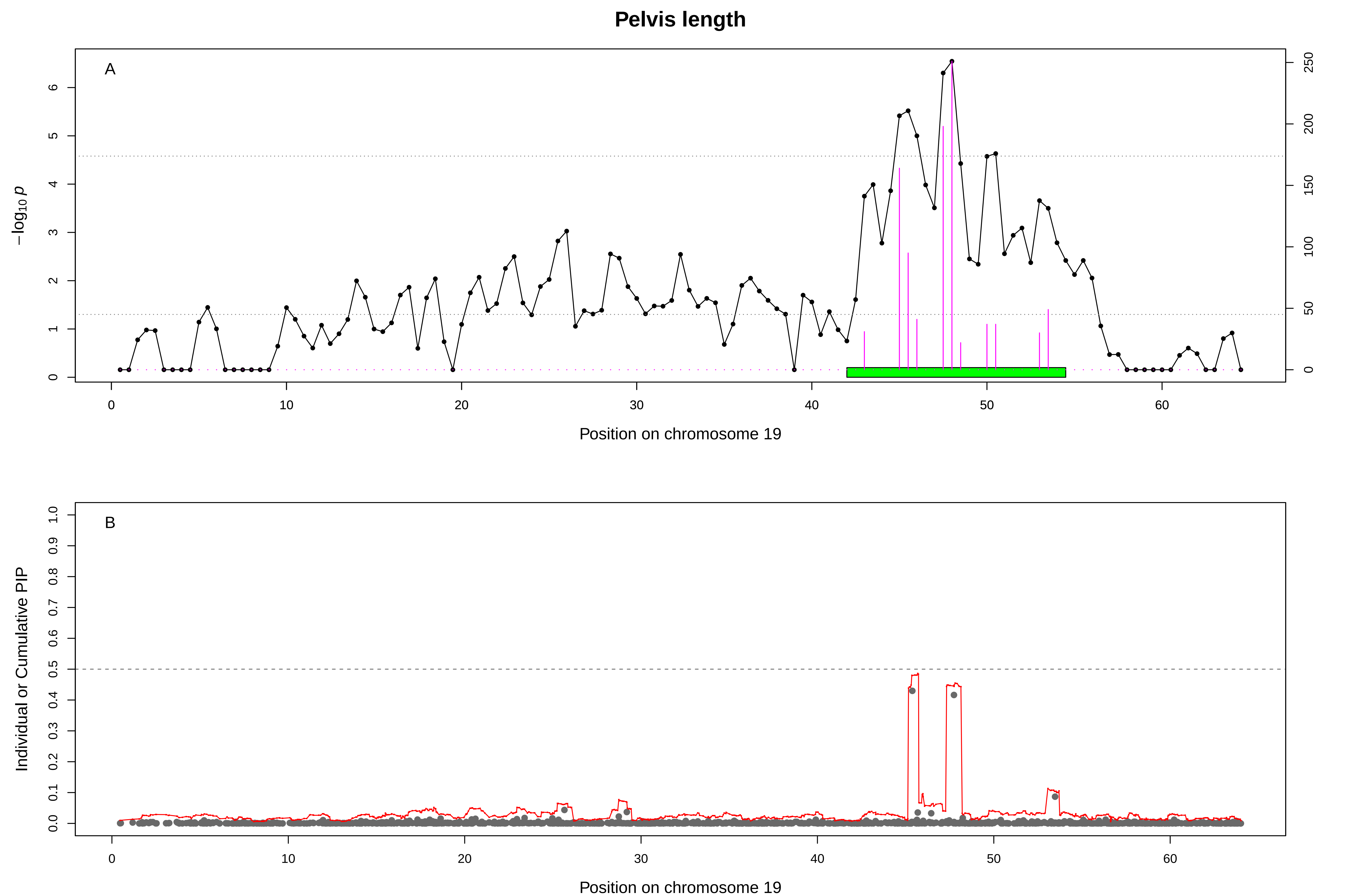
Pelvis length
A
B
−log10 p
Individual or Cumulative PIP
Position on chromosome 19
Position on chromosome 19

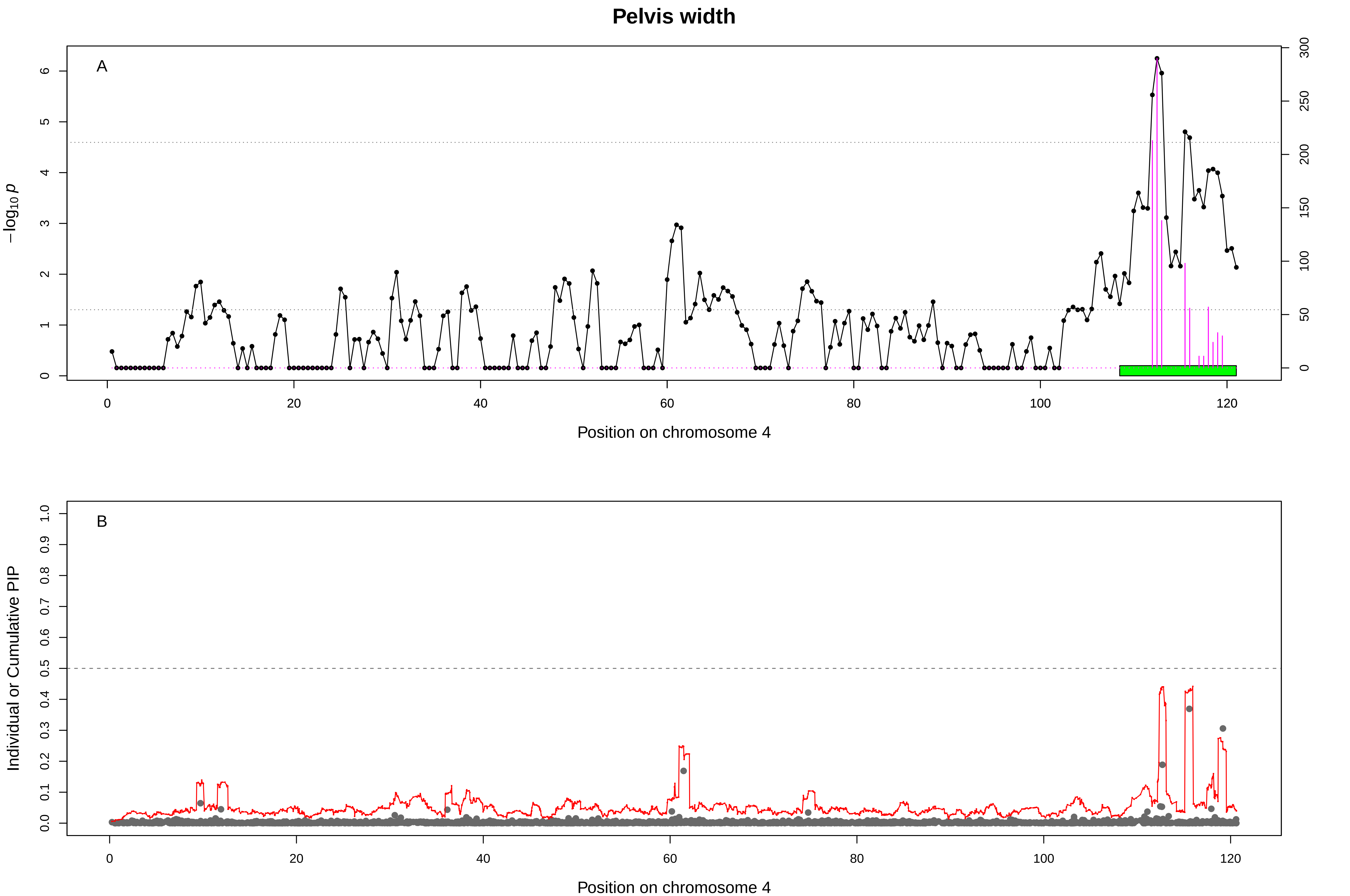

Pelvis width
A
−log10 p
Position on chromosome 4
B
Individual or Cumulative PIP
Position on chromosome 4

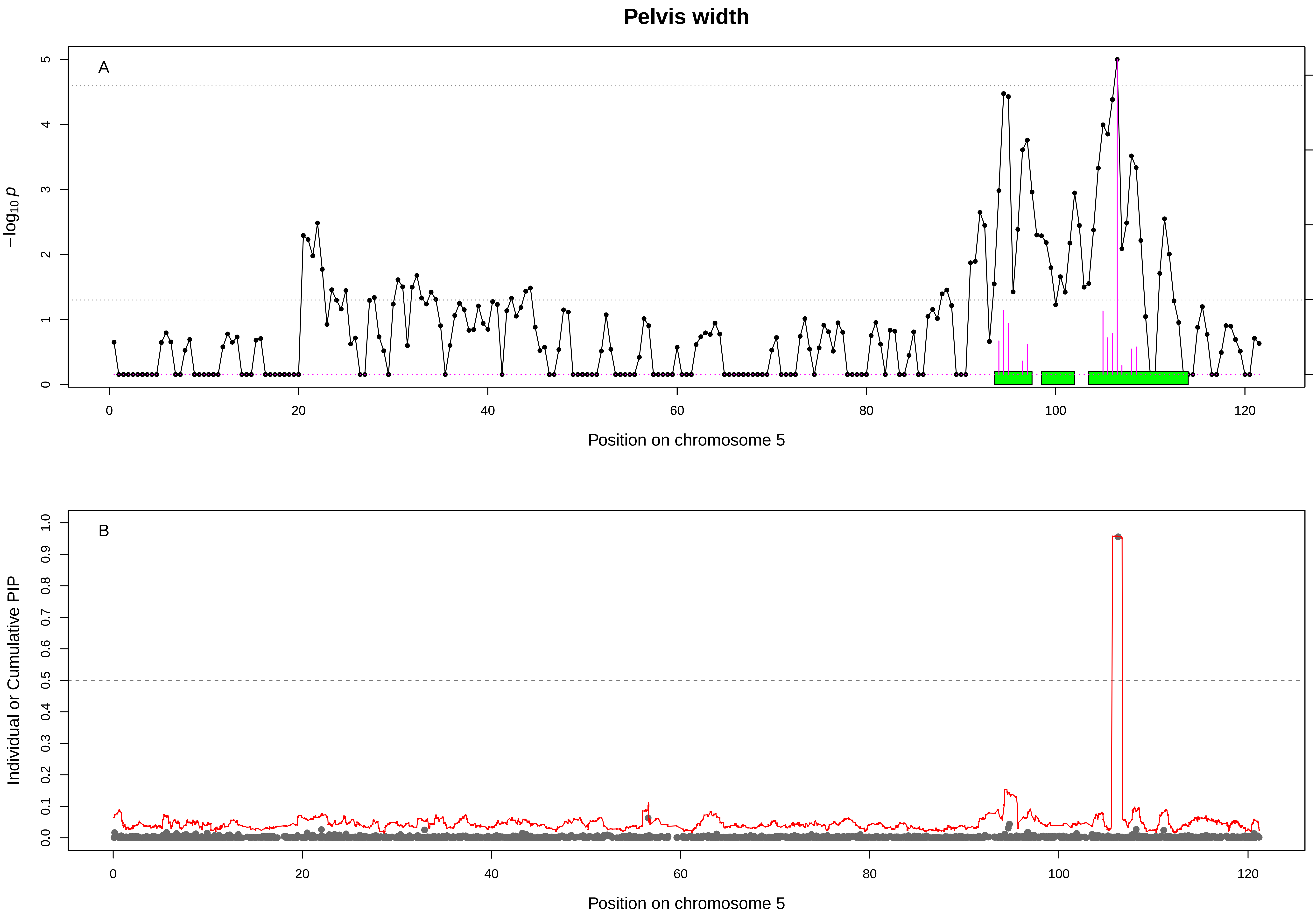

Pelvis width
A
B
−log10 p
Individual or Cumulative PIP
Position on chromosome 5
Position on chromosome 5

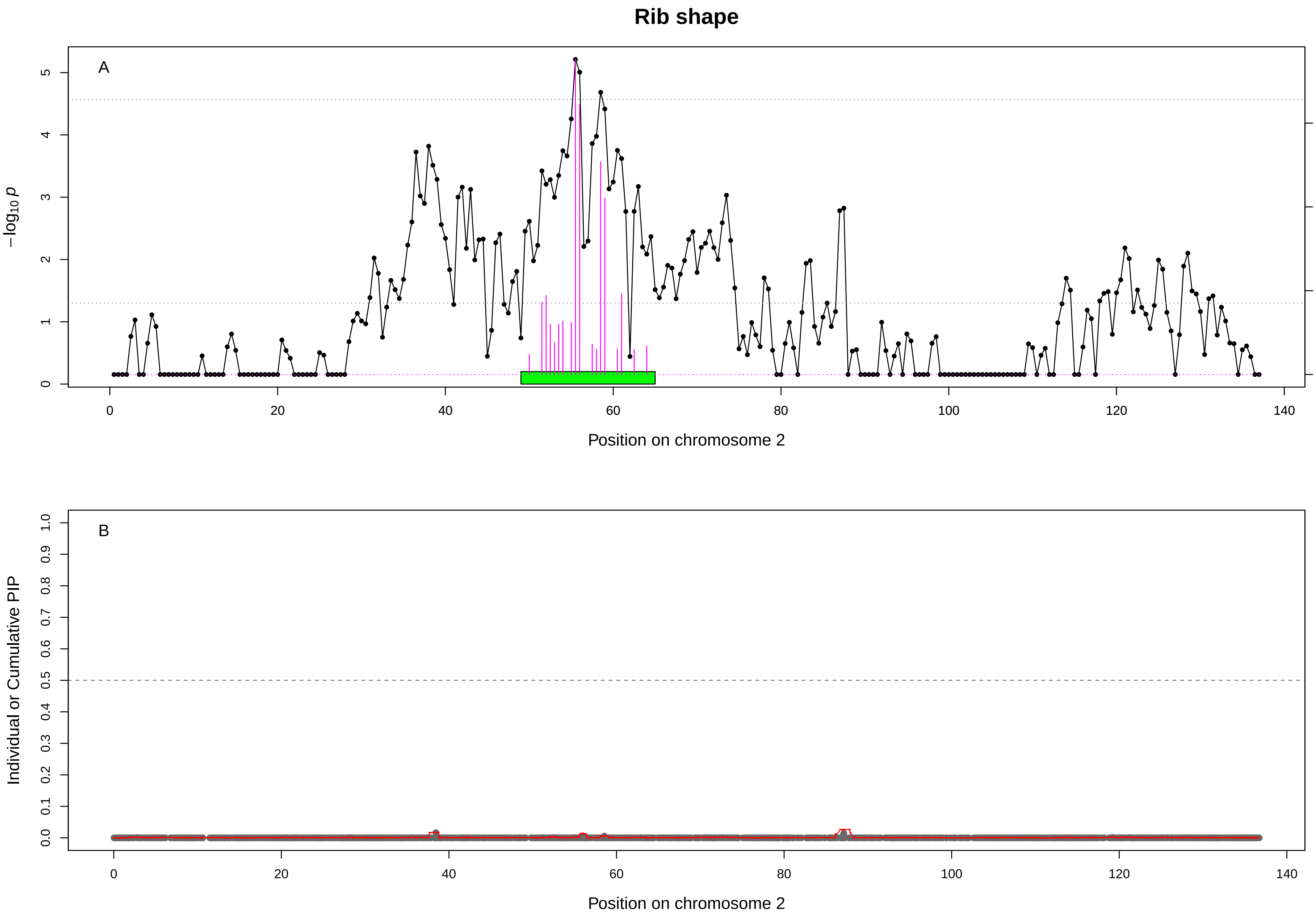

Rib shape
A
−log10 p
Position on chromosome 2
B
Individual or Cumulative PIP
Position on chromosome 2

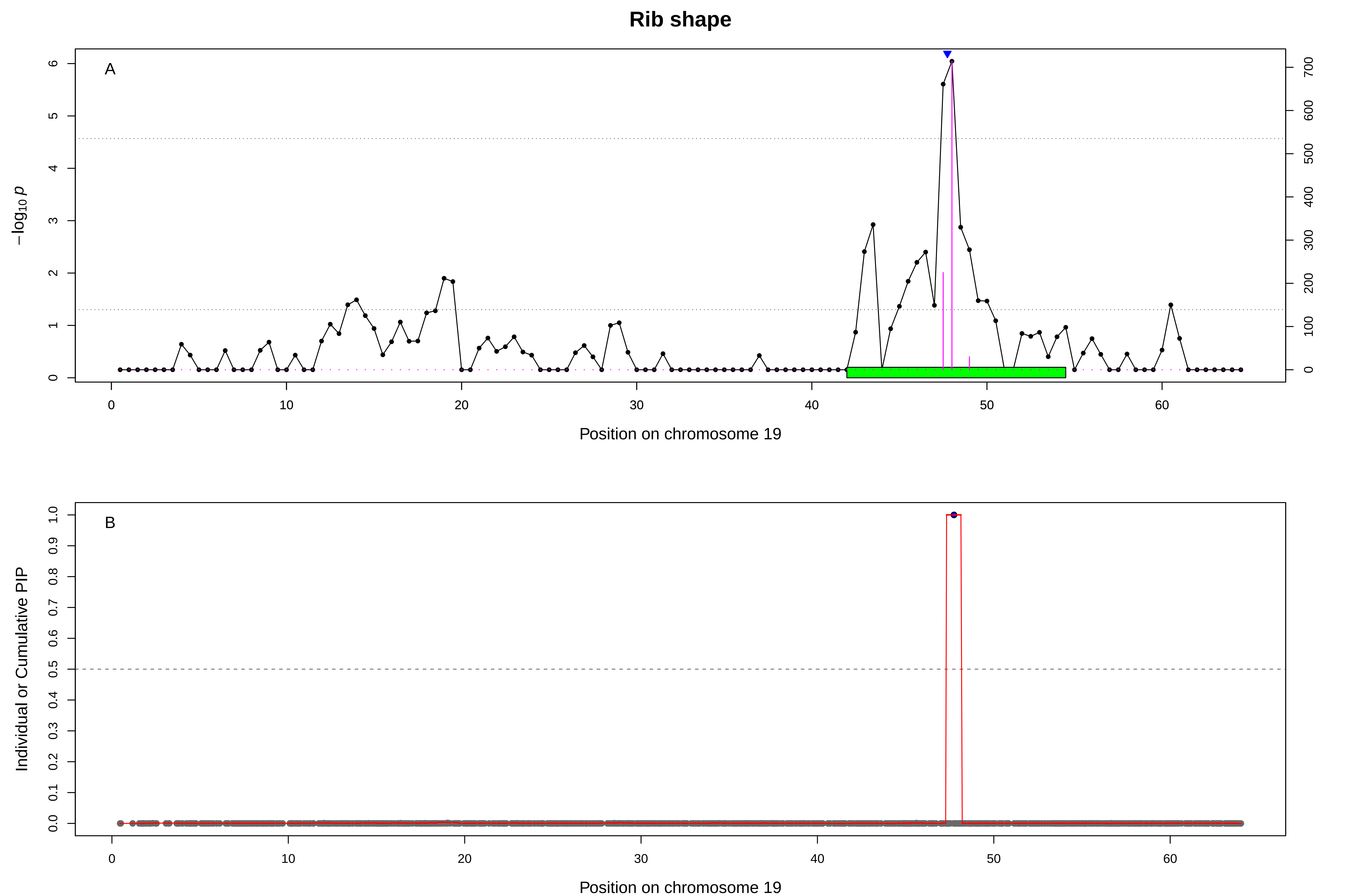

Rib shape
A
−log10 p
Position on chromosome 19
B
Individual or Cumulative PIP
Position on chromosome 19

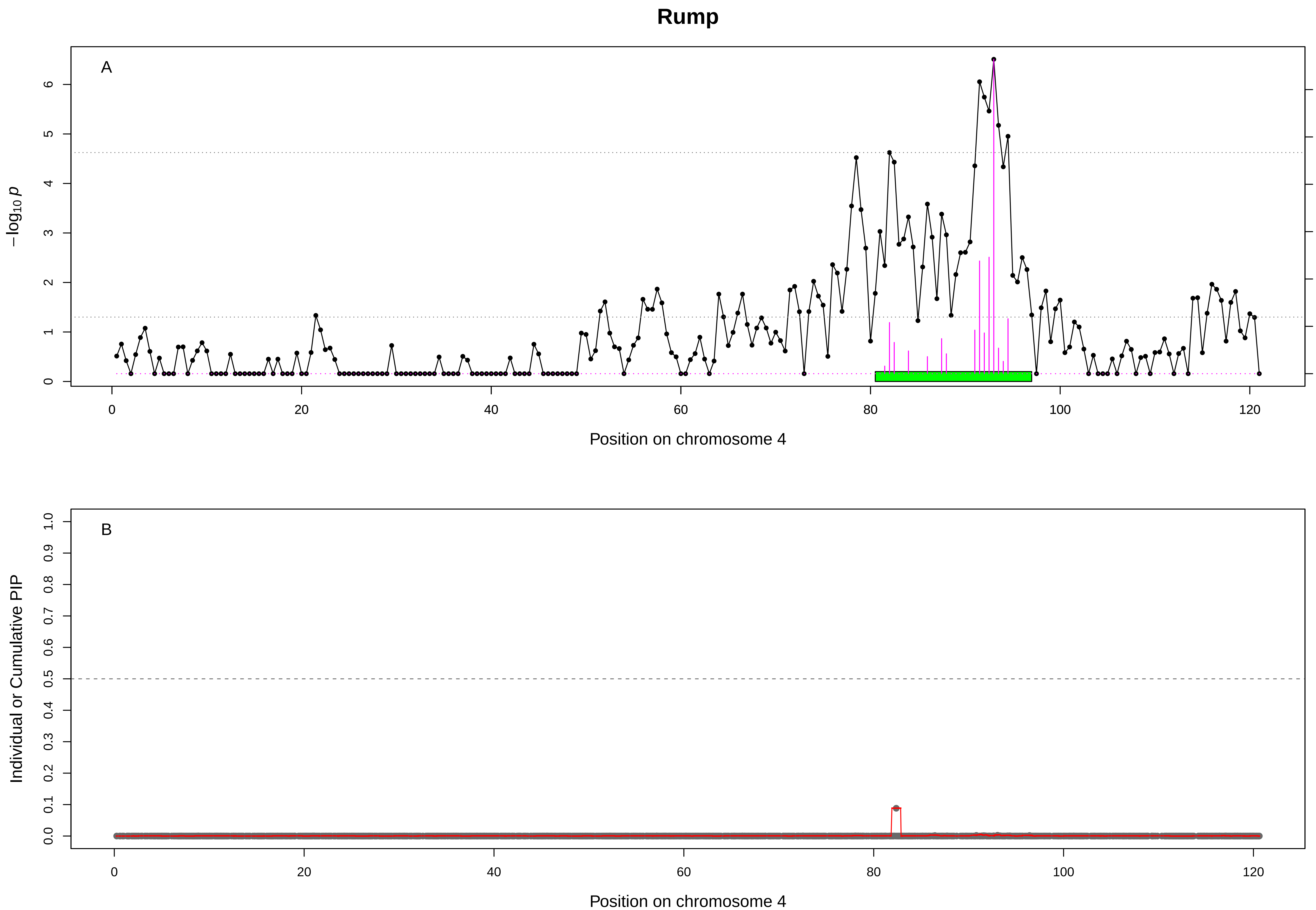

Rump
A
$-\log_{10} p$
0
1
2
3
4
5
6
0
50
100
150
200
250
300
0
20
40
60
80
100
120
Position on chromosome 4
B
Individual or Cumulative PIP
0.0
0.1
0.2
0.3
0.4
0.5
0.6
0.7
0.8
0.9
1.0
0
20
40
60
80
100
120
Position on chromosome 4

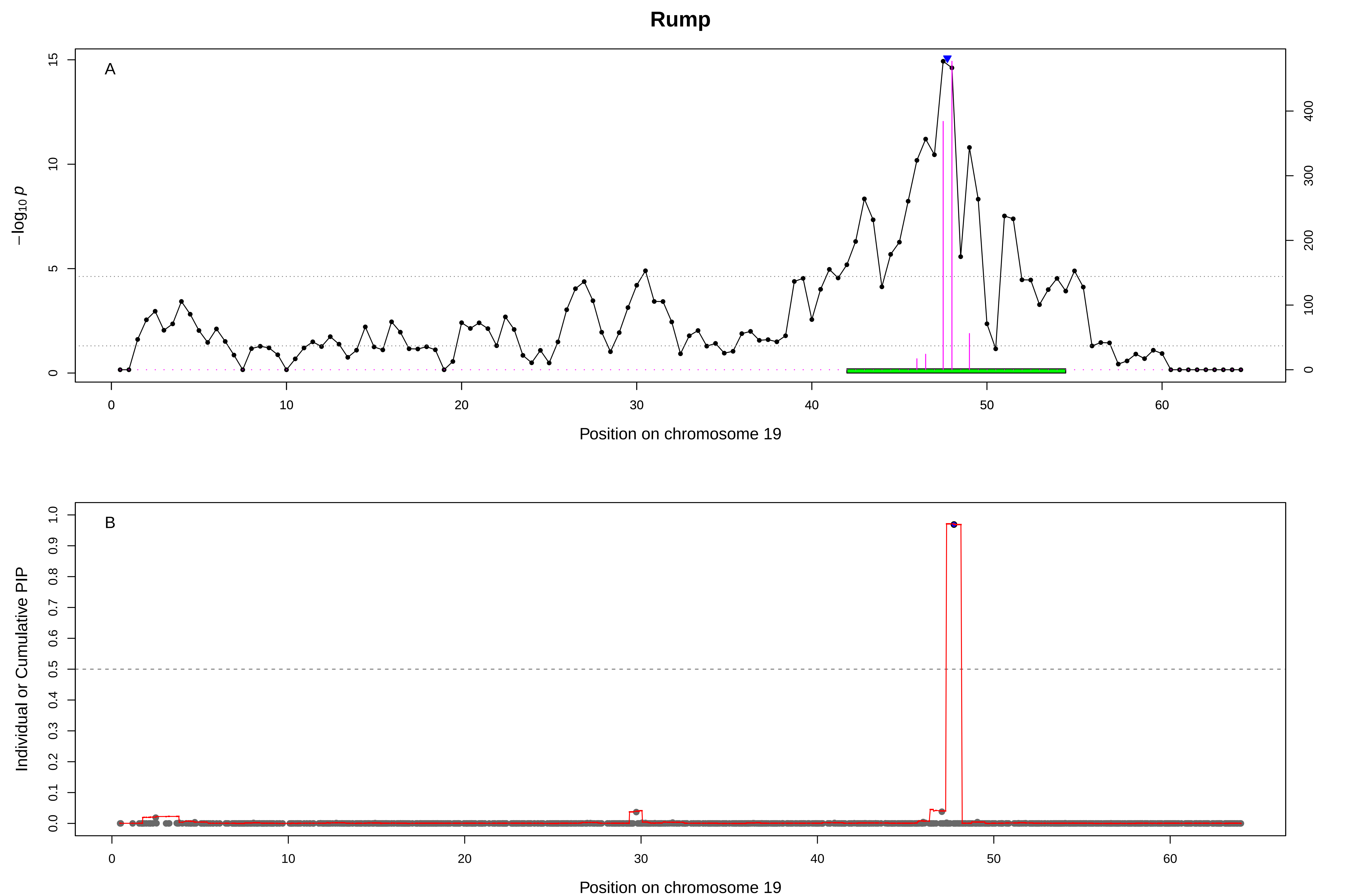

Rump
A
−log10 p
Position on chromosome 19
B
Individual or Cumulative PIP
Position on chromosome 19

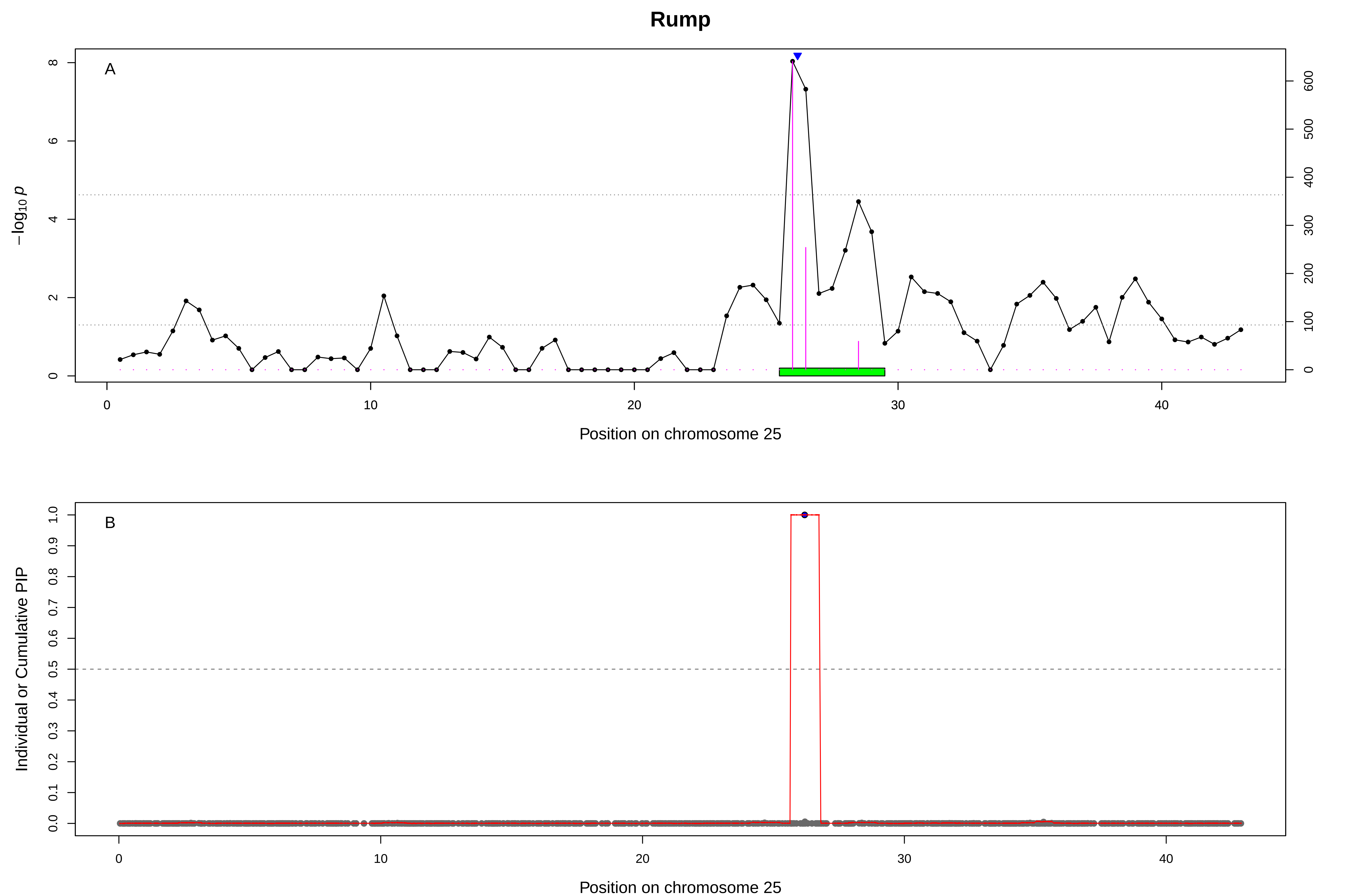
Rump
A
−log10 p
Position on chromosome 25
B
Individual or Cumulative PIP
Position on chromosome 25

Shoulder muscling

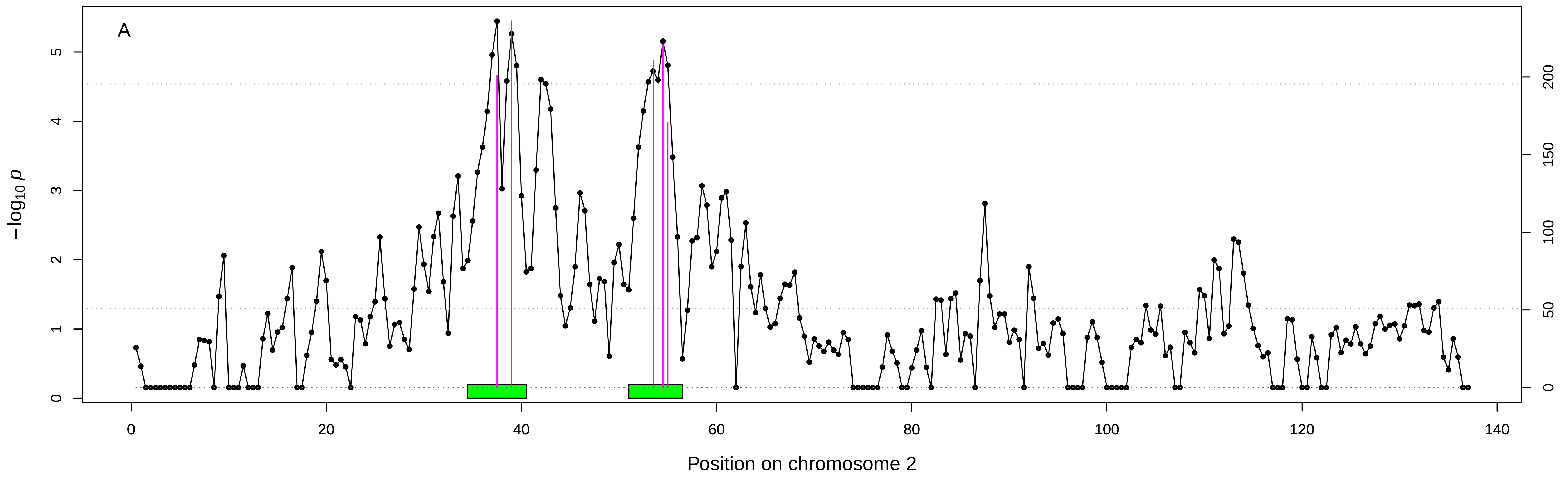

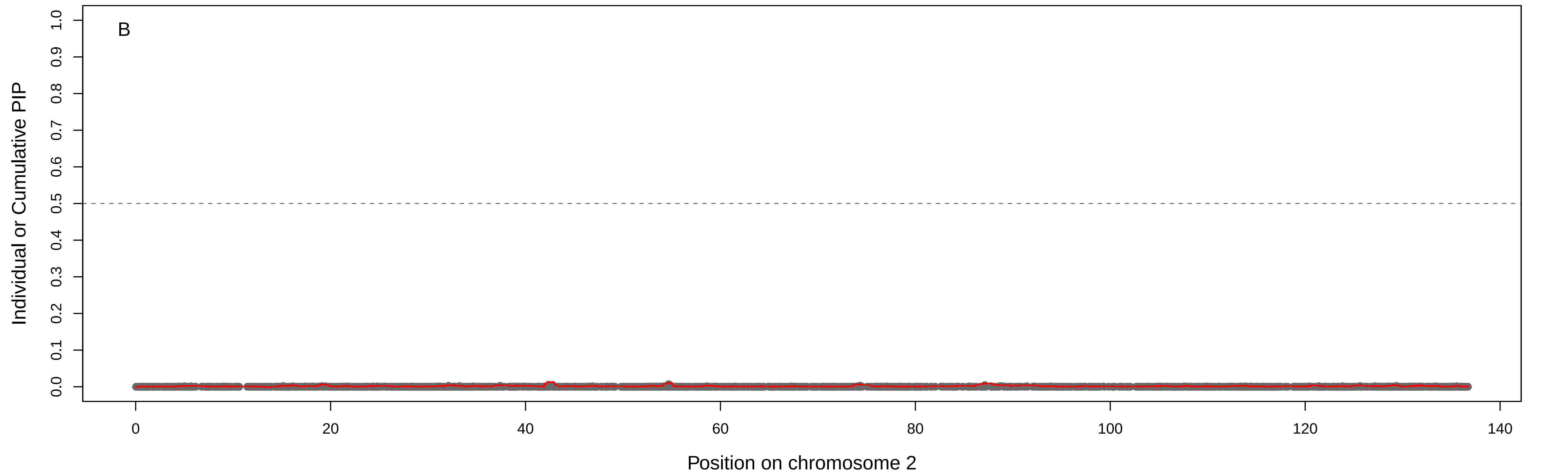

Shoulder muscling

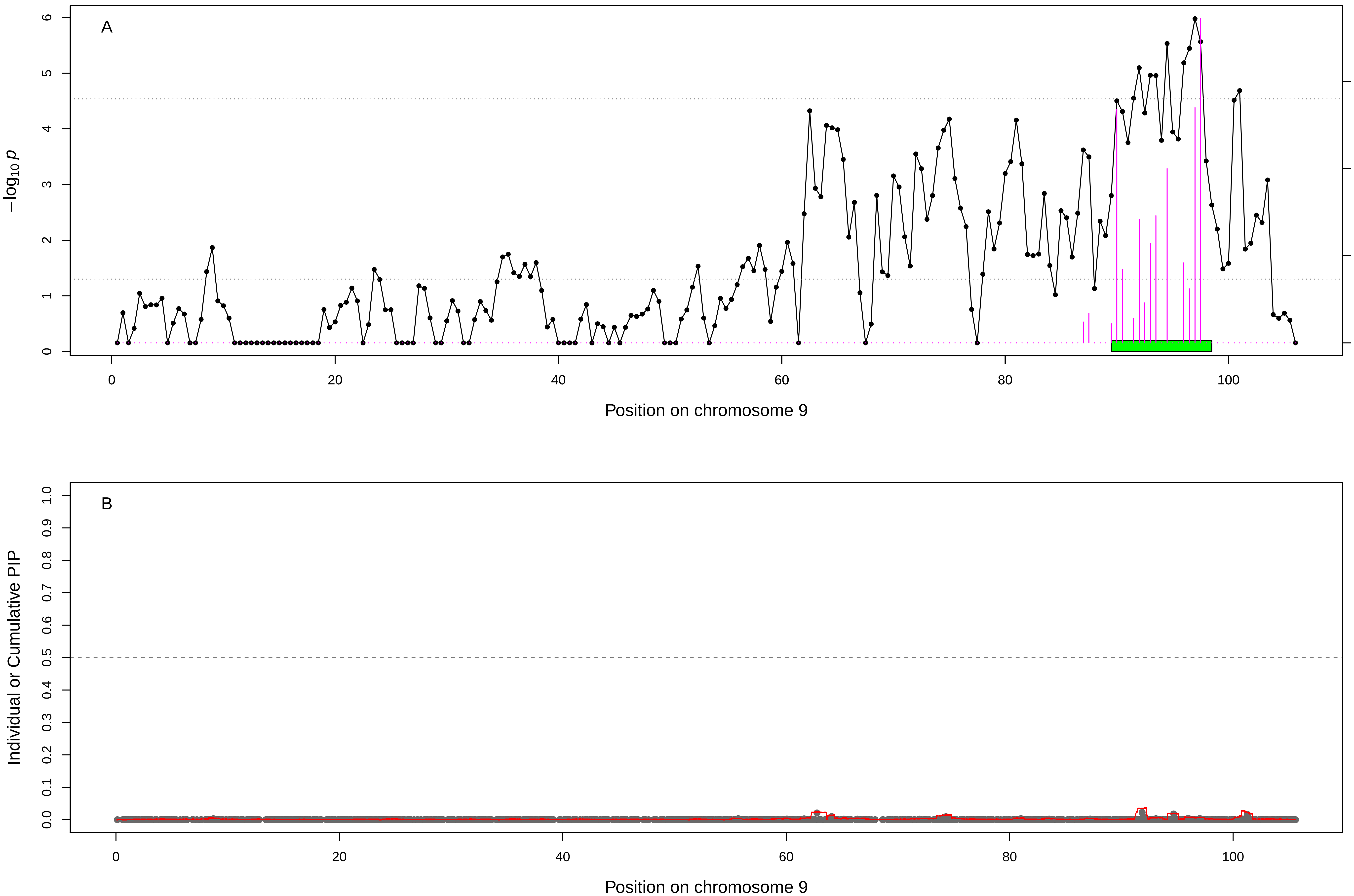

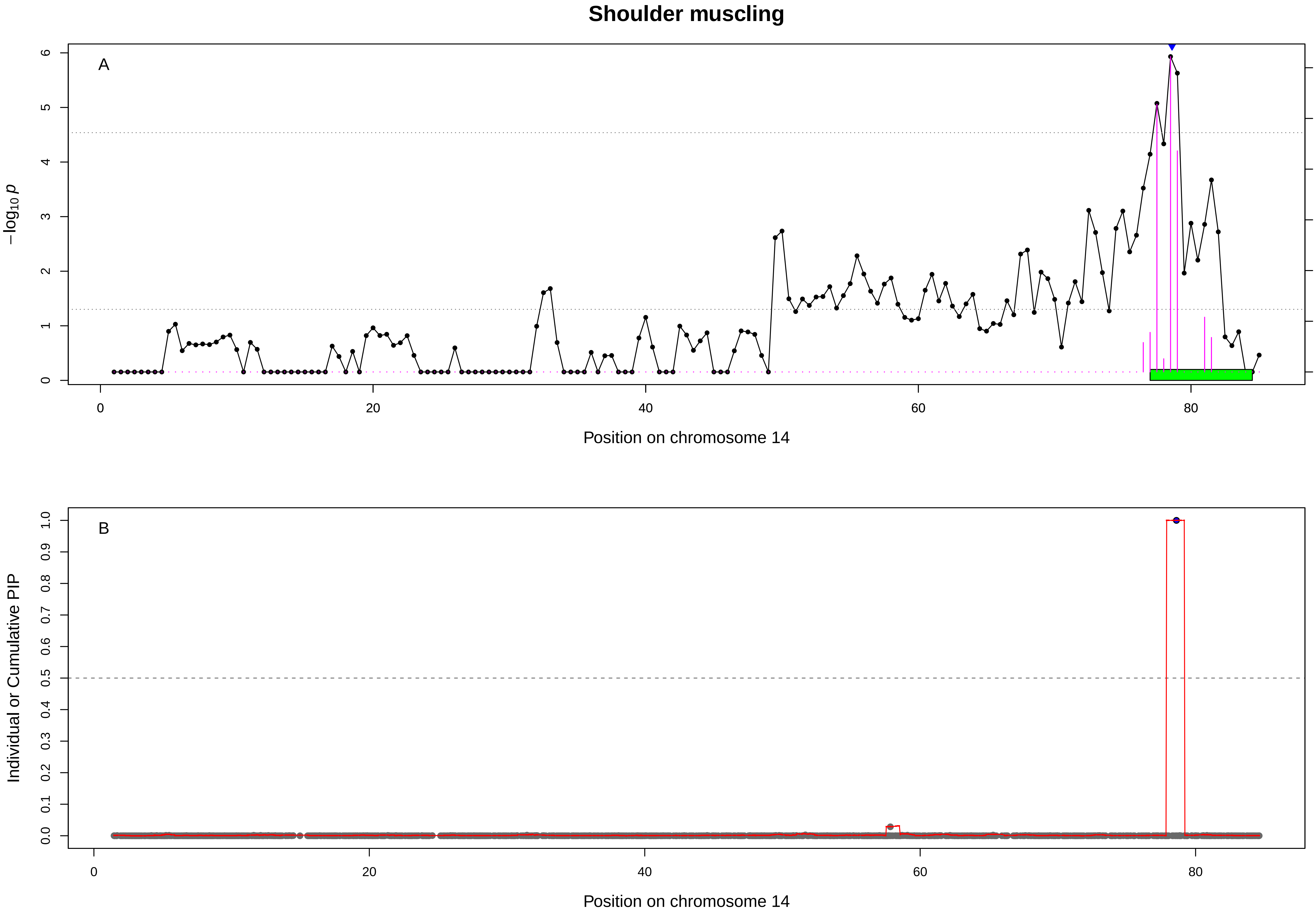
Shoulder muscling
A
$-\log_{10} p$
Position on chromosome 14
B
Individual or Cumulative PIP
Position on chromosome 14

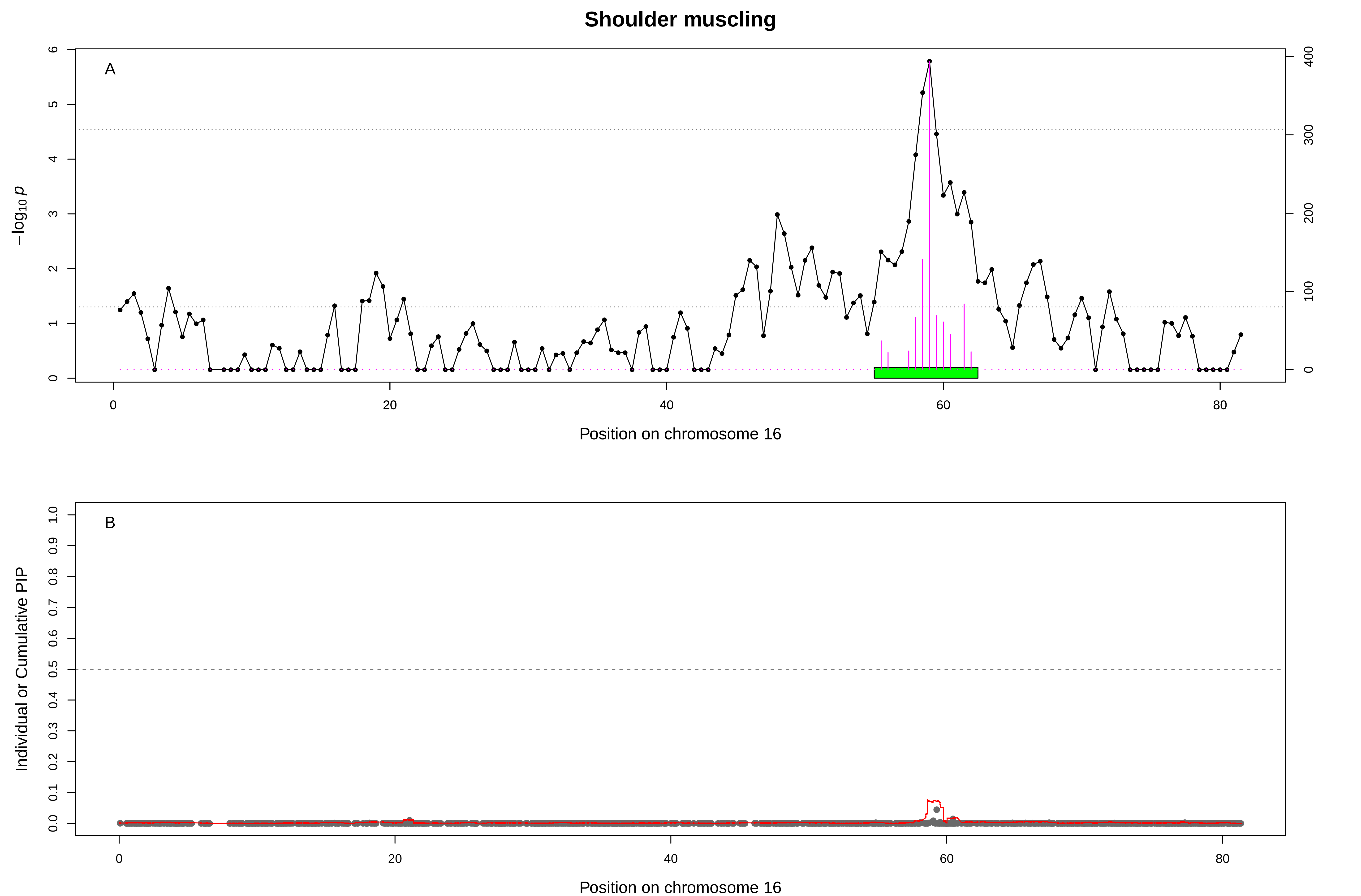

Shoulder muscling
A
$-\log_{10} p$
Position on chromosome 16
B
Individual or Cumulative PIP
Position on chromosome 16

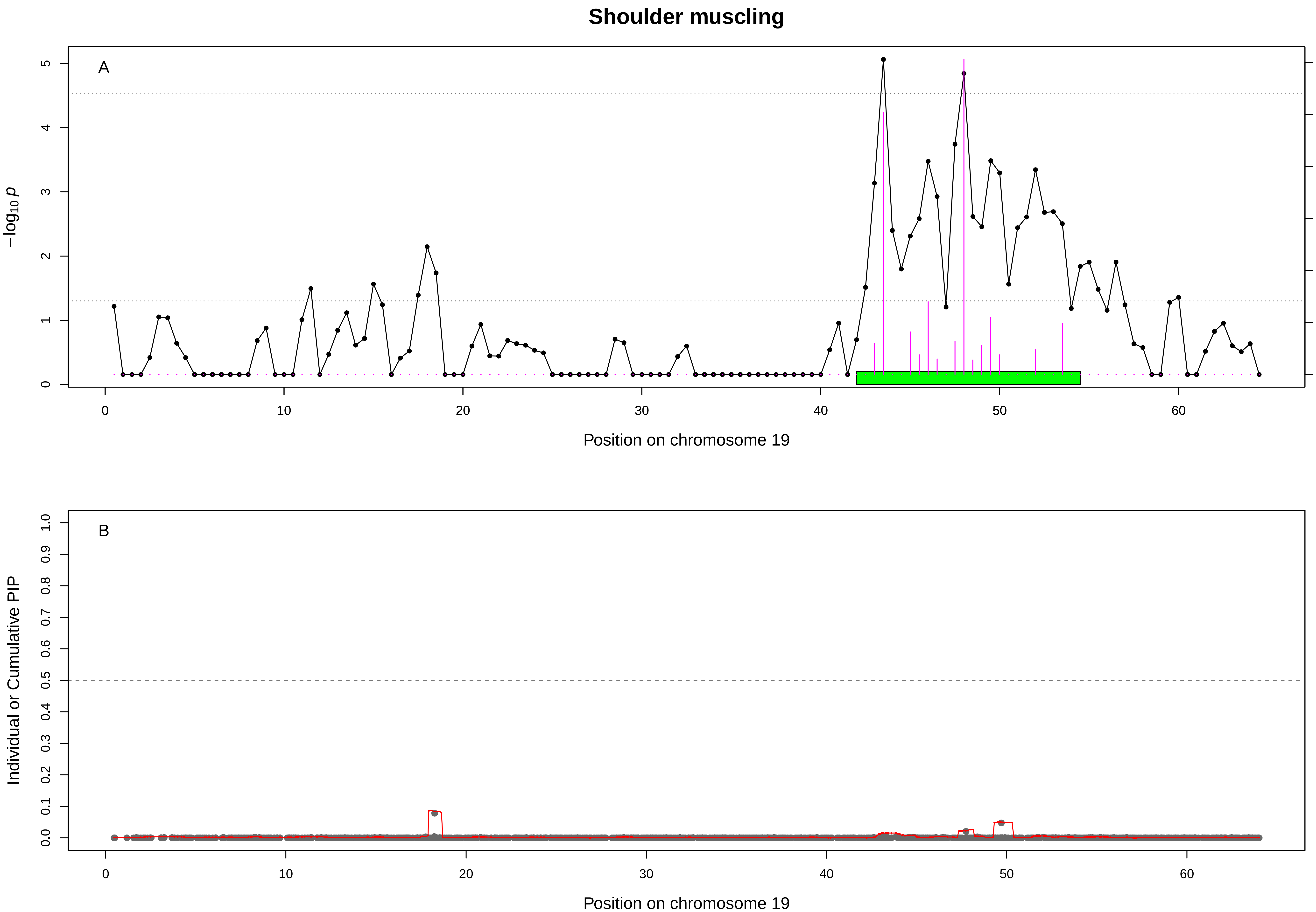

Shoulder muscling
A
−log10 p
Position on chromosome 19
B
Individual or Cumulative PIP
Position on chromosome 19

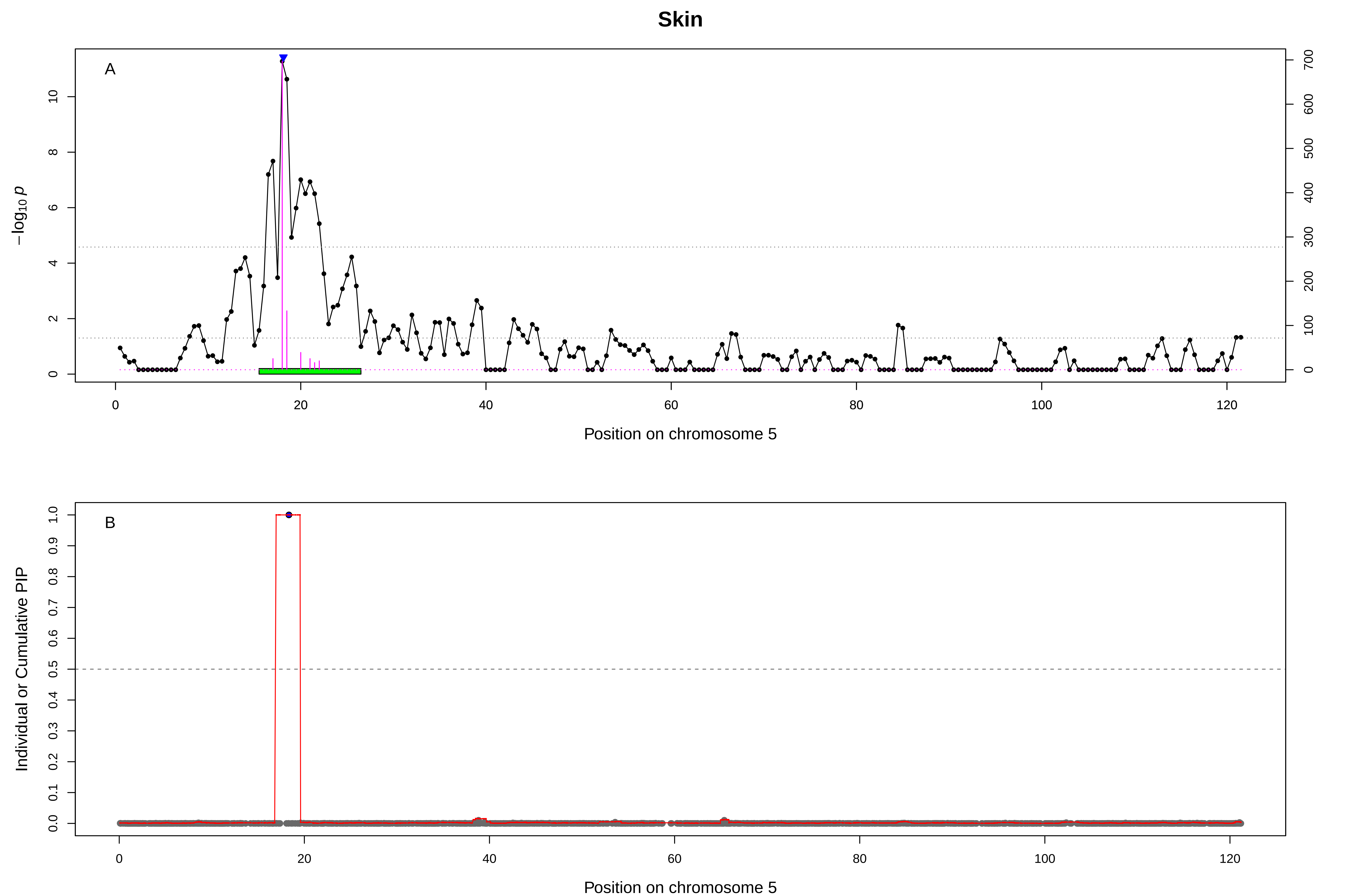

Skin
A
−log10 p
Position on chromosome 5
B
Individual or Cumulative PIP
Position on chromosome 5

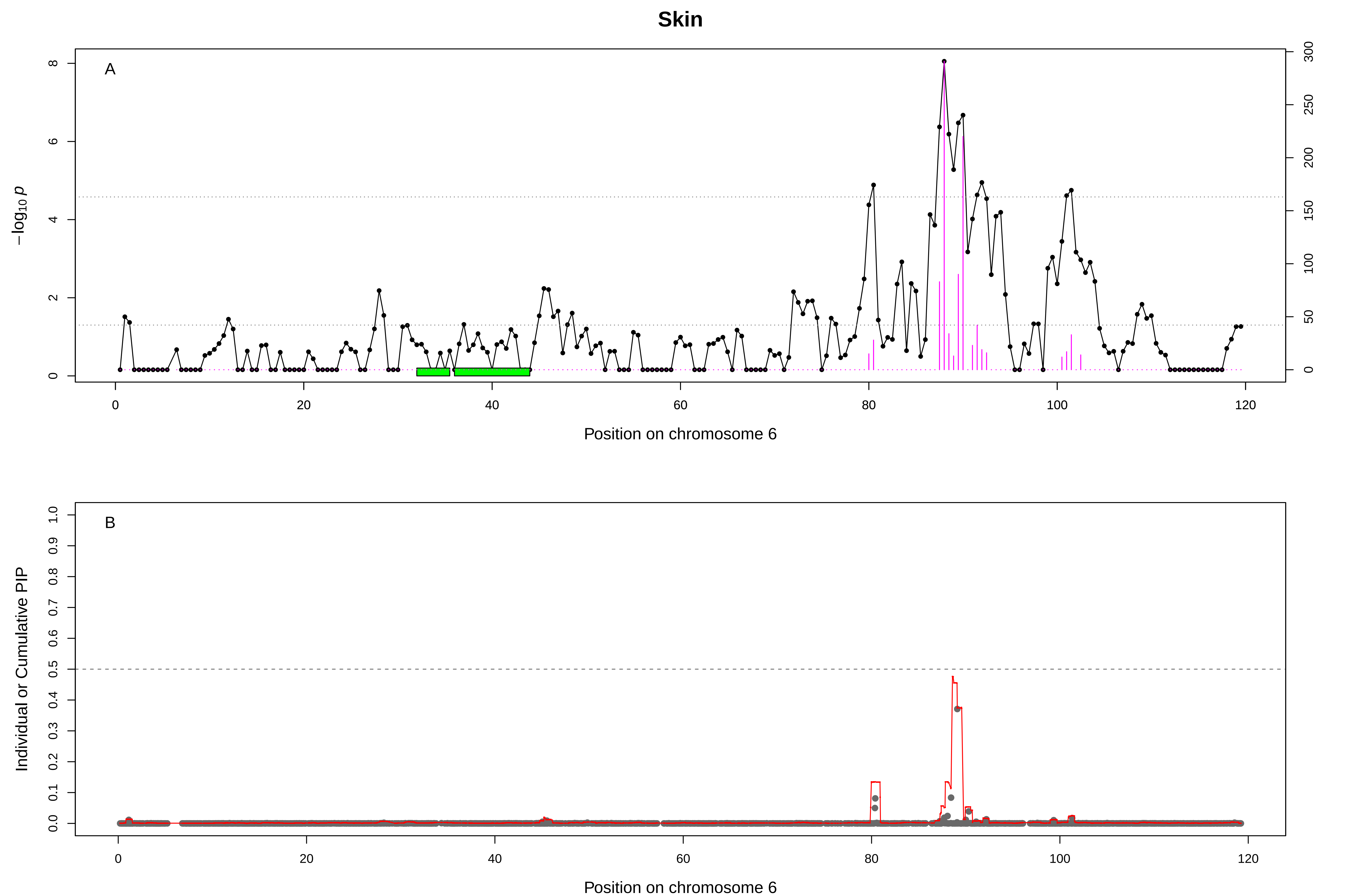
Skin
A
−log10 p
Position on chromosome 6
B
Individual or Cumulative PIP
Position on chromosome 6

Tail set

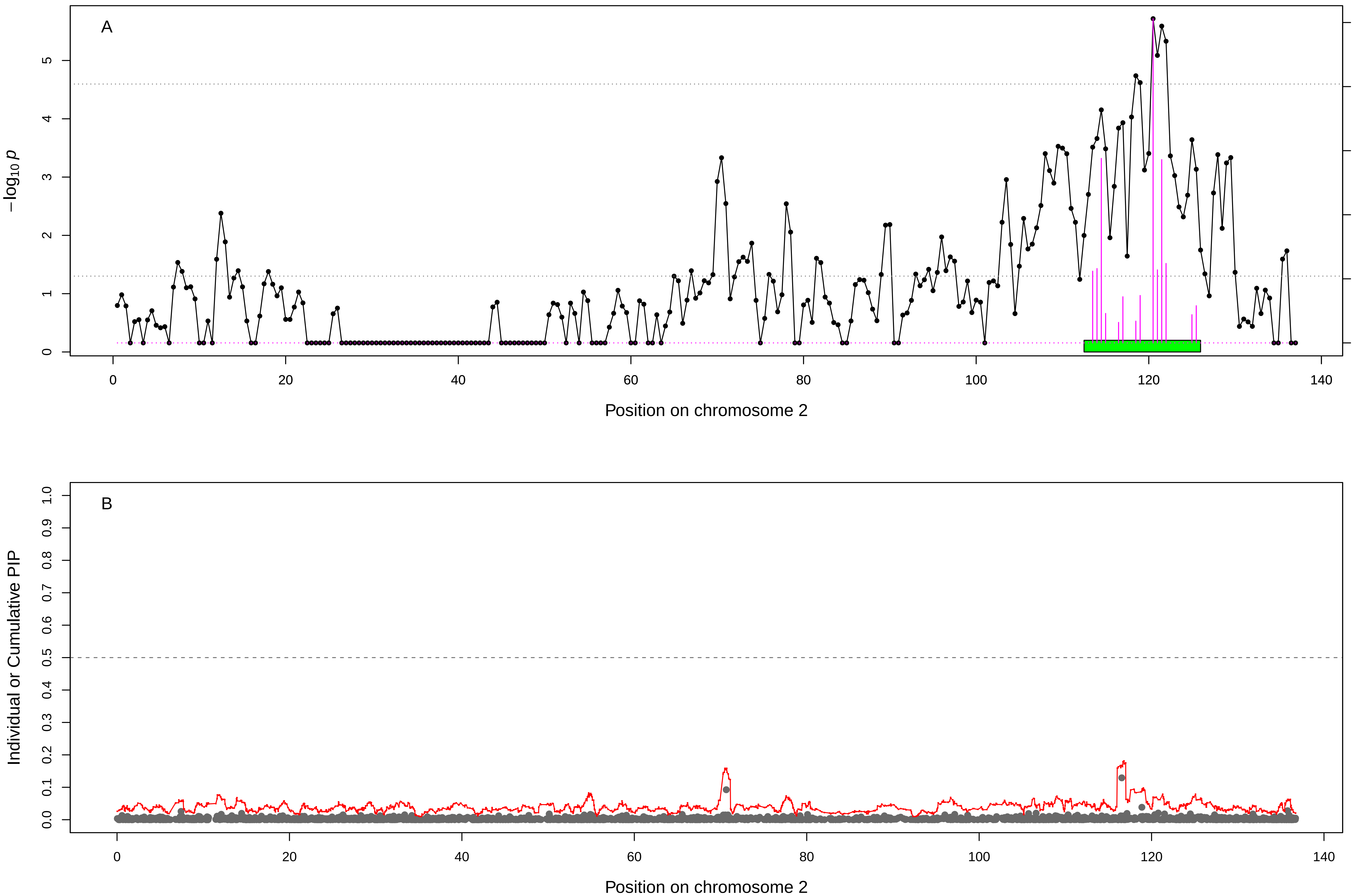
A
B
−log10 p
Individual or Cumulative PIP
Position on chromosome 2
Position on chromosome 2

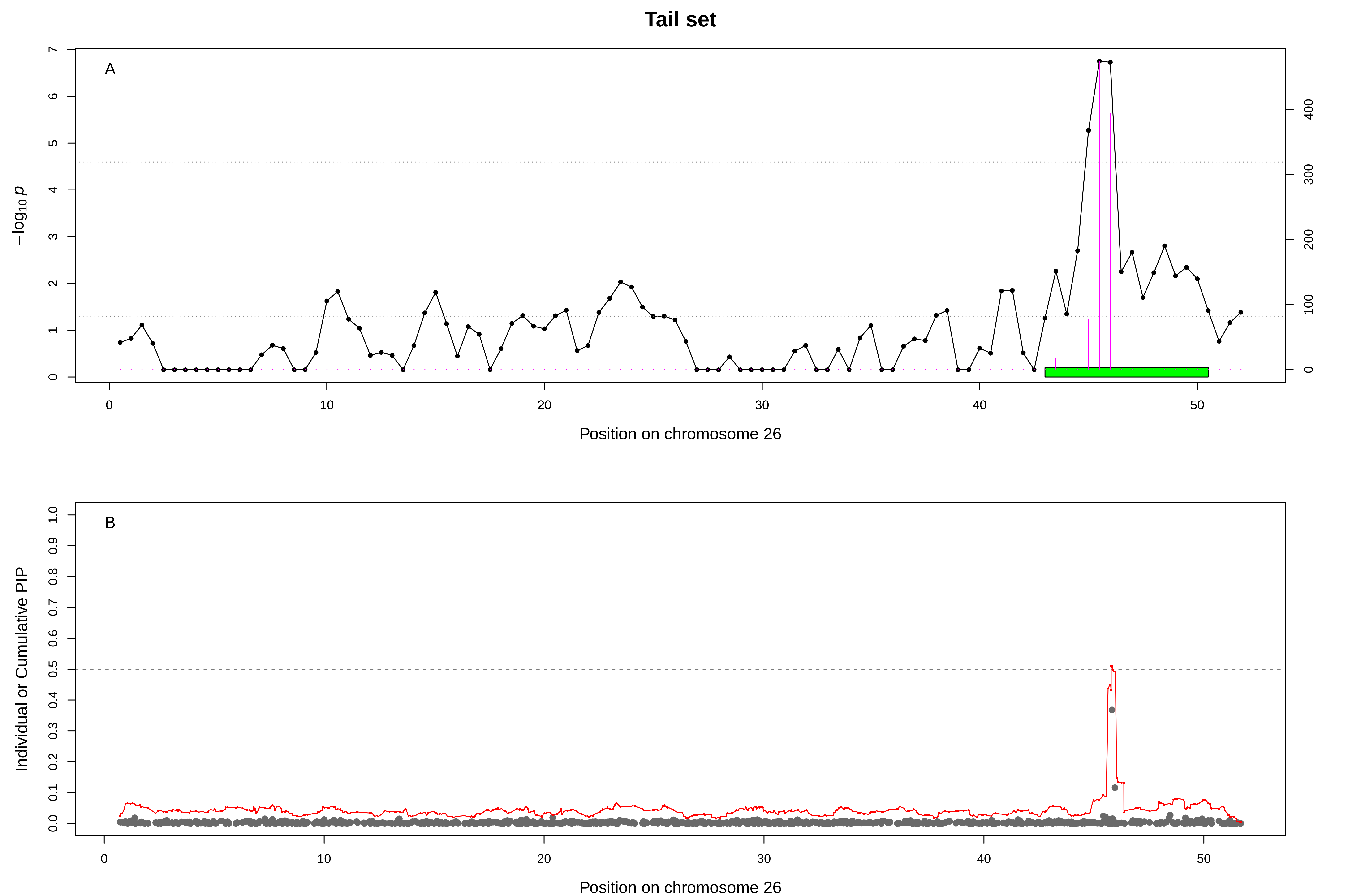

Tail set
A
−log10 p
Position on chromosome 26
B
Individual or Cumulative PIP
Position on chromosome 26

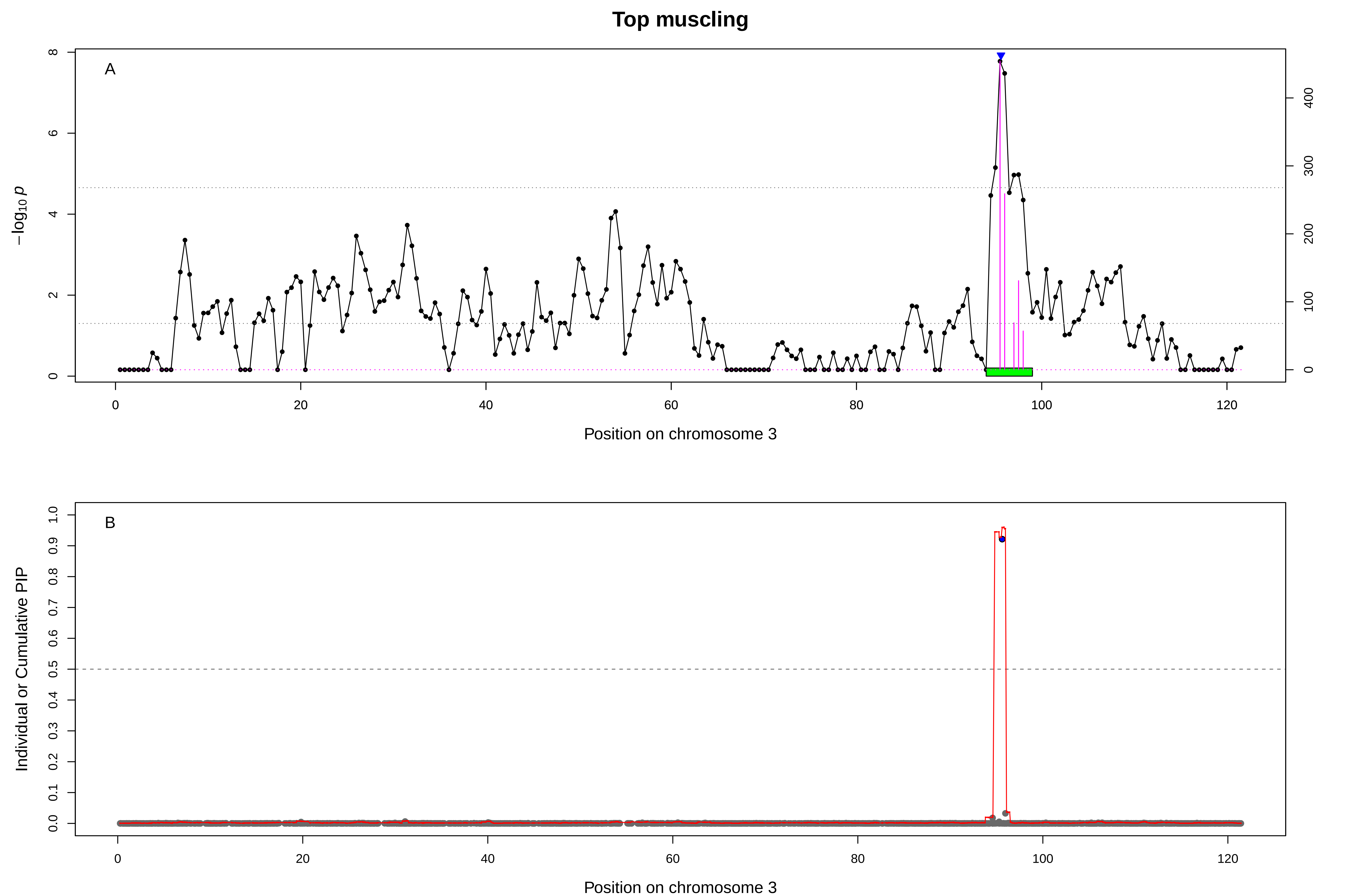

Top muscling
A
$-\log_{10} p$
Position on chromosome 3
B
Individual or Cumulative PIP
Position on chromosome 3

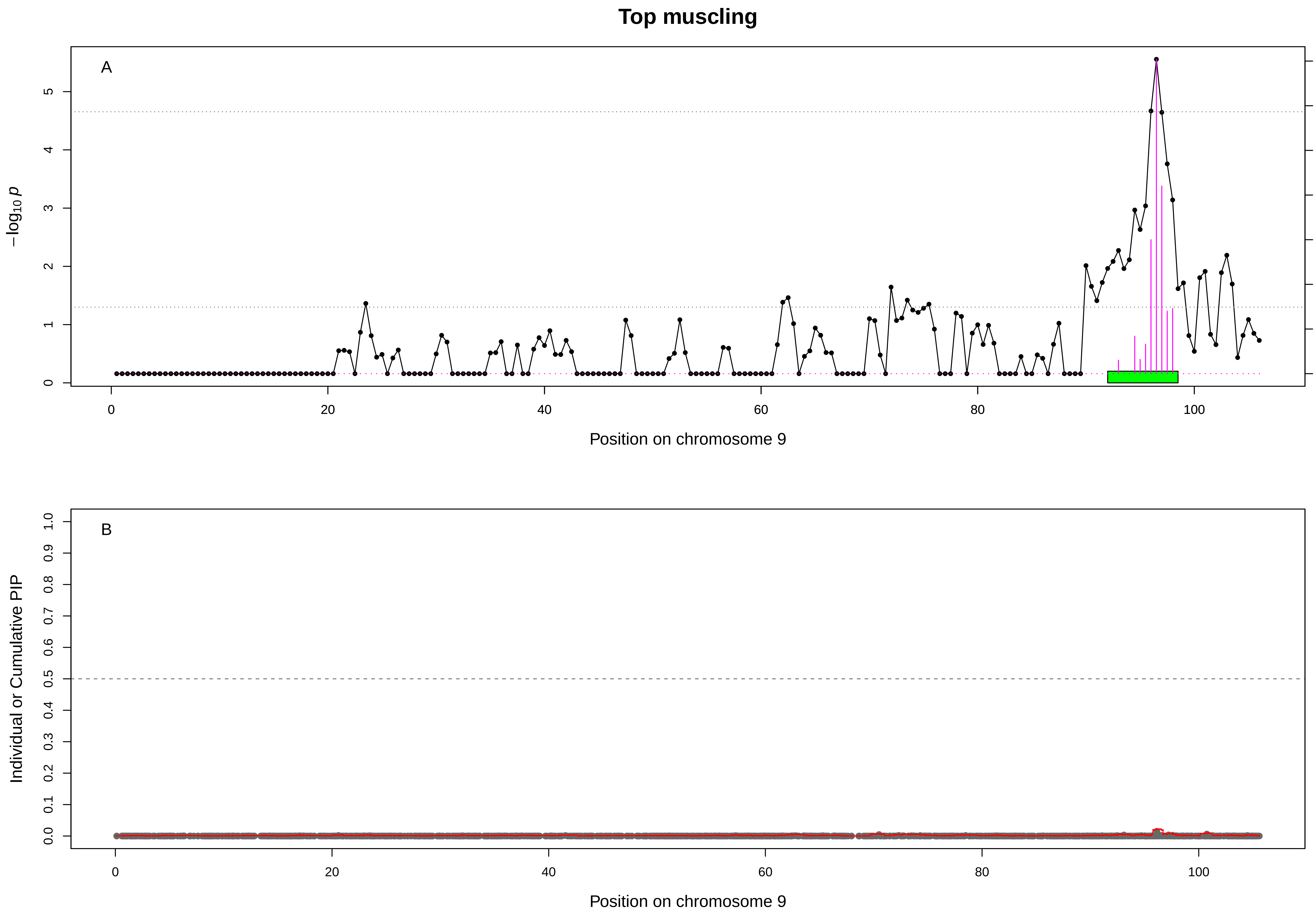

Top muscling
A
B
−log10 p
Individual or Cumulative PIP
Position on chromosome 9
Position on chromosome 9

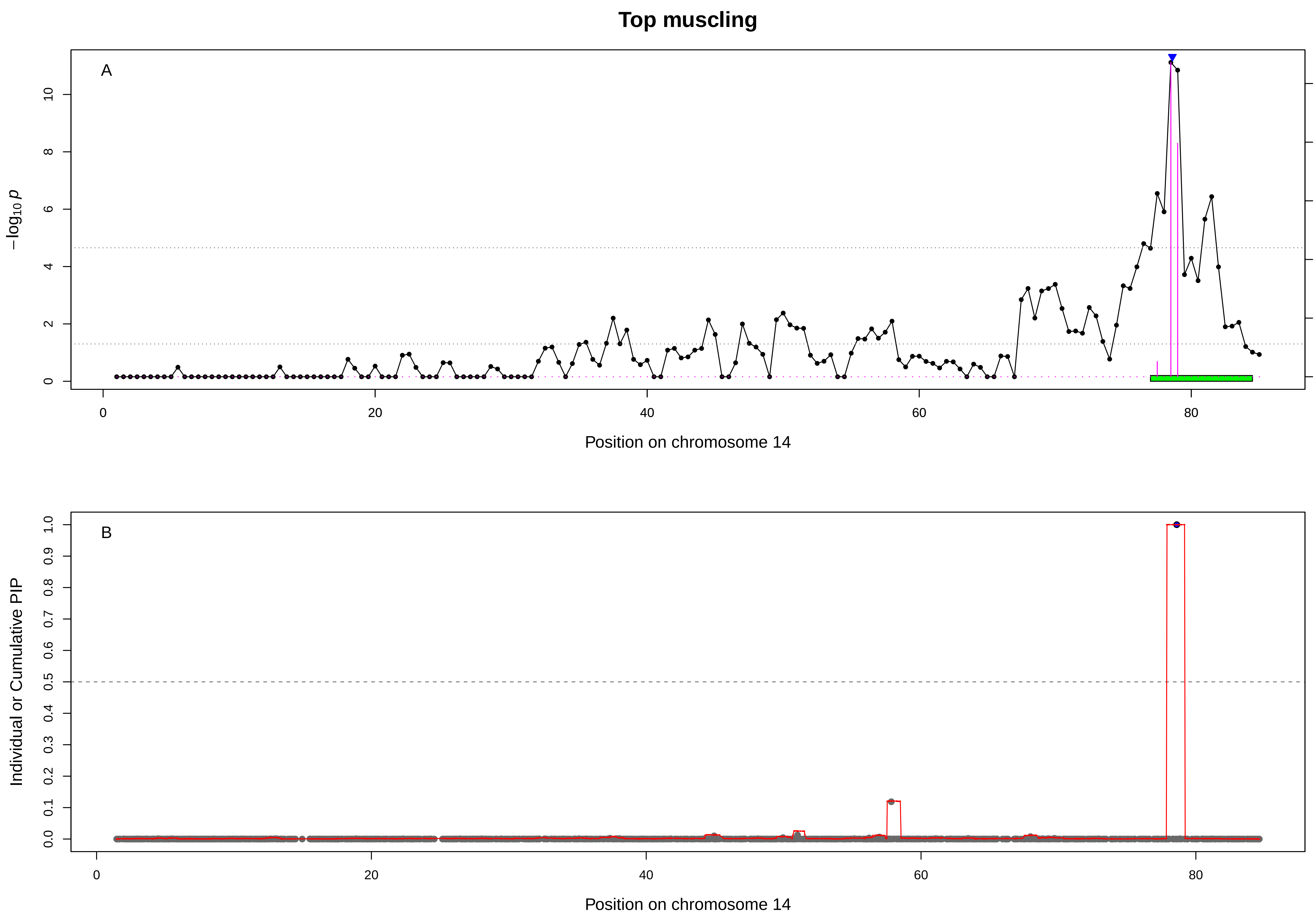

Top muscling
A
−log10 p
Position on chromosome 14
B
Individual or Cumulative PIP
Position on chromosome 14

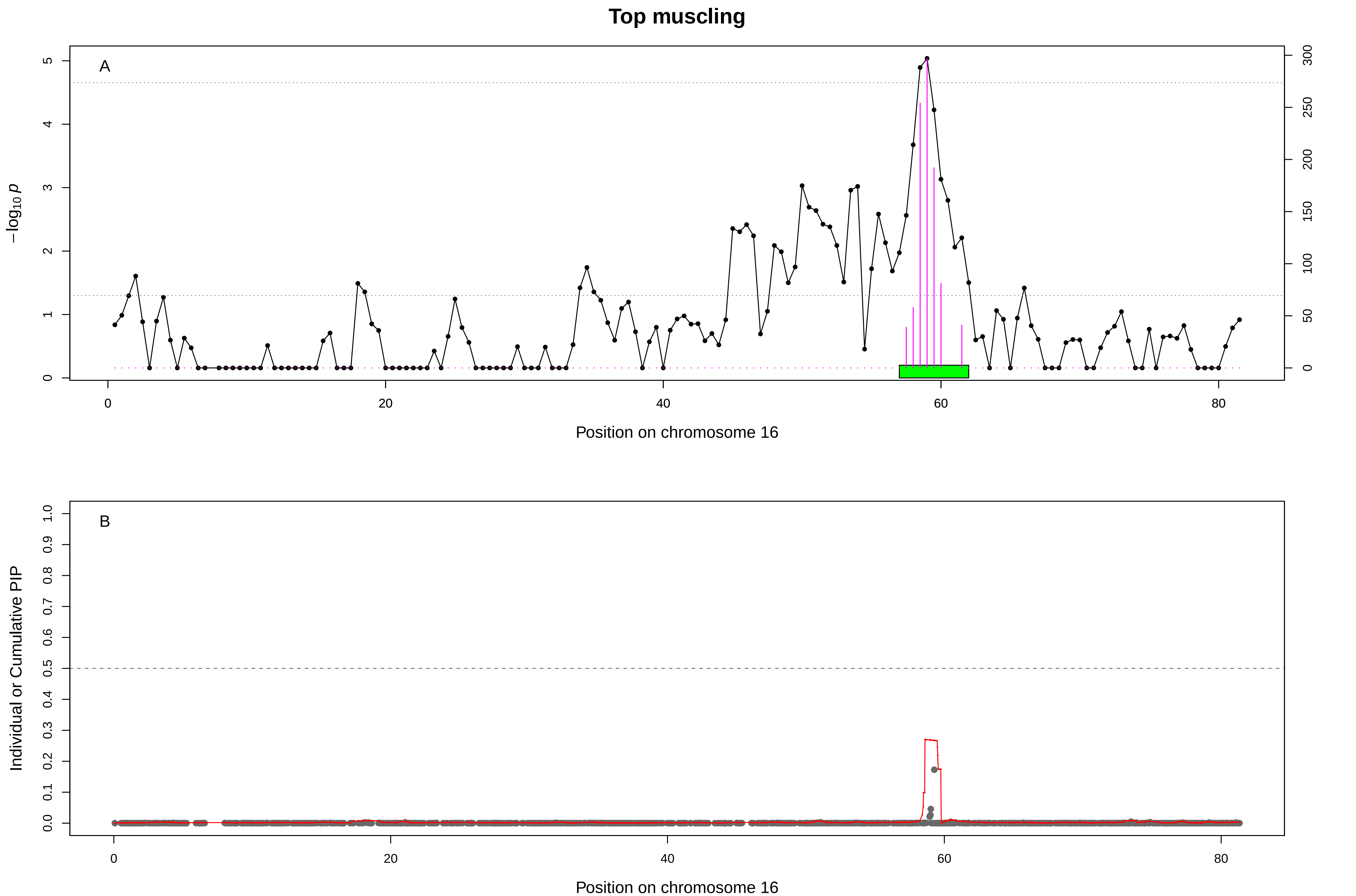

Top muscling
A
$-\log_{10} p$
Position on chromosome 16
0
20
40
60
80
0
1
2
3
4
5
0
50
100
150
200
250
300
B
Individual or Cumulative PIP
0.0
0.1
0.2
0.3
0.4
0.5
0.6
0.7
0.8
0.9
1.0
Position on chromosome 16

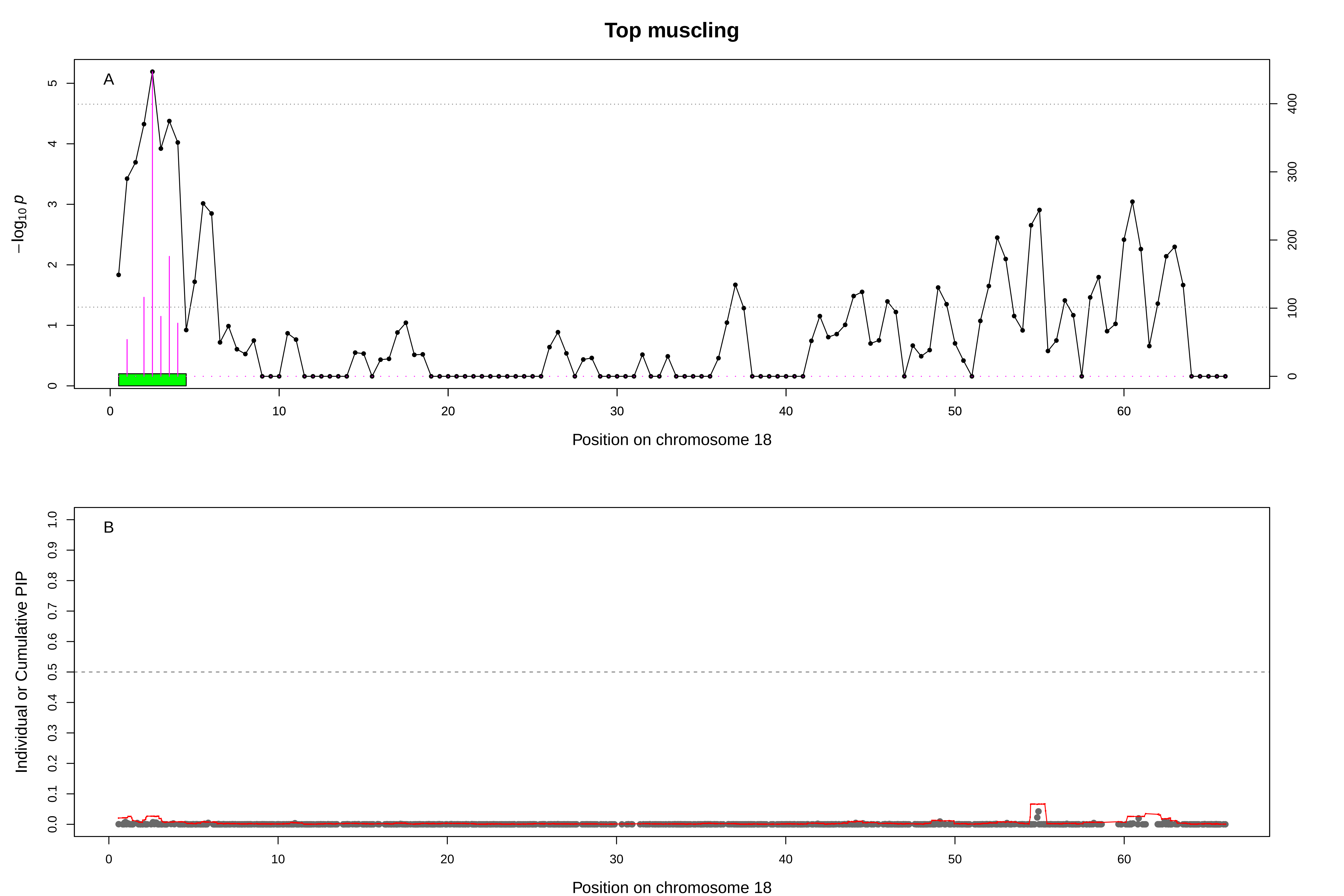

Top muscling
A
−log10 p
Position on chromosome 18
B
Individual or Cumulative PIP
Position on chromosome 18

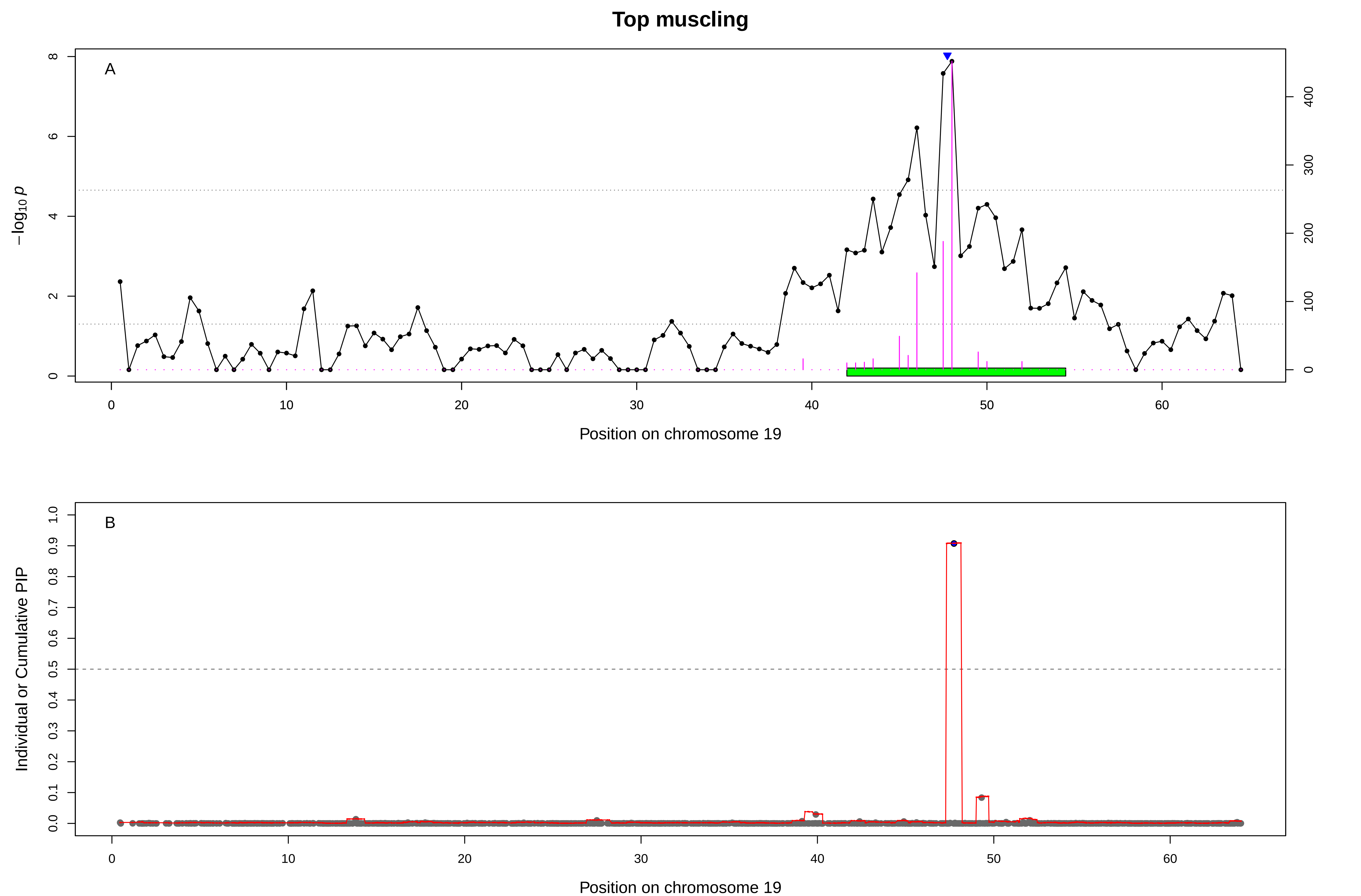

Top muscling
A
−log10 p
Position on chromosome 19
B
Individual or Cumulative PIP
Position on chromosome 19

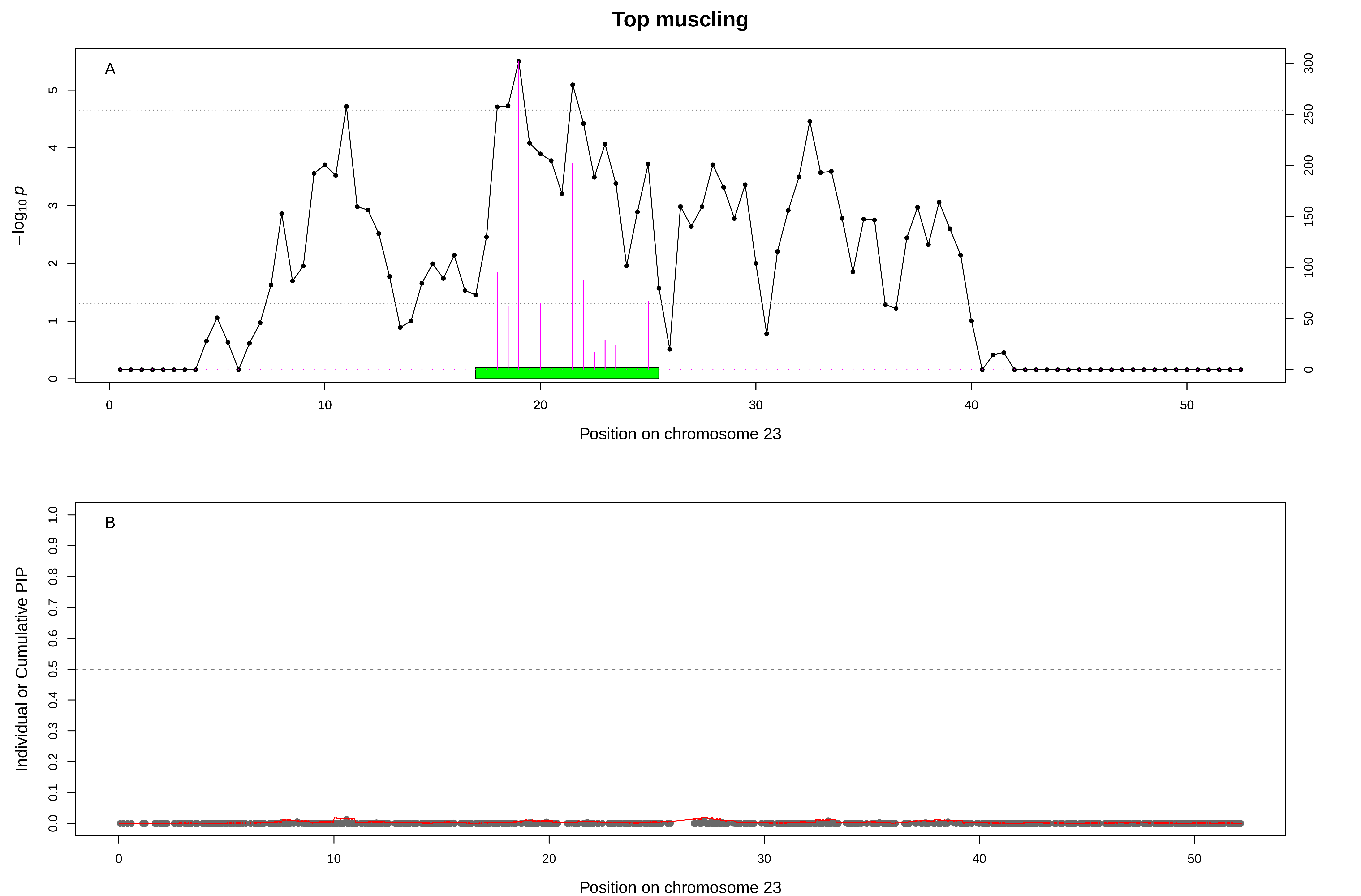

Top muscling
A
B
$-\log_{10} p$
Individual or Cumulative PIP
Position on chromosome 23
0 10 20 30 40 50
0 1 2 3 4 5
0 50 100 150 200 250 300
0.0 0.1 0.2 0.3 0.4 0.5 0.6 0.7 0.8 0.9 1.0

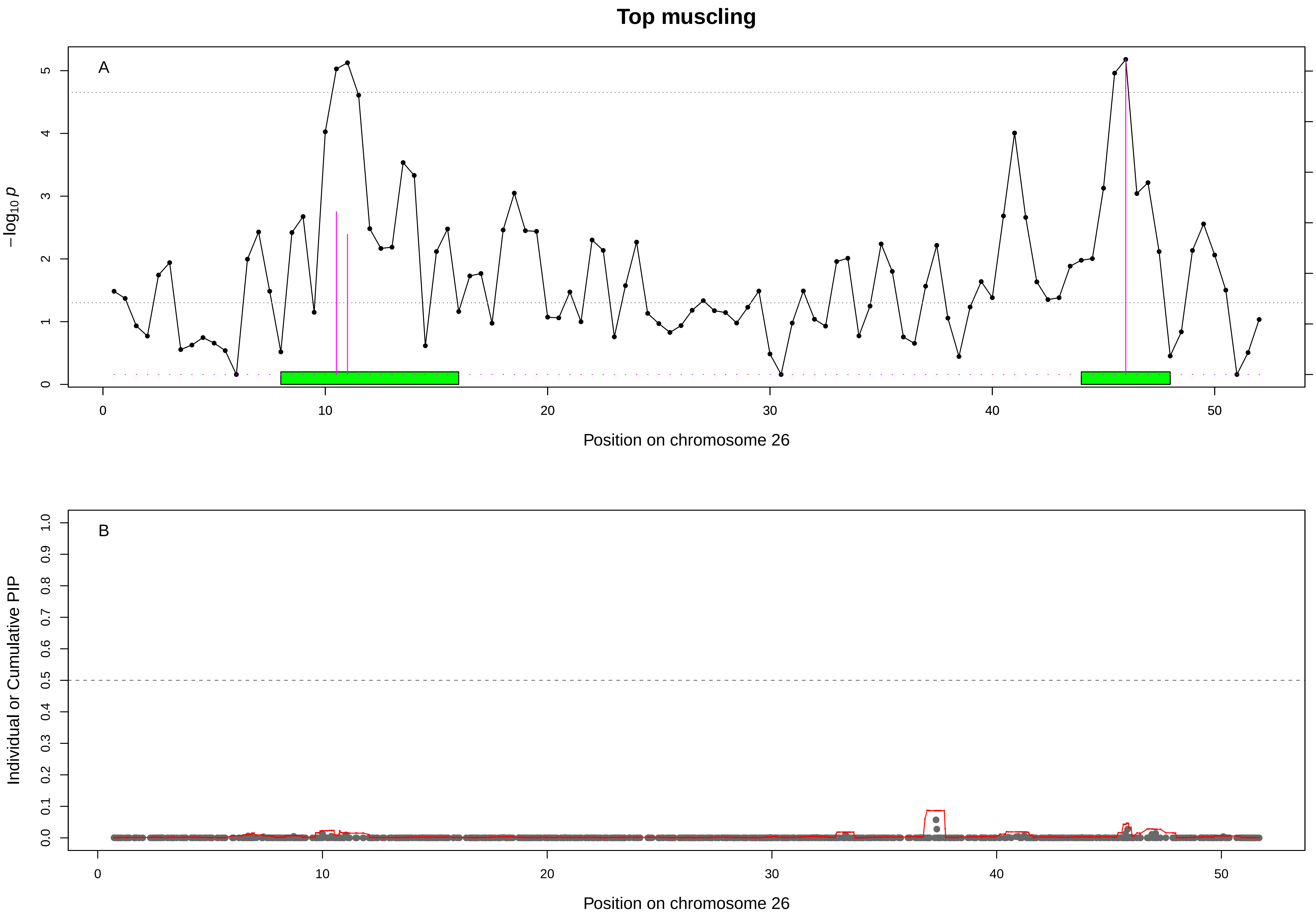
Top muscling
A
$-\log_{10} p$
Position on chromosome 26
B
Individual or Cumulative PIP
Position on chromosome 26

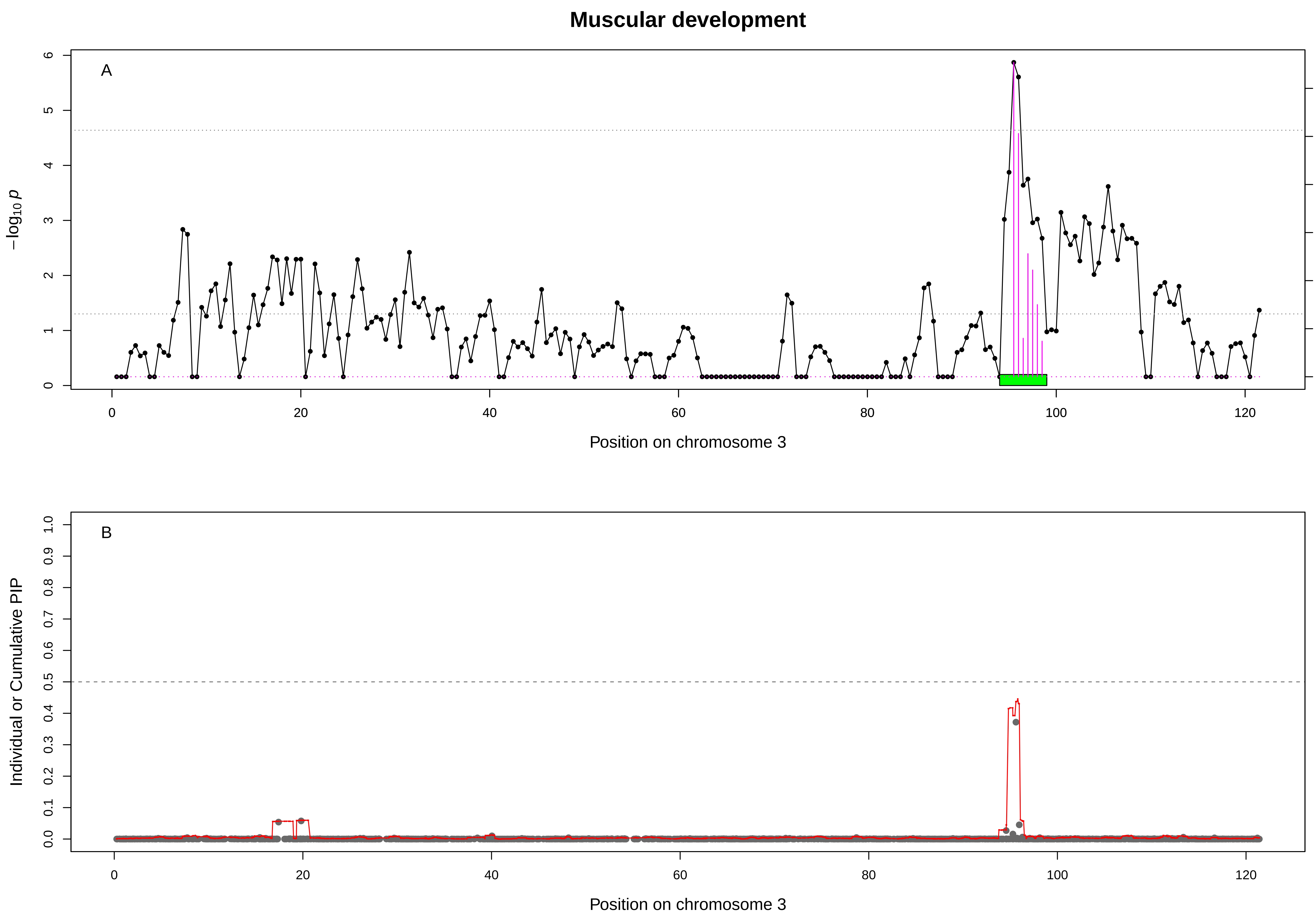
Muscular development
A
−log10 p
Position on chromosome 3
B
Individual or Cumulative PIP
Position on chromosome 3

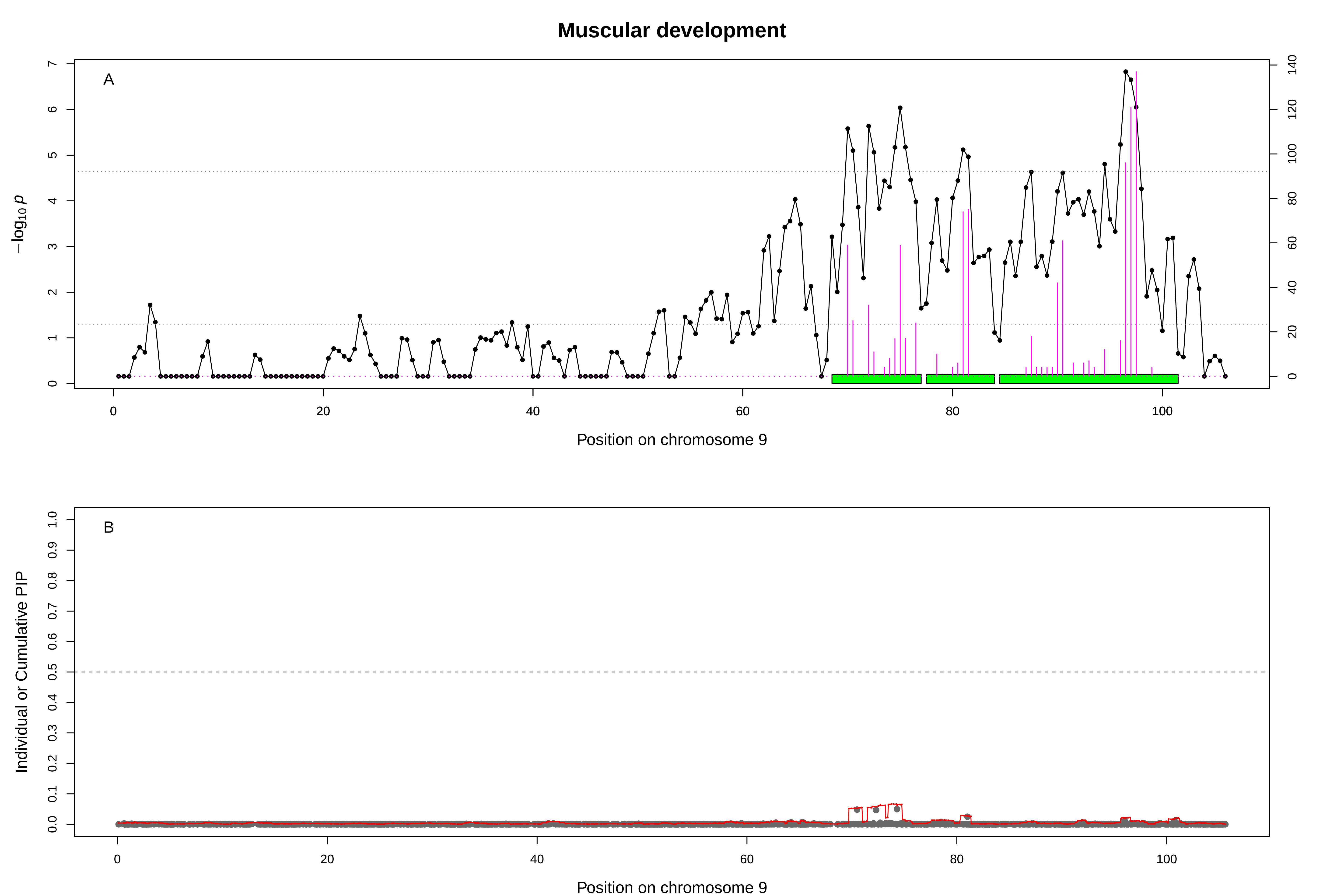

Muscular development
A
B
−log10 p
Individual or Cumulative PIP
Position on chromosome 9
Position on chromosome 9

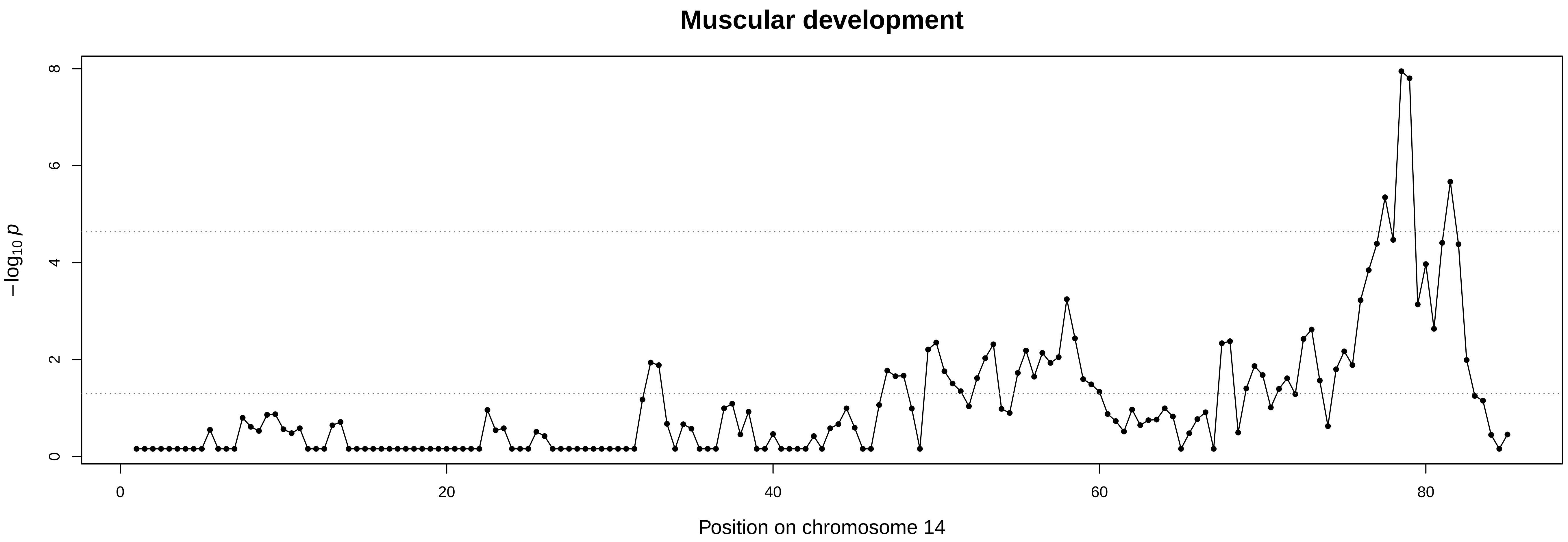

Muscular development
−log10 p
Position on chromosome 14
0
2
4
6
8
0
20
40
60
80

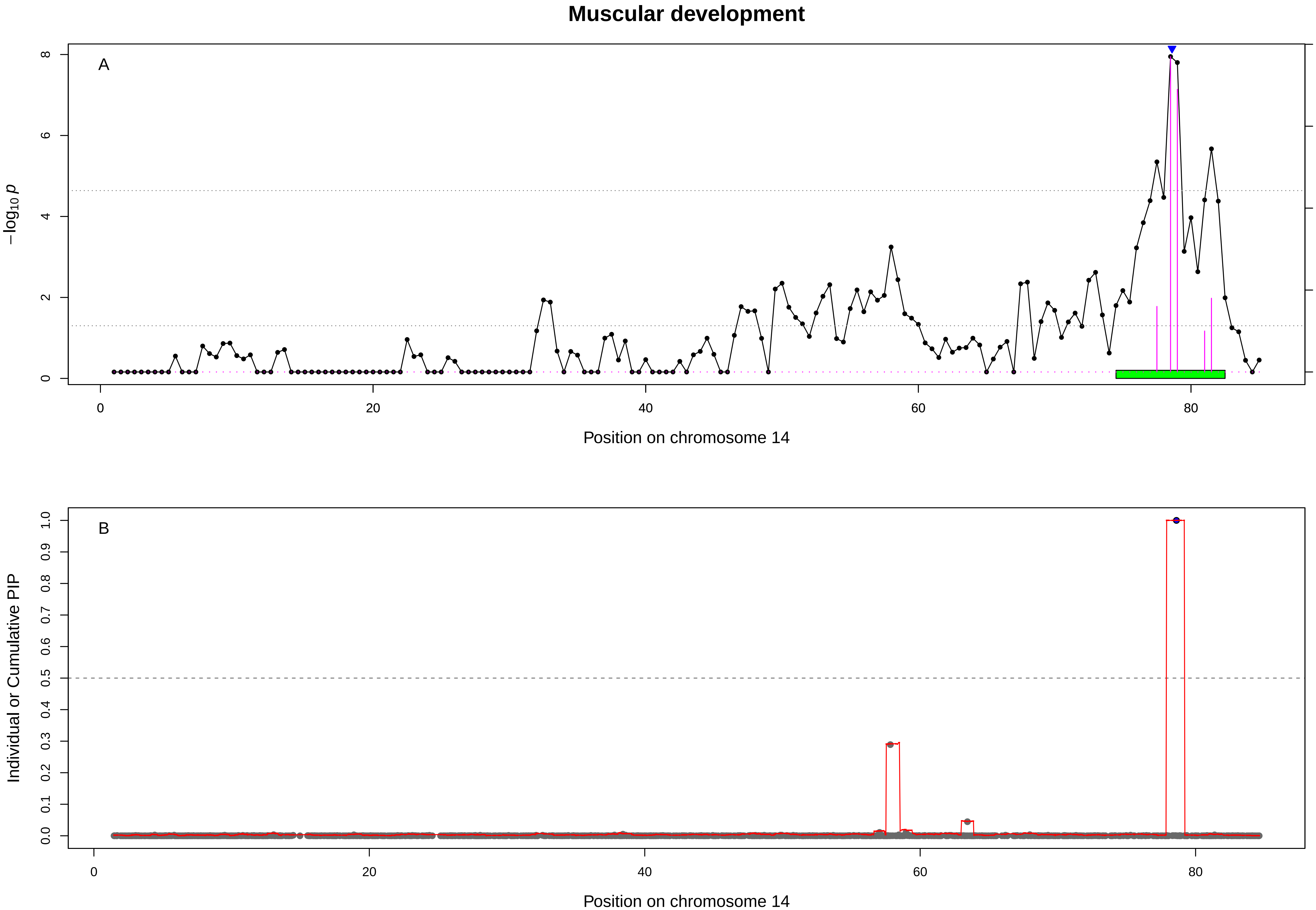

Muscular development
A
−log10 p
Position on chromosome 14
B
Individual or Cumulative PIP
Position on chromosome 14

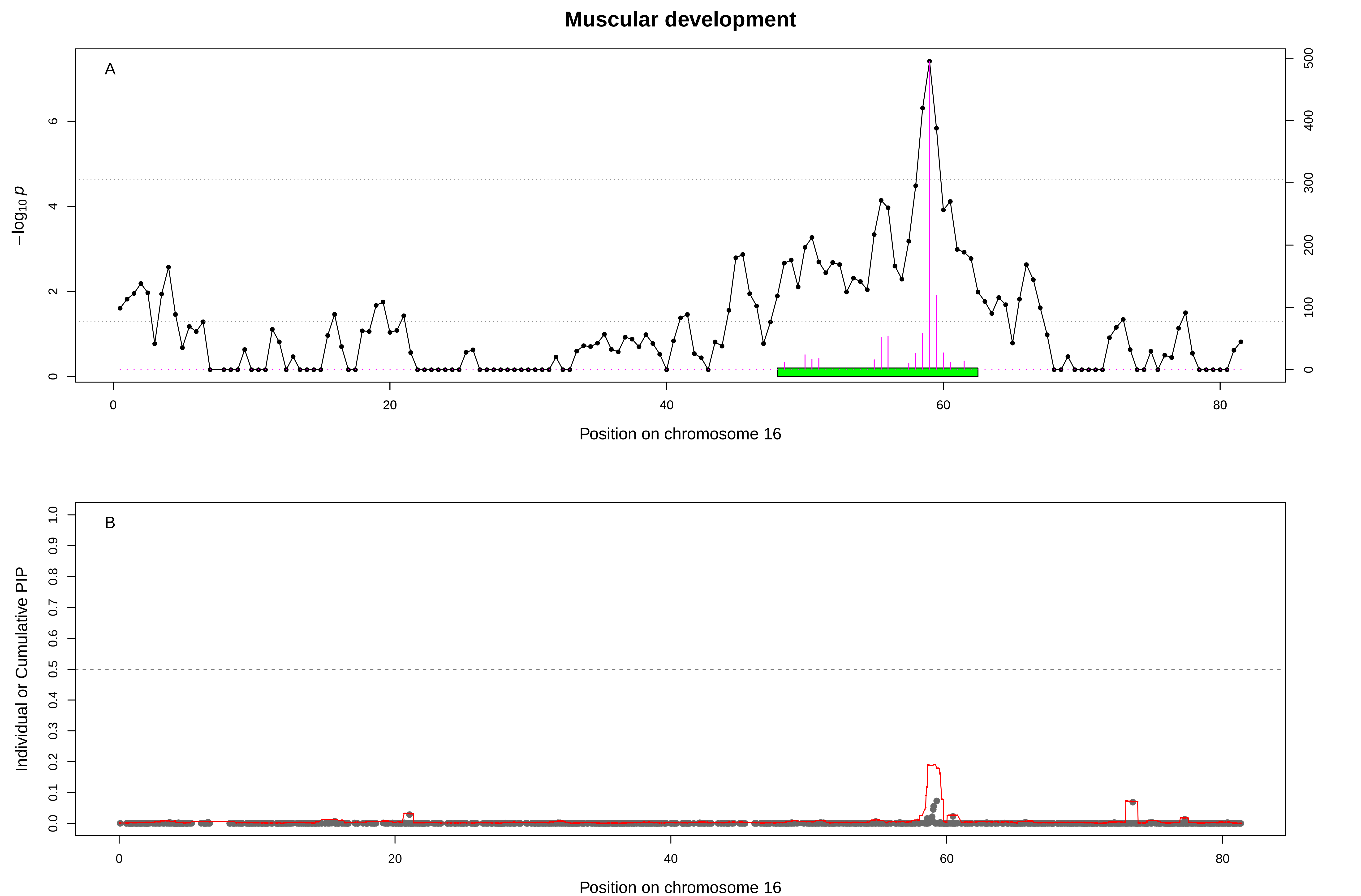
Muscular development
A
−log10 p
Position on chromosome 16
B
Individual or Cumulative PIP
Position on chromosome 16

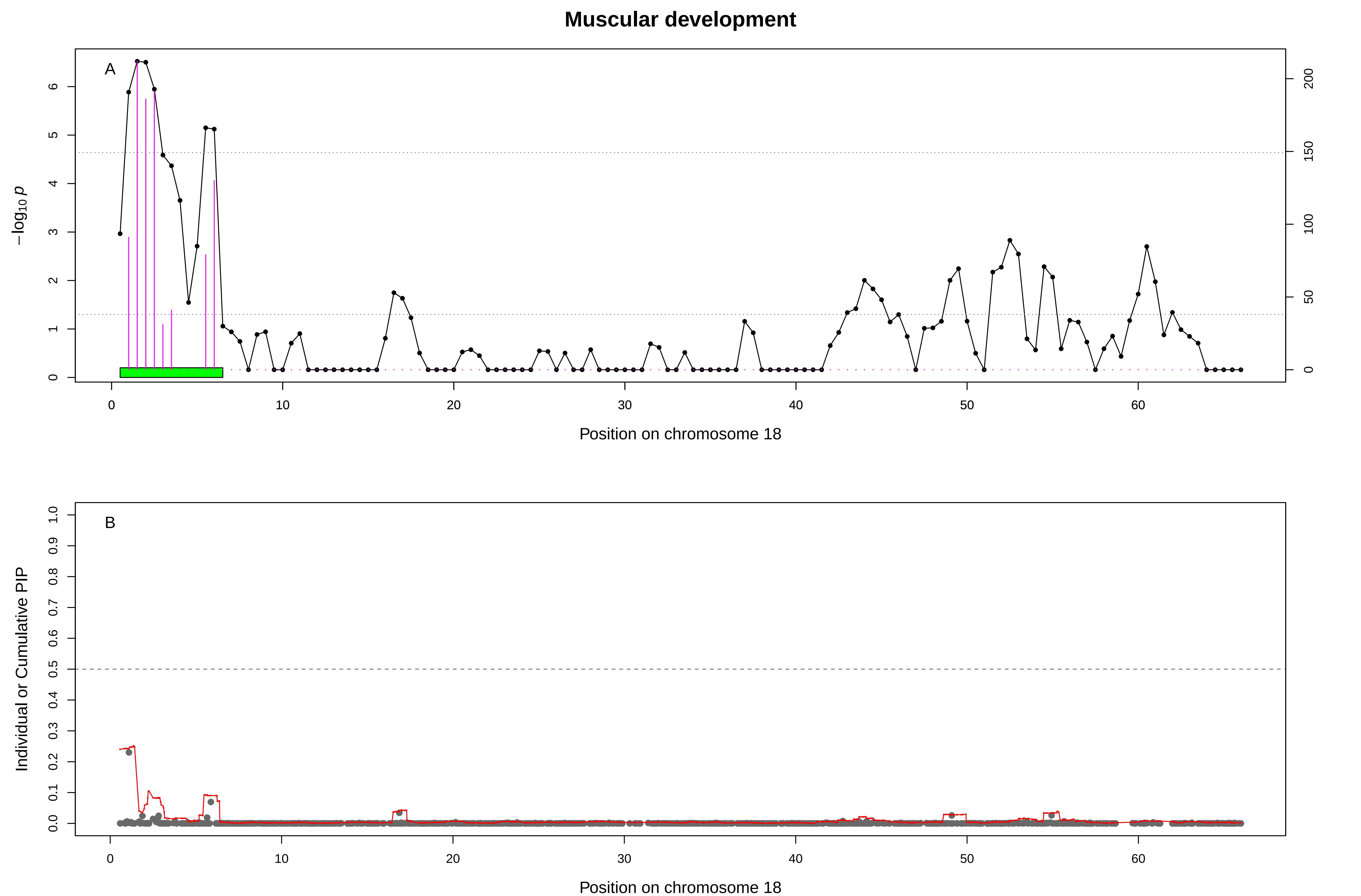

Muscular development
A
−log10 p
Position on chromosome 18
B
Individual or Cumulative PIP
Position on chromosome 18

Muscular development

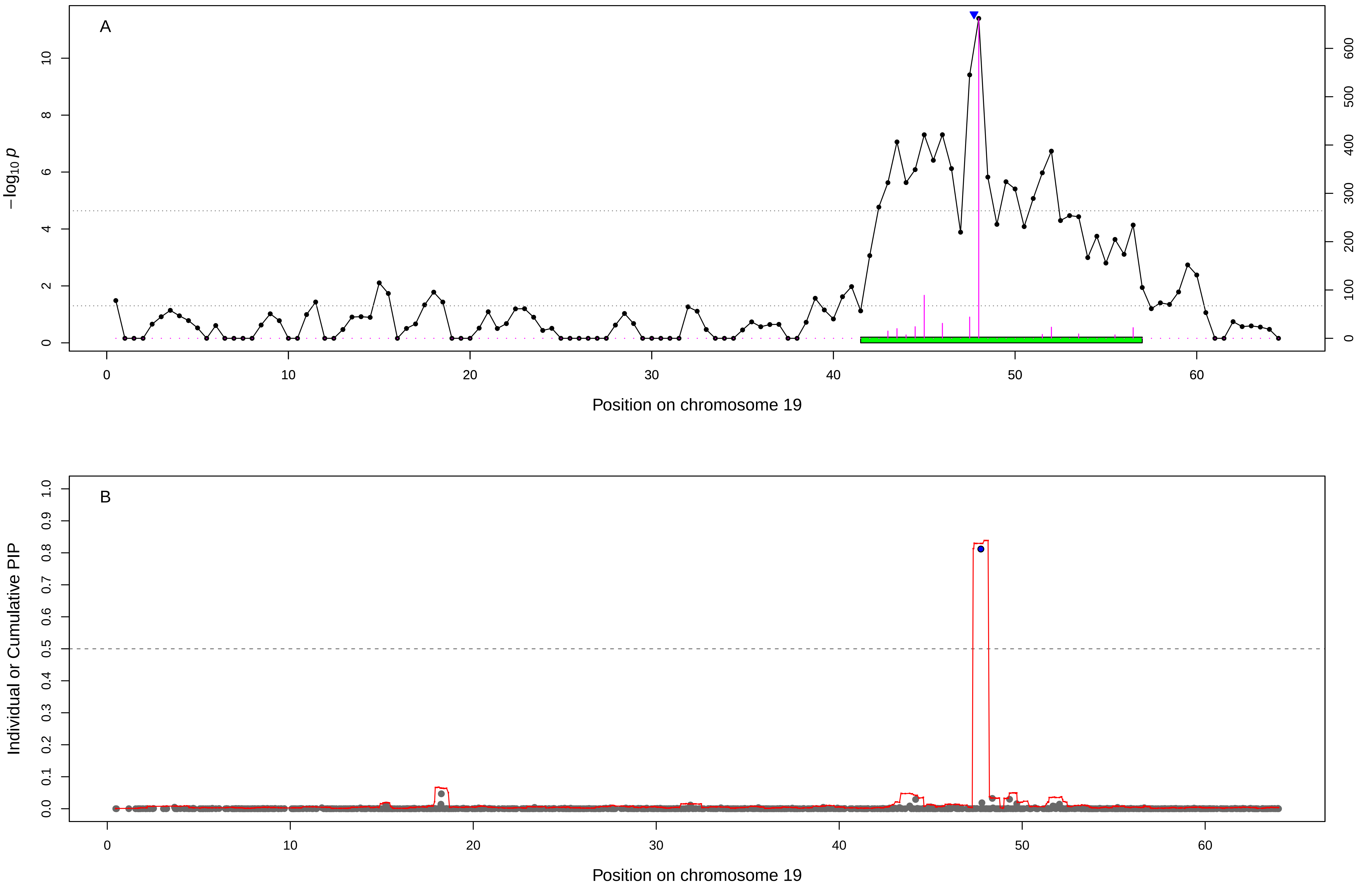

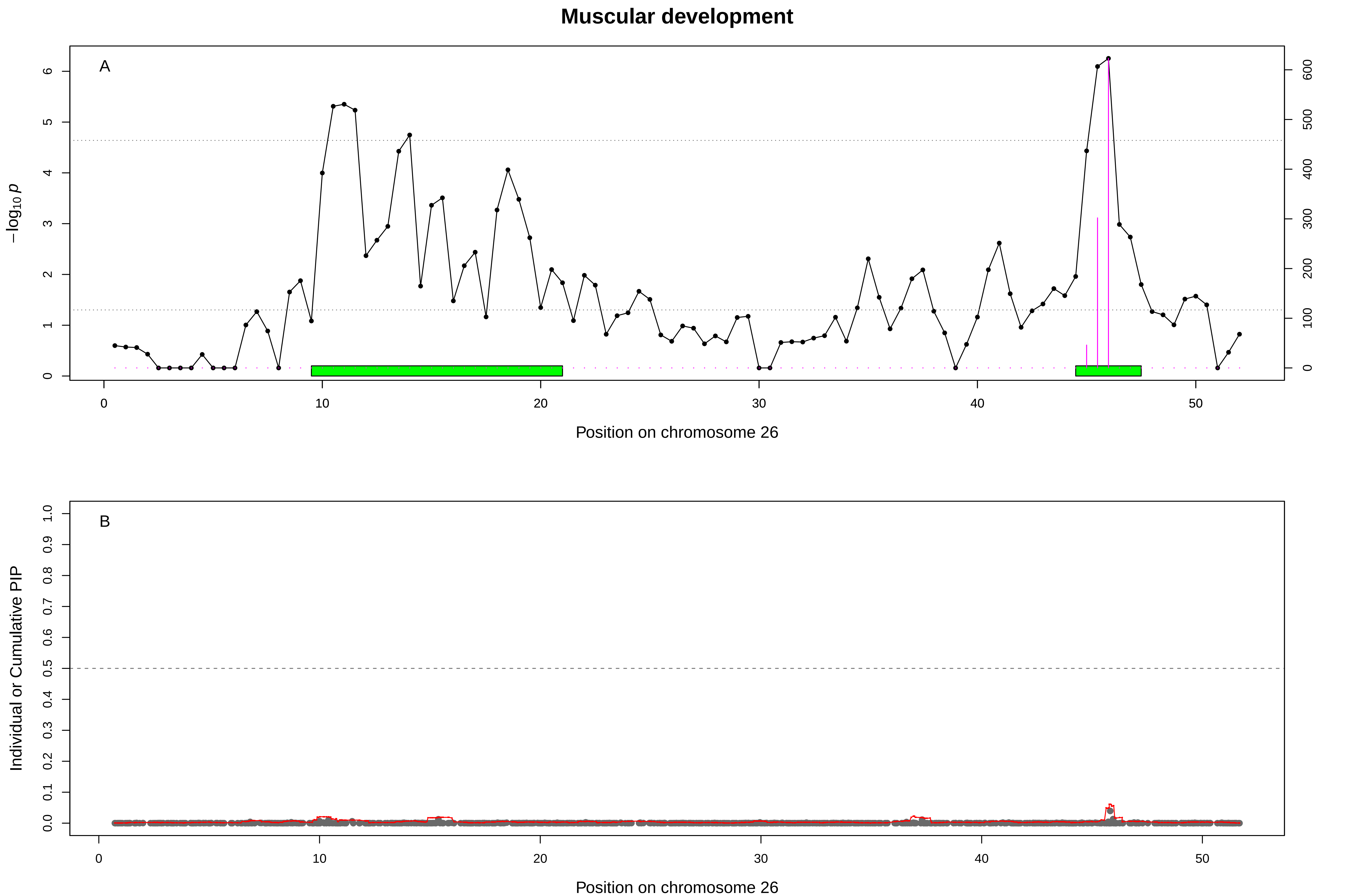
Muscular development
A
B
−log10 p
Individual or Cumulative PIP
Position on chromosome 26
Position on chromosome 26
0
1
2
3
4
5
6
0
100
200
300
400
500
600
0.0
0.1
0.2
0.3
0.4
0.5
0.6
0.7
0.8
0.9
1.0
0
10
20
30
40
50
